# Tissue-specific RNA Polymerase II promoter-proximal pause release and burst kinetics in a *Drosophila* embryonic patterning network

**DOI:** 10.1186/s13059-023-03135-0

**Published:** 2024-01-02

**Authors:** George Hunt, Roshan Vaid, Sergei Pirogov, Alexander Pfab, Christoph Ziegenhain, Rickard Sandberg, Johan Reimegård, Mattias Mannervik

**Affiliations:** 1 Department Molecular Biosciences, The Wenner-Gren Institute, Stockholm University, Stockholm, Sweden; 2 Department Cell and Molecular Biology, Karolinska Institutet, Stockholm, Sweden; 3 Department Cell and Molecular Biology, National Bioinformatics Infrastructure Sweden, Science for Life Laboratory, Uppsala University, Uppsala, Sweden

## Abstract

**Background:**

Formation of tissue-specific transcriptional programs underlies multicellular development, including dorsoventral (DV) patterning of the *Drosophila* embryo. This involves interactions between transcriptional enhancers and promoters in a chromatin context, but how the chromatin landscape influences transcription is not fully understood.

**Results:**

Here we comprehensively resolve differential transcriptional and chromatin states during *Drosophila* DV patterning. We find that RNA Polymerase II pausing is established at DV promoters prior to zygotic genome activation (ZGA), that pausing persists irrespective of cell fate, but that release into productive elongation is tightly regulated and accompanied by tissue-specific P-TEFb recruitment. DV enhancers acquire distinct tissue-specific chromatin states through CBP-mediated histone acetylation that predict the transcriptional output of target genes, whereas promoter states are more tissue-invariant. Transcriptome-wide inference of burst kinetics in different cell types revealed that while DV genes are generally characterized by a high burst size, either burst size or frequency can differ between tissues.

**Conclusions:**

The data suggest that pausing is established by pioneer transcription factors prior to ZGA and that release from pausing is imparted by enhancer chromatin state to regulate bursting in a tissue-specific manner in the early embryo. Our results uncover how developmental patterning is orchestrated by tissue-specific bursts of transcription from Pol II primed promoters in response to enhancer regulatory cues.

**Supplementary Information:**

The online version contains supplementary material available at 10.1186/s13059-023-03135-0.

## Introduction

**Fig. 1:**
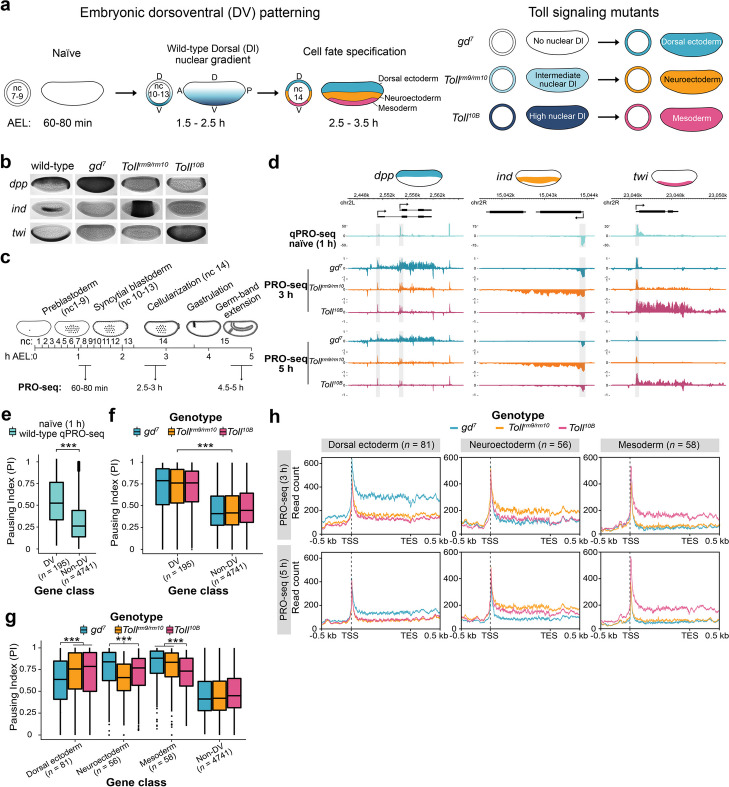
Promoter-proximal paused Pol II is established at DV-regulated genes prior to ZGA but is released into elongation in a tissue-specific manner.** a** Schematic of embryonic DV patterning. From an initially transcriptionally inert naïve embryo (nuclear cycle (nc) 7–9, 60–80 min (min) after egg laying (AEL)), a dorsoventral (DV) nuclear gradient of the maternally supplied transcription factor Dorsal (Dl) (nc 10–13, 1.5–2.5 hours (h) AEL) specifies cell fates at zygotic genome activation (ZGA) (nc 14, 2.5–3.5 h AEL). Distinct transcriptional programs initiated by the absence of Dl dorsally, moderate nuclear Dl laterally, and high nuclear Dl ventrally lead to cell specification into dorsal ectoderm, neuroectoderm, and mesoderm, respectively. Disrupted Dl gradient formation in *Toll* signaling mutants produces embryos composed entirely of presumptive dorsal ectoderm (*gd*^*7*^), neuroectoderm (*Toll*^*rm9/rm10*^), and mesoderm (*Toll*^*10B*^). **b** Images of whole-mount in situ hybridization in wild-type and *Toll* mutant embryos (2–4 h AEL) with probes hybridized to mRNAs of representative DV-regulated genes (*dpp*, *ind*, and *twi*). **c** Schematic of the experimental design to study spatio-temporal transcriptional dynamics during DV patterning. PRO-seq was performed on naïve wild-type embryos (nc 7–9, 60–80 min AEL) and *Toll* mutant embryos at ZGA (nc 14, 2.5–3 h AEL) and after gastrulation (> nc 14, 4.5–5 h AEL). **d** Genome browser shots of stranded PRO-seq signal (RPKM ×10^3^) at *dpp*, *ind*, and *twi*. Promoters are shaded gray. **e** Pausing index (PI) of DV and non-DV-regulated genes from qPRO-seq in wild-type naïve (1 h) embryos and **f** PRO-seq in *Toll* mutants. **g** PI of DV-regulated genes partitioned by the tissue of expression from PRO-seq in *Toll* mutants. **h** Metagene plots of *Toll* mutant PRO-seq signal at DV-regulated genes. Significant differences in the PI between DV and non-DV genes are from the Wilcoxon rank-sum and signed-rank tests and indicated by asterisks, * = *P* < 0.05, ** = *P* < 0.01, *** = *P* < 0.001

The ability to dynamically regulate gene expression is integral to developmental processes in multicellular organisms by enabling cells that retain identical DNA sequences to form specialized cell types. Early *Drosophila* embryogenesis involves 13 rapid, synchronous nuclear divisions within a syncytium to give rise to ~6000 nuclei that then cellularize, undergo zygotic genome activation (ZGA), and become specified. Dorsoventral (DV) axis specification of the early *Drosophila* embryo is one of the most well studied gene regulatory networks (reviewed in [1, 2]). During DV patterning, distinct cell fates form in response to an intranuclear morphogen gradient of the maternally supplied REL-family transcription factor Dorsal (Dl) [3–5]. Differential activation of Toll receptors leads to high nuclear import of Dl in ventral regions, low levels of nuclear Dl in lateral regions and an absence of Dl in dorsal regions (reviewed in [6]). The Dl gradient forms during nuclear cycles 10–14 and induces distinct complements of zygotic genes in ventral, lateral, and dorsal regions of the embryo, leading to cell specification at nuclear cycle 14 and formation of presumptive mesoderm, neurogenic ectoderm, and dorsal ectoderm, respectively (Fig. 1A). Dl activates genes such as *twist* (*twi*) in the mesoderm and *intermediate neuroblasts defective* (*ind*) in the neuroectoderm, but can also function as a repressor, which restricts genes such as *decapentaplegic* (*dpp*) to the dorsal ectoderm where Dl is absent from the nuclei (Fig. 1B).

An important aspect of transcriptional regulation is how regulatory signals are conveyed from enhancers to elicit a transcriptional response at the promoter. Hi-C, Micro-C, and microscopy-based data revealed that there are no differences in the topologically associated domain (TAD) structure or enhancer-promoter (E-P) contact frequencies for DV genes between cells in the embryo where they are expressed or silent [7–9]. This suggests that E-P looping is not the step that triggers tissue-specific activation of DV genes. Pausing of transcriptionally engaged RNA Polymerase II (Pol II) 30–60 bp downstream of the transcription start site (TSS) has been identified as an important regulatory checkpoint that allows the release of Pol II into productive elongation to be tightly controlled (reviewed in [10, 11]). Pol II pausing is prevalent among developmental genes during *Drosophila* embryogenesis [12] and allows cells in a tissue to synchronously activate gene expression [13].

DV tissue mutant embryos, derived from maternal effect mutations, with either the absence (*gd*^*7*^, dorsal ectoderm), or uniformly low (*Toll*^*rm9/rm10*^, neurogenic ectoderm) and high (*Toll*^*10B*^, mesoderm) levels of nuclear Dl (Fig. 1a,b), provided an amenable substrate for ChIP-based approaches to characterise DV enhancers and other important regulatory elements based on the enrichment of histone modifications such as H3K27ac and occupancy of the co-activator CBP [9, 14–18]. Nonetheless, a comprehensive genome-wide assessment of the interplay between transcriptional activity and chromatin state across the DV axis is lacking.

In this study, we used the DV patterning model to examine the spatio-temporal interplay between transcription and chromatin state. We performed Precision Run-On Sequencing (PRO-seq) on precisely aged tissue mutant *Drosophila* embryos to measure nascent transcription and Pol II pausing genome-wide, alongside chromatin state data from ATAC-seq, ChIP-seq, and CUT&Tag. We further inferred transcriptional burst kinetics from single-cell RNA-seq data. Our findings suggest that enhancers and promoters are initially primed for activation competency across cells that adopt distinct fates, but the spatio-temporally regulated acquisition of distinct patterns of enhancer CBP occupancy and histone acetylation in response to the Dl gradient leads to differential DV gene expression by controlling burst kinetics and the release of paused Pol II into productive elongation.

## Results

### Paused Pol II is established at dorsoventral genes prior to their expression in the early embryo

To obtain a precise genome-wide assessment of the activity state of Pol II and spatio-temporal differences in zygotic transcription during DV patterning, we performed PRO-seq on naïve wild-type embryos, 60–80 min after egg laying (AEL), and on DV tissue mutant embryos composed entirely of presumptive dorsal ectoderm (*gd*^*7*^), neurogenic ectoderm (*Toll*^*rm9/rm10*^), or mesoderm (*Toll*^*10B*^) at 3 and 5 h AEL (Fig. 1a–d) [19]. For the naïve stage, we also hand-sorted embryos to ensure that they were not older than nuclear cycle (nc) 9, and used the more sensitive qPRO-seq protocol [20]. We identified differentially expressed genes between the mutant embryos by comparing the number of PRO-seq reads mapping to the gene body (defined as the coding DNA sequence (CDS) of the gene), and observed 195 genes that were upregulated specifically in one of the mutants (Additional file 1: Fig. S1a,b and Additional file 2: Table S1). A comparison with previously published DV-regulated genes [21] showed a large overlap and expression in the expected tissue (Additional file 1: Fig. S1c,d). Gene ontologies for the differentially expressed genes were consistent with their expected functions in epithelial, nervous system, and muscle development, respectively (Additional file 2: Table S2). Most DV-regulated genes were expressed at both 3 and 5 h AEL, but some were specific to the later time point (Additional file 1: Fig. S1e, Additional file 2: Table S1).

Many developmental genes exhibit promoter-proximal paused RNA polymerase II (Pol II) ~30–60 bp downstream of the TSS [22]. To measure pausing, we calculated the pausing index from the ratio of PRO-seq reads mapping to the promoter (from 50 bp upstream of the TSS to 100 bp downstream of the TSS) and the sum of reads mapping to the promoter and the gene body, which revealed that DV genes, as well as anterior-posterior (AP) patterning genes, were more highly paused than non-DV genes expressed in these embryos (Fig. 1e,f, Additional file 1: Fig. S1f). Interestingly, Pol II pausing was observed at DV genes already in the naïve stage, prior to their expression (Fig. 1d,e).

To ensure that detection of paused Pol II in the naïve stage was not due to sample contamination with older embryos, we measured the gene body read counts and pausing index of zygotic genes expressed at specific stages of development [23] (Additional file 1: Fig. S1g-j). Genes already expressed at nc 7–9 and nc 9–10 had higher gene body qPRO- and PRO-seq signal than DV genes and genes expressed at the syncytial (nc 11–13) and cellularized (nc 14) blastoderm stages, demonstrating that the experiments captured properly staged embryos (Additional file 1: Fig. S1g-i). Whereas DV genes were paused at the naïve stage, genes expressed at the naïve stage had a low pausing index, consistent with previous findings [24] (Additional file 1: Fig. S1j). Core promoter motifs have been shown to strongly influence Pol II recruitment and pausing [10, 25, 26]. Examination of the CORE database [27] and de novo motif analysis showed that DV genes were highly enriched for core promoter motifs [28], such as Initiator (Inr), downstream promoter element (DPE), and TATA-box, compared to other genes (Additional file 1: Fig. S1k-n and Additional file 3: Table S3), likely contributing to their high pausing index.

High Pol II pausing was maintained at dorsal ectoderm, neuroectoderm, and mesoderm-specific genes across all three DV mutants (Fig. 1f), but gene body reads were elevated in specific mutants, as exemplified by *decapentaplegic* (*dpp)*, *intermediate neuroblasts defective (ind)*, and *twist* (*twi)* (Fig. 1d). Similar results were obtained with Pol II antibodies in CUT&Tag on *Toll* mutant embryos (Additional file 1: Fig. S1o). The pausing index for DV genes was significantly lower in the tissue mutant of expression (Fig. 1g). To address whether the reduction in pausing was due to the elevated gene body reads in the tissue of expression, or a decrease in reads for promoter-proximal paused Pol II, we measured the signal for these regions separately for all genes (Additional file 1: Fig. S1p, Additional file 2: Table S1) and generated metaplots of PRO-seq read density (Fig. 1h). The promoter-proximal Pol II signal was similar among the three mutants for most genes at 3 h (AEL), and the reduced pausing index was mostly explained by the elevation of gene body reads, suggesting a key role for pause release in DV gene transcription. The observation that DV genes become highly paused in naïve embryos prior to their transcription and that pausing is maintained in different tissue contexts, irrespective of transcription, confirms that pause release is a major regulatory step in tissue-specific DV transcription [12].

### Enhancer chromatin state reflects tissue-specific DV gene transcription

**Fig. 2:**
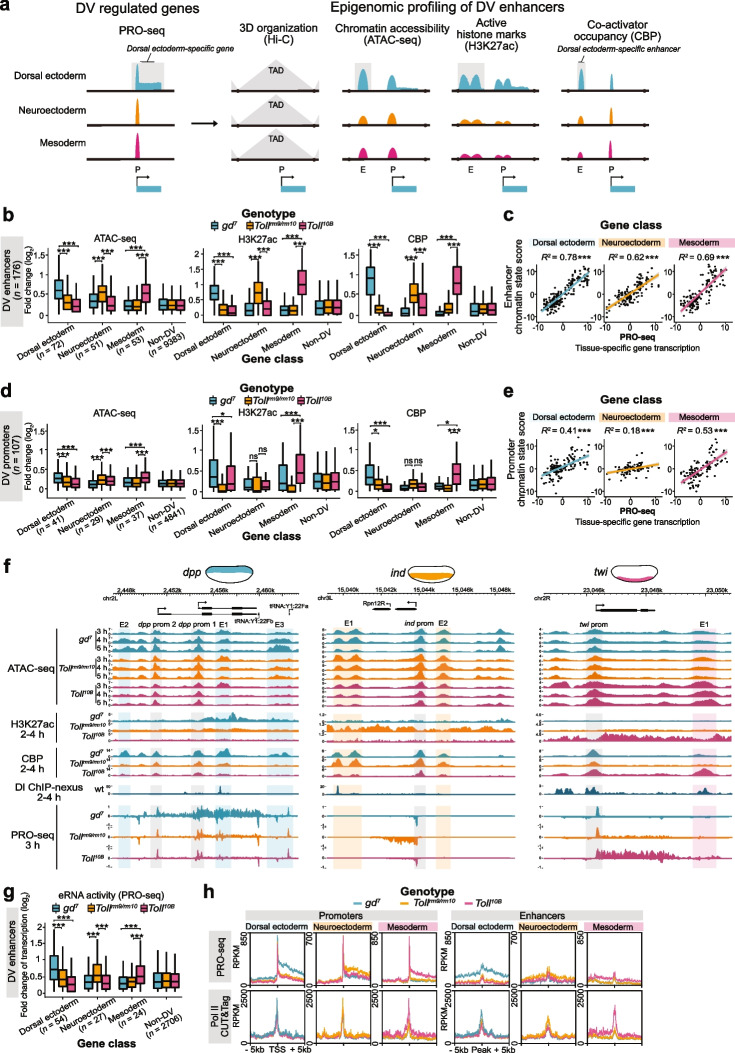
Epigenomic profiling identifies chromatin states at DV enhancers and promoters that correlate with tissue-specific gene expression.** a** Schematic of the epigenomic profiling strategy for identifying tissue-specific DV enhancers genome-wide. PRO-seq identified DV genes were linked to regions within the same topologically associating domain (TAD) with differential chromatin accessibility (ATAC-seq), enrichment of the active histone mark H3K27ac, and occupancy of CBP between *Toll* mutants. **b** The fold change (log_2_) in ATAC-seq and CBP and H3K27ac [9, 14, 15] ChIP-seq tissue-specific enrichment scores from *Toll* mutant embryos at DV (dorsal ectoderm *n* = 72, neuroectoderm *n* = 51, mesoderm *n* = 53) and non-DV (*n* = 9383) enhancers. Significant differences in enrichment from the Wilcoxon signed-rank test are indicated by asterisks, * = *P* < 0.05, ** = *P* < 0.01, *** = *P* < 0.001. **c** Correlations of the combined tissue-specific enhancer chromatin state score and target DV gene tissue-specific transcription. The coefficient of determination (*R*^*2*^) is shown alongside asterisks denoting the associated *P*-value significance, * = *P* < 0.05, ** = *P* < 0.01, *** = *P* < 0.001. **d** The fold change (log_2_) in tissue-specific enrichment scores from *Toll* mutants for the genomic datasets in **b** at promoters associated with DV enhancers (dorsal ectoderm *n* = 41, neuroectoderm *n* = 29, mesoderm *n* = 37). **e** Correlations of the combined tissue-specific promoter chromatin state score and target DV gene tissue-specific transcription. **f** Genome browser shots of *Toll* mutant ATAC-seq (3, 4, and 5 h AEL), H3K27ac and CBP ChIP-seq (2–4 h AEL) and PRO-seq (3 h) alongside Dl ChIP-nexus (2–4 AEL wild-type embryos) [33] at *dpp*, *ind*, and *twi*. The genomic position of DV enhancers and promoters are denoted. **g** Boxplots showing the fold change (log_2_) in enhancer RNA (eRNA) activity measured from *Toll* mutant PRO-seq at DV and non-DV enhancers. **h** Metagene profiles of *Toll* mutant PRO-seq (3 h) and Pol II (Rpb3) CUT&Tag (2–4 h AEL) signal (RPKM) at DV enhancers (± 5 kb of CBP) and promoters (± 5 kb of TSS)

To identify what controls the release of paused Pol II into productive elongation, we examined the chromatin states of enhancers and promoters for DV genes. Occupancy of p300/CBP and enrichment of the p300/CBP-catalyzed mark H3K27ac are hallmarks of active enhancers [29–32], and DV enhancers have previously been identified based on differential H3K27ac [9, 15]. We generated chromatin accessibility (ATAC-seq) and *Drosophila* CBP (Nejire) ChIP-seq data from DV mutant embryos and screened for DV enhancers by correlating differential expression with genomic regions that exhibit tissue-specific H3K27ac enrichment [9, 14, 15], CBP occupancy, and chromatin accessibility (Fig. 2a).

We assigned genomic regions with differential occupancy and accessibility to target genes within the same topologically associated domain (TAD), and identified 176 putative DV enhancers linked to 107 promoters (Additional file 1: Fig. S2a,b and Additional file 4: Table S4). Most genes were associated with one or two DV enhancers, but a few genes had multiple enhancers (Additional file 1: Fig. S2c). Examining the distribution of enhancer-TSS genomic distances revealed a subset of promoter-proximal enhancers, but the majority of enhancers (82%) were distal (> 700 bp) to their targets (Additional file 1: Fig. S2d). DV enhancers showed a characteristic pattern of H3K27ac flanking the central maxima of CBP enrichment and region of accessible chromatin (Additional file 1: Fig. S2e,f) that likely reflects CBP recruitment by DNA-binding TFs (Additional file 1: Fig. S2e,f). We validated our enhancer identification strategy by examining overlapping genomic regions tested in a high-throughput transgenic reporter gene assay [34], which revealed the enrichment of annotation terms associated with dorsal ectoderm expression for *gd*^*7*^ enhancers, ventral ectoderm for *Toll*^*rm9/rm10*^ enhancers, and mesoderm for *Toll*^*10B*^ enhancers (Additional file 1: Fig. S2g). Examples of regions overlapping DV enhancers tested in reporter assays that recapitulate the expected spatial expression patterns are shown in Additional file 1: Fig. S2h [34]. We conclude that chromatin state data is highly efficient in identifying tissue-specific enhancers, validating previous results [9, 14, 15].

We categorized enhancers based on the tissue-specific expression of their target genes and observed high tissue specificity of elevated chromatin accessibility, CBP, and H3K27ac (Fig. 2b and Additional file 1: Fig. S2i). A chromatin state enhancer score from the combined tissue-specific signal for CBP, H3K27ac, and ATAC-seq could accurately predict the level of tissue-specific expression determined by PRO-seq (Fig. 2c, R^*2*^ values 0.78, 0.62, 0.69 for dorsal ectoderm, neuroectoderm, and mesoderm enhancers, respectively), and had higher predictive value combined than individually (Additional file 1: Fig. S2j). The chromatin state of DV promoters varied less across tissues and predicted the expression of target genes less accurately (Fig. 2d,e, Additional file 1: Fig. S2j,k).

In summary, the data suggest that whereas DV enhancer chromatin state correlates with tissue-specific expression, the promoter chromatin state is more tissue-invariant and may allow recruitment and establishment of paused Pol II to prime DV promoters for transcription in all three germ layers. This is consistent with a model where promoter-bound CBP supports Pol II recruitment and pausing without inducing H3K27ac (Fig. 2f) [35], whereas catalytic CBP activity at enhancers is critical for tissue-specific histone acetylation and release from pausing.

### Temporal changes to enhancer accessibility correlate with variations in DV expression

To explore spatio-temporal accessibility dynamics during the induction of DV-responsive transcription, we analyzed our *Toll* mutant ATAC-seq data from three time points (3, 4, and 5 h AEL) (Additional file 1: Fig. S3a). ATAC-seq revealed that tissue-specific accessibility at DV enhancers became more pronounced from 3 to 5 h (Additional file 1: Fig. S3a). Chromatin accessibility at DV promoters also increased from 3 to 5 h, but with less tissue specificity (Additional file 1: Fig. S3a). We quantified changes in accessibility across the time course for each DV enhancer, specifically in the tissue mutant where its target gene is expressed, and identified enhancers that gained (log_2_ fold change ≥ 0.5), lost (log_2_ fold change ≤ −0.5), or maintained stable accessibility (Additional file 1: Fig. S3b). While the majority of enhancers gained or maintained accessibility over time in the tissue of expression, a subset lost accessibility (Additional file 1: Fig. S3b,c). Measuring the PRO-seq gene body expression at early (2.5–3 h) and late (4.5–5 h) phases of DV-responsive transcription revealed genes linked to enhancers that gained accessibility had significantly stronger expression at the later time point whereas genes associated with enhancers that lost accessibility had significantly weaker expression at the later phase compared to the earlier phase (Additional file 1: Fig. S3d).

The closing down of specific enhancers for a gene may indicate transfer of regulatory control between enhancers that drive expression at different developmental stages. For example, at the locus of the dorsal ectoderm-specific gene *schnurri* (*shn*), enhancers linked to expression at early (E1) and late (E2) developmental stages undergo temporal changes in accessibility that correlate with the spatio-temporal pattern of *shn* expression (Additional file 1: Fig. S3e-g). The E1 enhancer is primed by chromatin accessibility through nc 11–13 [36], and is initially accessible in all the tissue mutants at the start of nc 14 (3 h), but rapidly closes down in *Toll*^*rm9/rm10*^ and *Toll*^*10B*^ embryos at 4 h, and in *gd*^7^ embryos at 5 h (Additional file 1: Fig. S3f,g). The upstream E2 enhancer gains accessibility specifically in *gd*^7^ embryos from 4 h onwards, suggesting regulatory control of *shn* is transferred from E1 to E2 as development proceeds (Additional file 1: Fig. S3g). Supporting this, reporter gene activities driven by fragments overlapping E1 and E2 have distinct spatial and temporal patterns that recapitulate the early and later embryonic expression patterns of *shn*, respectively (Additional file 1: Fig. S3g) [34]. These results are consistent with previous findings [37], and with the enhancer rather than promoter chromatin state driving tissue-specific DV transcription.

### Enhancer RNAs are more abundant in the tissue of expression

In mammals, non-coding transcription is a predictive marker of active enhancers [38, 39]. Enhancer RNAs (eRNAs) may allosterically activate the HAT activity of p300/CBP [40] (but see also [41]) and are implicated in supporting the transition of paused Pol II into elongation [42]. *Drosophila* eRNA transcription also correlates with enhancer activity [43], but direct comparisons of eRNA levels between the same enhancer in active and inactive cellular contexts are lacking. From the PRO-seq signal at intergenic enhancers and the non-coding strand of genic enhancers, we detected eRNAs that were more abundant in the tissue where the target gene was expressed (Fig. 2g, Additional file 4: Table S4). For example, at the intronic *dpp* E1 enhancer, we detected an eRNA with strong antisense transcription specific to *gd*^*7*^ embryos (Additional file 1: Fig. S2l). Interestingly, Pol II CUT&Tag enrichment at DV enhancers was strong, whereas the PRO-seq signal that captures transcriptionally engaged Pol II was low compared to promoters (Fig. 2h). It therefore appears that Pol II is efficiently recruited to both promoters and enhancers, but that Pol II engages in transcription to a lesser extent at enhancers. This suggests that features specific to enhancers and promoters are involved in establishing transcriptionally engaged Pol II at a post-recruitment step.

### DV transcription occurs within the context of a tissue-invariant chromatin conformation

Early *Drosophila* embryogenesis involves the rapid formation of an elaborate 3D chromatin organization characterized by the establishment of TADs and the formation of enhancer-promoter loops [8, 44]. Although TAD formation coincides with ZGA, it occurs independently of transcription and is tissue-invariant and gene expression is largely unaltered by major disruptions of chromosome topology [9, 44, 45]. Enhancer-promoter loops are also maintained across tissues in the early embryo [7–9, 44, 46], so although these loops are important for positioning enhancers and promoters in proximity to each other, additional regulatory components are required to drive tissue-specific expression. Consistently, despite the major differences in chromatin state and transcription, the genome organization of the DV-regulated genes *dpp*, *ind*, and *twi* appear largely tissue-invariant between *Toll* mutants (Additional file 1: Fig. S3h) [9].

### Tissue-specific P-TEFb recruitment releases Pol II into productive elongation at DV genes

**Fig. 3:**
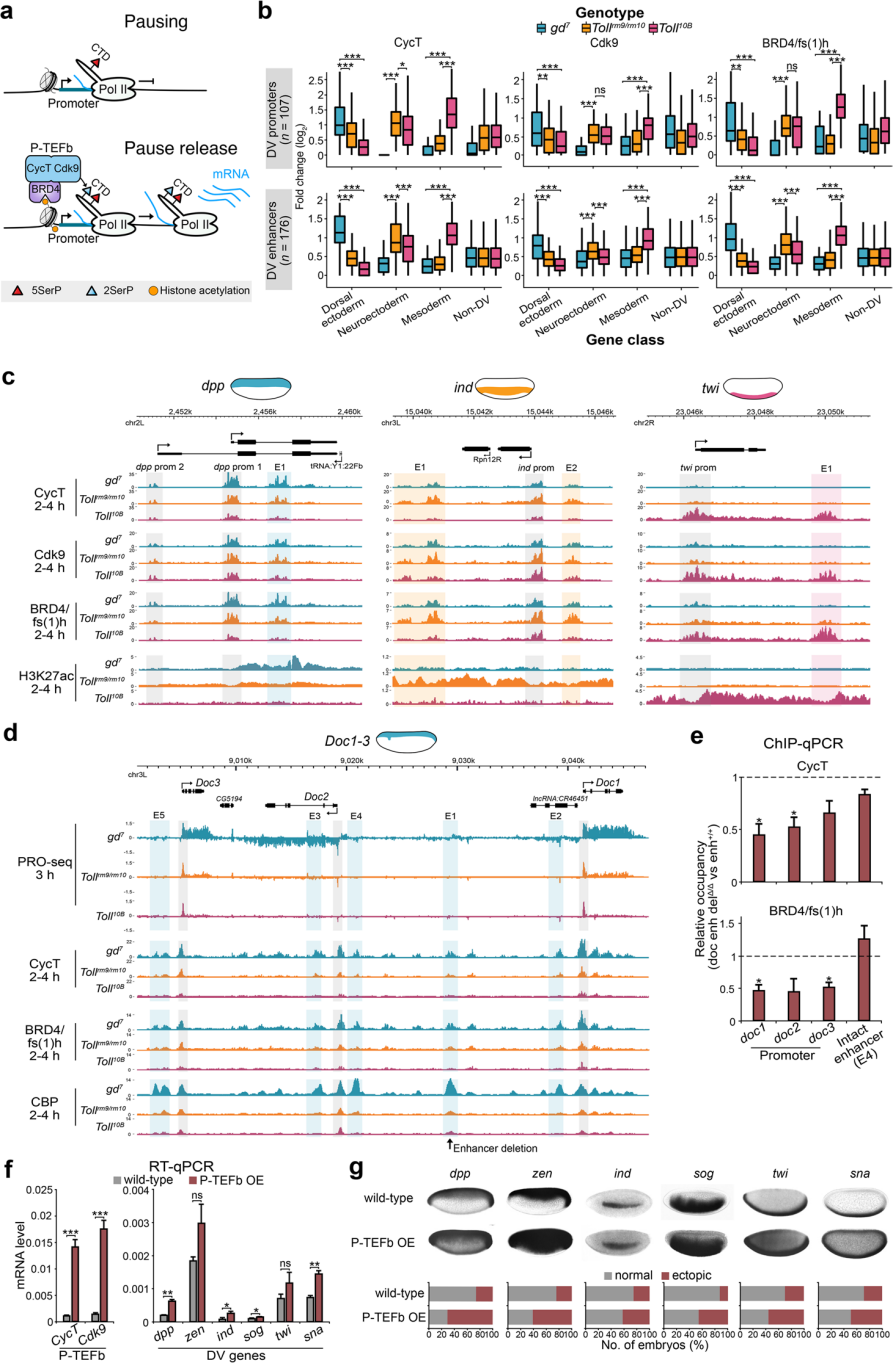
Tissue-specific P-TEFb recruitment is associated with the release of paused Pol II into productive elongation at DV promoters. **a** Schematic of P-TEFb (composed of CycT and CDK9 subunits) mediated release of promoter-proximal paused Pol II into productive elongation. P-TEFb phosphorylates serine 2 of the Pol II carboxyl-terminal domain (CTD) to stimulate elongation. BRD4/fs(1)h binds to acetylated histones and helps recruit P-TEFb. **b** The fold change (log_2_) in CycT, Cdk9, and BRD4/fs(1)h CUT&Tag tissue-specific enrichment scores from *Toll* mutant embryos at DV promoters and enhancers. Significant differences in enrichment are from the Wilcoxon signed-rank test and indicated by asterisks, * = *P* < 0.05, ** = *P* < 0.01, *** = *P* < 0.001. **c** Genome browser shots of *Toll* mutant CycT, Cdk9 and BRD4/fs(1)h CUT&Tag, and H3K27ac ChIP-seq at *dpp*, *ind*, and *twi* and **d** *Toll* mutant PRO-seq, CBP ChIP-seq, and CycT and BRD4/fs(1)h CUT&Tag at the *Doc* locus. The position of the *Doc* E1 enhancer deletion [7] is denoted. **e** ChIP-qPCR enrichment of CycT and BRD4/fs(1)h at the *Doc1-3* promoters and the intact E4 enhancer in *Doc enh del*^*Δ/Δ*^ embryos (2–4 h AEL) relative to enh^+/+^ embryos (*n* = 3–4). Error bars show SEM. Significant differences in occupancy (two tailed, unpaired *t*-test) are indicated by asterisks (* = *P* < 0.05). **f** RT-qPCR quantification of *CycT*, *Cdk9* and DV-regulated genes (*dpp*, *zen*, *ind*, *sog*, *twi*, and *sna*) mRNA levels (relative to 28S rRNA) in wild-type embryos and P-TEFb maternally overexpressed (OE) embryos. Error bars show SEM. Significant differences in mRNA (two tailed, unpaired *t*-test) are indicated by asterisks (* = *P* < 0.05, ** = *P* < 0.01, *** = *P* < 0.001). **g** (top) Images of whole-mount in situ hybridization in wild-type and P-TEFb OE mutant embryos (2–4 h AEL) with probes hybridized to mRNAs of the DV-regulated genes in **f** and (bottom) quantification of the proportion of embryos with ectopic signal for each probe. The number of embryos sampled is detailed in the methods

The tissue-invariant 3D topology and promoter chromatin state at DV loci suggest that the tissue-specific enhancer chromatin state may provide a signal to trigger the release of paused Pol II into elongation. A critical step in the release of paused Pol II is the phosphorylation of negative elongation factors and the Pol II C-terminal domain (CTD) by the P-TEFb kinase, which consists of CDK9 and Cyclin T (CycT) (Fig. 3a) [47, 48]. To establish whether tissue-specific activity of P-TEFb at DV genes is regulated by differential recruitment or post-recruitment enzymatic activation, we performed CycT and CDK9 CUT&Tag in 2–4 h *Toll* mutant embryos. This revealed that P-TEFb occupancy is more strongly associated with dorsal ectoderm promoters in *gd*^*7*^ embryos, neuroectoderm promoters in *Toll*^*rm9/rm10*^ embryos, and mesoderm promoters in *Toll*^*10B*^ embryos (Fig. 3b,c and Additional file 1: Fig. S4a), despite similar promoter chromatin accessibility between tissues (Fig. 2d). We validated this result by ChIP-qPCR, showing tissue-specific CycT enrichment at DV promoters (Additional file 1: Fig. S4b). Interestingly, we observed comparable levels of P-TEFb enrichment, and even higher tissue specificity at DV enhancers (Fig. 3b,c and Additional file 1: Fig. S4c). This suggests enhancer-binding factors may load P-TEFb and direct it to the target promoter. To test this, we investigated P-TEFb occupancy at the *Dorsocross* (*Doc*) locus that consists of three genes (*Doc1*, *Doc2*, and *Doc3*) and five enhancers (Fig. 3d). CycT was highly enriched at both enhancers and promoters in the dorsal ectoderm (*gd*^*7*^ embryos) compared to the other tissues. We examined CycT occupancy in embryos homozygous for a deletion of the *Doc* E1 enhancer [7]. Removal of this single enhancer marginally reduced expression of the *Doc* genes and had minimal effects on the chromatin state of the locus (Additional file 1: Fig. S4d,e), reflecting functional redundancy of the intact enhancers that maintain promoter contacts [7]. Nevertheless, by ChIP-qPCR, we could detect a reduction in the occupancy of CycT at the *Doc* promoters in embryos lacking the E1 enhancer (Fig. 3e), indicating that enhancers modulate loading of P-TEFb to promoters.

One factor that has been implicated in P-TEFb recruitment is the tandem bromo- and extra-terminal domain (BET) protein BRD4, known as female sterile (1) homeotic (fs(1)h) in *Drosophila* (Fig. 3a) [49, 50]. We performed BRD4/fs(1)h CUT&Tag and found that it is also more strongly associated with DV promoters and enhancers in the tissue of target gene expression (Fig. 3b–d, Additional file 1: Fig. S4a and c) and that its occupancy at the *Doc* promoters was reduced in the absence of the E1 enhancer (Fig. 3e). Although BRD4/fs(1)h can recognize acetylated histones through its bromodomains [51], occupancy was restricted to enhancers and promoters and did not overlap the more diffused H3K27ac pattern (Fig. 3c and Additional file 1: Fig. S4a). This indicates that other histone modifications or factors binding accessible chromatin at enhancers may be more important for BRD4/fs(1)h recruitment than H3K27ac.

Tissue-specific enrichment of P-TEFb suggests it may be limiting for transcription in non-expressing tissues. We therefore over-expressed Cdk9 and CycT in early embryos with the maternal tub-Gal4 driver, leading to more than 10-fold increased expression in embryos (Fig. 3f). Although occupancy of P-TEFb did not increase at tested promoters according to CycT ChIP-qPCR (Additional file 1: Fig. S4f), the expression of some DV genes was elevated (Fig. 3f). Interestingly, the number of embryos with DV expression detected outside the normal expression domain was significantly increased by P-TEFb overexpression for all DV genes examined by whole-mount in situ hybridization (Fig. 3g and Additional file 1: Fig. S4g). Furthermore, precocious activation of DV genes could be detected in these embryos (Additional file 1: Fig. S4h). Since promoter occupancy of P-TEFb did not change upon overexpression, ectopic expression may result from titration of negative regulators of P-TEFb, such as the 7SK snRNP that sequesters and inactivates the kinase [52]. Consistent with this idea, the frequency of ectopic expression correlated with the level of CycT at gene promoters in non-expressing tissues (*r* = 0.72, Additional file 1: Fig. S4i). Together, the results suggest that both enrichment of P-TEFb and relief from inhibition may be important for tissue-specific release of Pol II from promoter-proximal pausing.

### Repression involves exclusion of H3K27ac or induction of Polycomb-mediated H3K27me3

We next examined how active repression in non-expressing cells contributes to tissue-specific control of Pol II pausing by comparing the chromatin state at genes regulated by the Dl and Snail repressors (Additional file 1: Fig. S5a). Dl is converted to a repressor when its binding sites are flanked by AT-rich elements that recruit Capicua (Cic) and the co-repressor Groucho, resulting in long-range repression to delimit the ventral boundary of dorsal ectoderm-specific genes [53, 54]. As previously reported [14], the Polycomb-catalyzed mark H3K27me3 anti-correlates with expression of DV genes. Dl-repressed targets (dorsal ectoderm genes) accumulate H3K27me3 in both the neuroectoderm and mesoderm (Additional file 1: Fig. S5b). In contrast, whereas dorsal ectoderm genes are hypoacetylated in the mesoderm, H3K27ac is not completely blocked in the neuroectoderm (Additional file 1: Fig. S5b), despite similar levels of Dl at dorsal ectoderm enhancers in these two tissues as determined by CUT&Tag (Additional file 1: Fig. S5c). This indicates that Dl represses these genes by a mechanism that does not involve prevention of H3K27ac. Presumably, these enhancers are occupied by a different set of transcription factors and chromatin regulators in the neuroectoderm than in the mesoderm, leading to differences in the accumulation of H3K27ac.

In the mesoderm, Snail (Sna) works as a short-range repressor by recruiting the CtBP and Ebi co-repressors to shut down neuroectoderm-specific enhancers [16, 55, 56]. We found that the Sna repressor did not prevent occupancy of the Dl activator at neuroectoderm enhancers in the mesoderm (Additional file 1: Fig. S5c). Instead, prevention of H3K27ac in the mesoderm at neuroectoderm-expressed loci appears to be a major target of Sna-mediated repression (Fig. 2b, Additional file 1: Figs. S2i and S5b). This suggests that Sna quenches the Dl activator in the mesoderm by preventing CBP-mediated H3K27ac. However, Sna-targets did not accumulate H3K27me3 in the mesoderm (Additional file 1: Fig. S5b), consistent with the notion that Sna represses transcription by a different mechanism.

In line with previous findings [14, 16], the data show that whereas Dl-mediated repression is often accompanied by Polycomb-silencing and H3K27me3, repression by Sna involves prevention of H3K27ac without induction of H3K27me3. However, both mechanisms prevent release of paused Pol II into elongation (Fig. 1h).

### DV enhancers and promoters are temporally primed by pioneer factors for increased accessibility prior to induction of DV transcription

**Fig. 4:**
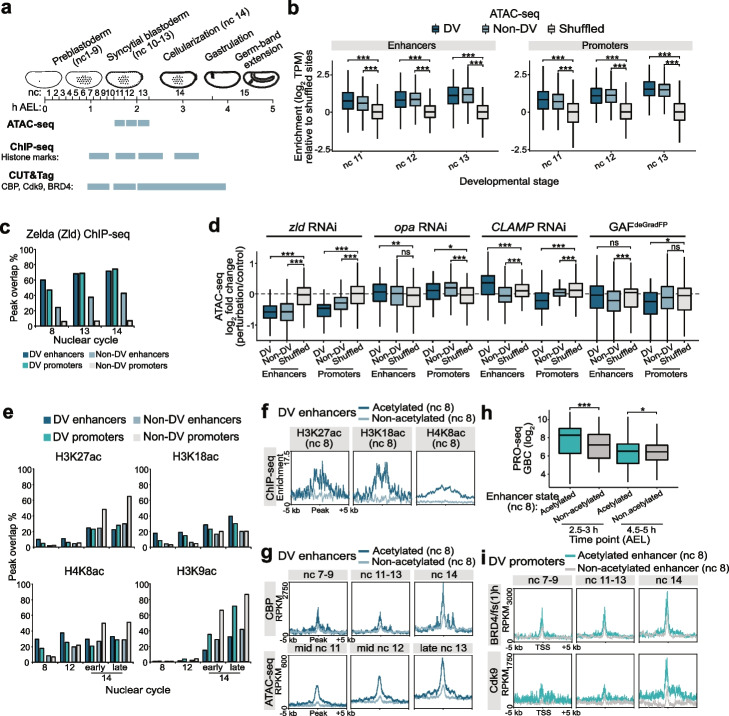
DV enhancers are primed by increased chromatin accessibility and CBP-mediated histone acetylation prior to the induction of DV transcription.** a** Schematic of the developmental stages profiled by ATAC-seq [36], ChIP-seq [57], and CUT&Tag (this study). **b** Boxplots of ATAC-seq enrichment (log_2_ TPM) at DV and non-DV enhancers and promoters, relative to shuffled genomic regions from wild-type embryos at nc 11, 12, and 13. Significant differences (Wilcoxon rank-sum test) are indicated by asterisks, * = *P* < 0.05, ** = *P* < 0.01, *** = *P* < 0.001. **c** Overlap (%) of DV and non-DV enhancers and promoters with Zld ChIP-seq peaks from nc 8, 13, and 14 wild-type embryos [58]. **d** Boxplots showing the log_2_ fold change (perturbation/control) in ATAC-seq signal at DV, non-DV, and shuffled enhancers and promoters after maternal RNAi depletion of *zld* and *opa* [59], *CLAMP* [60], and zygotic GAF^deGradFP^ [61]. *P*-values (Wilcoxon rank-sum test) show significant differences in accessibility compared to shuffled sites. **e** Overlap (%) of DV and non-DV enhancers and promoters with ChIP-seq peaks (nc 8, 12, 14 (early and late)) for the p300/CBP-mediated histone acetylation marks (H3K27ac, H3K18ac, and H4K8ac) and the non-p300/CBP mark H3K9ac [57]. **f–i** Metagene plots of (**f**) CBP-catalyzed histone marks from nc 8 ChIP-seq and **g** CBP CUT&Tag and ATAC-seq enrichment at DV enhancers acetylated or non-acetylated at nc 8. **h** Boxplots of 2.5–3 h and 4.5–5 h (AEL) PRO-seq gene body read counts (log_2_) for DV genes linked to enhancers acetylated or non-acetylated at nc 8. *P*-values are from the Wilcoxon rank-sum test. **i** Metagene plots of BRD4/fs(1)h and Cdk9 CUT&Tag signal from nc 7–9, 11–13, and 14 wild-type embryos at the promoters of DV genes linked to enhancers acetylated or non-acetylated at nc 8

We next aimed to complement our tissue-resolved map of the activity of DV enhancers and promoters by exploring the temporal dynamics of chromatin and transcriptional states during DV patterning (Fig. 4a). We plotted the chromatin accessibility at DV enhancers and promoters using previously reported ATAC-seq data from wild-type embryos through nuclear cycles 11–13, immediately preceding ZGA [36]. Since the Dl gradient response gradually appears between nuclear cycles (nc) 12–14, we expect chromatin accessibility to be largely uniform across cells in wild-type embryos during nc 11–13. We found that both DV and non-DV enhancers and promoters were significantly more accessible than shuffled sites representative of the genomic background prior to the initiation of DV gene transcription (Fig. 4b).

Consistent with the early priming of regulatory elements, the pioneer factor Zld, which has been shown to potentiate Dl activity at DV enhancers [62], is already highly enriched at DV enhancers and promoters in nc 8 embryos (Fig. 4c and Additional file 1: Fig. S6a) [58]. Alongside Zld, three factors with pioneer-like activities in the early embryo have been identified, Odd-paired (Opa) [59, 63], CLAMP [60], and GAGA-factor (GAF, also known as Trithorax-like, Trl) [61]. We found that Opa and CLAMP occupy both DV enhancers and promoters, whereas GAF favours DV promoters (Additional file 1: Fig. S6b). We analyzed published ATAC-seq data from embryos where each factor had been perturbed [59–61] (Fig. 4d). We observed a pronounced loss of accessibility at both DV and non-DV enhancers and promoters in *zld* RNAi embryos, a small change from *opa* RNAi, an unexpected slight increase in DV enhancer accessibility in *CLAMP* RNAi embryos and a small loss of accessibility at DV promoters upon GAF inactivation (Fig. 4d). This is consistent with earlier work demonstrating a function for Zld in expression and accessibility of DV genes [58, 62].

### CBP-mediated acetylation primes a subset of DV enhancers for strong induction of tissue-specific transcription

To investigate if the priming of chromatin accessibility at DV enhancers and promoters is accompanied by changes in histone modifications, we reanalyzed published spike-in normalized ChIP-seq data for a wide range of histone marks from nc 8, 12, 14a (early), and 14c (late) wild-type embryos [57]. As previously observed, the CBP-catalyzed marks H3K27ac, H3K18ac, and H4K8ac progressively accumulated at enhancers and promoters from nc 12 to nc 14c, with a subset of DV and non-DV enhancers already marked by histone acetylation at nc 8 (Fig. 4e and Additional file 1: Fig. S6c). By contrast, deposition of non-CBP-catalyzed H3K9ac, and methylation of H3K4 (H3K4me1/me3) occurred co-transcriptionally at nc 14 (Fig. 4e and Additional file 1: Fig. S6c). Interestingly, a greater proportion of DV than non-DV enhancers were marked by H3K27ac, H3K18ac, and H4K8ac prior to ZGA (Fig. 4e). We compared the overlap of enhancers with acetylation peaks over time and identified 48 DV enhancers already marked by a CBP-catalyzed acetylation at nc 8 (Fig. 4f and Additional file 1: Fig. S6d). Of these, 96% overlap Zld ChIP-seq peaks from the same stage, compared to 46% of the non-acetylated DV enhancers (Additional file 1: Fig. S6e) [57].

The deposition of histone acetylation at a subset of DV enhancers prior to ZGA suggests CBP is recruited to chromatin before DV transcription initiates. To test this, we performed CUT&Tag on hand-sorted nc 7–9, 11–13, and 14 embryos, which demonstrated that CBP was enriched at DV enhancers and promoters relative to shuffled genomic regions already at nc 7–9 (Additional file 1: Fig. S6f). The Zld-bound early acetylated DV enhancers were more enriched for CBP and had markedly higher accessibility than non-acetylated enhancers across the pre-ZGA nuclear cycles (Fig. 4g). Promoters linked to the early acetylated enhancers were also more enriched for histone acetylation than promoters linked to non-acetylated enhancers, had higher chromatin accessibility, and had stronger CBP enrichment (Additional file 1: Fig. S6g,h). To assess whether the early establishment of an active chromatin state influenced transcriptional activity, we compared the PRO-seq gene body signal for DV genes associated with early acetylated and non-acetylated enhancers at early (2.5–3 h) and late (4.5–5 h) stages of DV gene induction (Fig. 4h). PRO-seq revealed that DV genes with early formed active chromatin state established stronger tissue-specific transcription at the beginning of nc 14 (2.5–3 h AEL) (Fig. 4h). Thus, our data suggest that a subset of DV enhancers are primed by Zld for establishment of an active chromatin state defined by elevated chromatin accessibility, recruitment of CBP, and enrichment of CBP-catalyzed histone acetylations, and is associated with strong induction of tissue-specific transcription.

### Strong P-TEFb enrichment at DV promoters is not observed until gene expression is initiated

Since DV genes are paused but not expressed in naïve embryos, we examined when P-TEFb and BRD4/fs(1)h became associated with these genes. We performed CUT&Tag for CDK9 and BRD4/fs(1)h on nc 7–9, 11–13, and 14 embryos. We detected significant enrichment of BRD4/fs(1)h at DV enhancers and promoters, relative to shuffled genomic regions, already at nc 7–9 (Additional file 1: Fig. S6e). The promoters of DV genes associated with early acetylated enhancers also exhibited stronger enrichment of BRD4/fs(1)h than other DV promoters across the time course (Fig. 4i). Interestingly, although weak enrichment of CDK9 was observed at nc 7–9 and 11–13 at DV promoters linked to both early acetylated and not-acetylated enhancers, strong CDK9 recruitment occurred concomitantly with the induction of expression at nc 14, with promoters linked to early acetylated enhancers having the strongest occupancy (Fig. 4i).

Taken together, the data are consistent with a model where DV enhancers are temporally primed by the pioneer factor Zld leading to an active chromatin state and BRD4/fs(1)h recruitment prior to the induction of DV-responsive transcription. However, strong loading of P-TEFb to the promoter does not occur until nc 14, which may trigger the release of paused Pol II and induction of tissue-specific gene expression.

### Identification of DV cell clusters from single-cell expression data

**Fig. 5:**
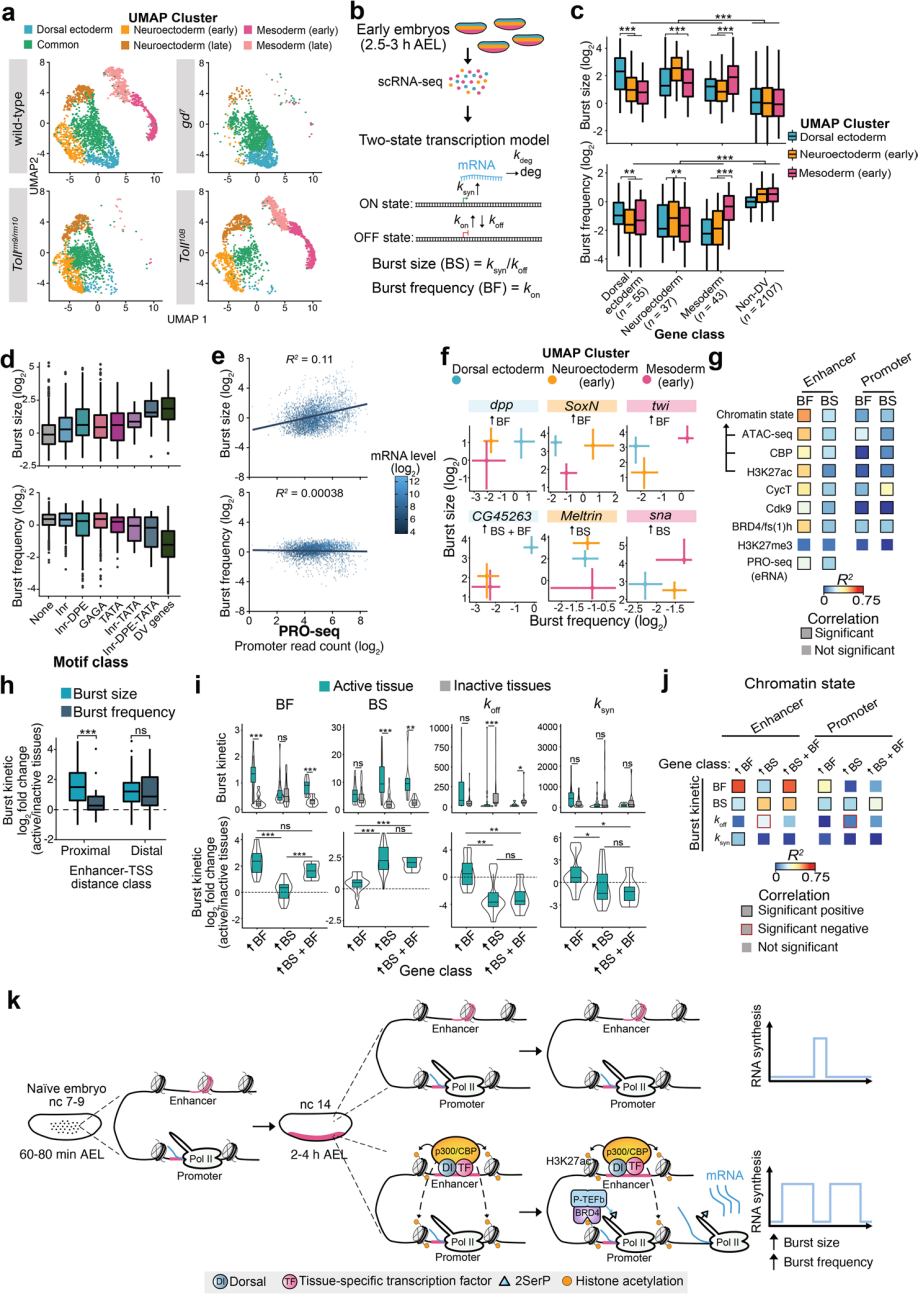
Transcriptional kinetics inferred from scRNA-seq data show that DV genes have a high burst size and are regulated in burst size or frequency.** a** UMAP clustering of single-cell RNA-seq (scRNA-seq) from DV-relevant clusters in wild-type and *Toll* mutant 2.5–3.5h embryos [9] based on the expression of DV genes identified by PRO-seq. **b** Schematic of the two-state transcriptional model used for transcriptome-wide inference of burst kinetics from scRNA-seq [65]. **c** Boxplots showing the burst size and frequency (log_2_) of DV genes classified by the tissue of expression in DV-relevant UMAP clusters from wild-type scRNA-seq. **d** Boxplots of the burst size and frequency of genes classified by the presence of de novo identified promoter motifs and compared to all DV genes. **e** Correlations between transcriptional kinetics and PRO-seq promoter read counts (log_2_). The mRNA level (log_2_ TPM) of genes is denoted. **f** Plots showing the transcriptional kinetics of individual DV genes (*dpp*, *CG45263*, *SoxN*, *Meltrin*, *twi*, and *sna*) across DV-relevant UMAP clusters. Error bars show the 95% confidence intervals. Genes with statistically significant increases in bursting kinetics in the cluster of expression relative to the OFF clusters are denoted. **g** Heatmap of the coefficient of determination (*R*^*2*^) between the tissue-specific enrichment of various genomic datasets at DV enhancers and promoters compared to burst frequency (BF) and size (BS) differences for enhancer-paired DV genes with significant changes in kinetics between DV clusters (*n* = 29). Comparisons with significant correlations are denoted. See Additional file 6: Table S7 for *R*^*2*^ and *P*-values. **h** Boxplots showing the fold change (log_2_) in transcriptional kinetics between the active tissue relative to the inactive tissues for genes regulated by proximal (≤ 700 bp from the TSS) and distal enhancers. Significant differences (Wilcoxon rank-sum test) are indicated by asterisks, *** = *P* < 0.001. **i** (Top) Boxplots of the inferred transcriptional kinetics for enhancer-paired DV genes partitioned into classes based on whether they have a significant kinetic change in burst frequency (*n* = 8), size (*n* = 16), or both (*n* = 5) between the active and inactive clusters (see also Additional file 1: Fig. S8h). (Bottom) For each class, the log_2_ fold change (active/inactive tissues) is plotted. Significant differences (Wilcoxon rank-sum test) are indicated by asterisks, * = *P* < 0.05, ** = *P* < 0.01, *** = *P* < 0.001.** j** Heatmap showing the coefficient of determination (*R*^*2*^) between the chromatin state change at DV enhancers and promoters compared to the kinetics inferred for each class. Comparisons with significant positive and negative correlations are denoted. **k** Schematic model of tissue-specific DV gene transcriptional activation

Quantitative studies have revealed that transcription is stochastic and occurs in bursts [64]. Our results show that the DV genes are regulated by pause release, but mediation of the release of paused Pol II to produce bursts of transcription is poorly understood. We analyzed single-cell RNA-seq (scRNA-seq) data from wild-type and *Toll* mutant 2.5–3.5 h (AEL) embryos [9] to link these processes. Clustering of single-cell expression profiles previously identified 15 clusters representing different cell identities in the early embryo (Additional file 1: Fig. S7a) [9]. We performed principal component analysis (PCA) using the DV genes identified by PRO-seq on cells from the ectoderm, neural, and mesoderm clusters, and used shared nearest neighbor (SNN) clustering on the first 10 principal components to assign 6 new clusters and visualized it with Uniform Manifold Approximation and Projection (UMAP) (Fig. 5a, Additional file 1: Fig. S7b, c and Additional file 5: Table S5). UMAPs from *gd*^*7*^, *Toll*^*rm9/rm10*^, and *Toll*^*10B*^ embryos revealed the absence of specific clusters in mutant embryos (Fig. 5a). This shows that the mutant embryos largely reflect the three presumptive germ layers, but that *Toll*^*10B*^ embryos consist of 49% mesoderm cells and 34% cells that resemble neuroectoderm (Additional file 1: Fig. S7d), further clarifying previous results [9]. The scRNA-seq profiles of *dpp*, *ind*, and *twi* in *Toll* mutant embryos are shown in Additional file 1: Fig. S7e.

### Transcriptome-wide inference of burst kinetics in different cell types reveals that DV genes have high burst size capacities and constrained burst frequencies

The scRNA-seq data from wild-type embryos was used to infer transcriptional burst kinetics based on a two-state model of transcription [65] (Fig 5b). The two-state model consists of four parameters that may accommodate different transcriptional kinetics. The rate of transition to the active state, *k*_on_; the rate of transition to the inactive state, *k*_off_; the rate of transcription in the active state, *k*_syn_; and the mRNA degradation rate, *k*_deg_. Here, we mainly characterized bursting by the burst frequency (*k*_on_; in units of mean mRNA degradation rate) and burst size (mean number of transcripts produced per active burst; *k*_syn_/*k*_off_). We modelled gene expression using bootstrapped maximum likelihood inferences to obtain estimates and confidence intervals on burst frequency and size [65], and for each cluster removed genes with no or low burst size and uncertain kinetic parameters (Additional file 1: Fig. S7f, g). Burst kinetics could be determined for a total of 3751 genes, including 135 DV genes, in at least 2 of 3 DV clusters (dorsal ectoderm, neuroectoderm (early) and mesoderm (early) (Fig. 5a, Additional file 5: Table S6), and the kinetic values inferred were highly concordant between two different wild-type lines (*PCNA:eGFP* and* w*^*1118*^, Additional file 1: Fig. S7h). The analysis revealed that DV genes, as well as AP patterning genes, have high burst sizes and low burst frequencies compared to non-DV genes, suggesting that they fire infrequently but produce many transcripts per burst (Fig 5c and Additional file 1: Fig. S8a). Since high burst size has previously been associated with the occurrence of certain core promoter motifs [65], we plotted the burst sizes and frequencies of all genes (*n*=2291) associated with no motifs, with individual motifs or with a combination of promoter motifs (Fig. 5d). This showed that genes associated with Inr, DPE, and TATA had a high burst size but low burst frequency. Since these motifs are overrepresented in the DV genes (Additional file 1: Fig. S1j-m), it may partly explain their high burst size capacity. We also plotted the burst size and frequency relative to the level of Pol II promoter-proximal pausing genome-wide (Fig. 5e). We noted a weak correlation between pausing and burst size, but not burst frequency. Pausing correlated better with burst size than burst frequency also for DV genes but the correlation was even weaker, likely due to the small sample size (Additional file 1: Fig. S8b). Thus, an enrichment of core promoter motifs may partly explain the high burst size of DV genes, whereas Pol II pausing has less influence.

### Differences in burst kinetics between cell types correlate with differences in enhancer chromatin state

By comparing the burst kinetics inferred in the dorsal ectoderm, neuroectoderm (early), and mesoderm (early) cell clusters, we were able to measure changes in DV gene burst sizes and frequencies between cells where these genes are active or inactive, but still detectable by scRNA-seq (Fig. 5c). Comparison between the clusters showed that both burst size and frequency were significantly higher for DV genes in the cluster of expression (Fig. 5c). To explore whether the relative contributions of burst size and frequency parameters vary between genes, we plotted the burst size and frequency with confidence intervals for individual DV genes in the three clusters (Fig. 5f, Additional file 5: Table S6). This revealed that DV genes have different dependencies on burst size and frequency changes during bursts. We found that of the 47 PRO-seq identified DV genes with a significant change in one or both kinetic values, 16 significantly changed in burst frequency (e.g., *dpp, SoxN and twi*), 25 increased in burst size (e.g., *Meltrin* and *sna*) and 6 genes changed in both burst size and frequency (e.g., *CG45263*) (Fig. 5f, Additional file 1: Fig. S8c-e). There were more dorsal ectoderm and neuroectoderm-specific genes that significantly increased in burst size than burst frequency whereas more mesoderm-specific genes changed in burst frequency (Additional file 1: Fig. S8d,e). To validate our scRNA-seq inferred burst kinetic values, we compared them to data for three genes derived from live imaging [66, 67], showing similar trends between the methods (Additional file 1: Fig. S8f). For example, live imaging shows that *sog* bursts more frequently in ventral regions of the neuroectoderm compared to dorsal regions of the neuroectoderm without changes to burst size. Similarly, *sog* burst frequency is higher in the neuroectoderm than in the adjacent dorsal ectoderm, whereas the change in burst size between these two cell types is small according to scRNA-seq (Additional file 1: Fig. S8f).

Since histone acetylation has been suggested to influence transcription by modulating burst frequency [65, 68], we sought to correlate tissue-specific differences in burst kinetics to our genomic datasets. For the enhancer-paired DV genes with a significant kinetic change (*n* = 29), we found that the combined tissue-specific chromatin state at enhancers was a good predictor of changes in burst frequency between tissues (*R*^*2*^ = 0.54), and correlated better with burst frequency changes than histone acetylation, CBP occupancy, or chromatin accessibility individually (Fig. 5g, Additional file 1: Fig. S8g and Additional file 6: Table S7). In contrast, the chromatin state at promoters was poor at predicting changes in burst frequency. Enhancer P-TEFb, BRD4/fs(1)h and eRNA transcription also correlated significantly with burst frequency, but were not as good predictors as the combined chromatin score (Fig. 5g). Differences in burst size between tissues could not be explained as well as burst frequency by the chromatin state, but a significant correlation of moderate strength was noted at enhancers (Fig. 5g and Additional file 1: Fig. S8g, *R*^*2*^ = 0.25). Interestingly, loading of CycT at promoters was the best predictor of burst size changes (*R*^*2*^ = 0.39), indicating that release from pausing may influence the burst size (Fig. 5g). We also explored if the enhancer-promoter distance influenced the modulation of burst size or frequency during activation. Interestingly, for DV enhancers located proximal (< 700 bp) to their target promoters (*n* = 22), bursts involved a significantly stronger shift in size than frequency, whereas genes regulated by distal enhancers (*n* = 115) shifted in both burst size and frequency upon activation (Fig. 5h).

To further explore DV gene bursts, we plotted the kinetics for genes partitioned into classes based on whether they significantly changed in burst size (*n* = 16 genes, 33 enhancers), burst frequency (*n* = 8 genes, 19 enhancers) or both (*n* = 5 genes, 6 enhancers) in the cell cluster of expression compared to the inactive tissues (Fig. 5i). Burst frequency (*k*_on_) and burst size (*k*_syn_/*k*_off_) can reliably be inferred from scRNA-data [65], but how well the individual *k*_syn_ and *k*_off_ parameters can be estimated is more uncertain. We observed that increases in burst size appear to occur from lower off rates (*k*_off_) and not from increases in the rate of transcription (*k*_syn_) in the tissue of activity (Fig. 5i). This is consistent with the tightly constrained initiation rates observed for gap genes along different positions in the early embryo by single-molecule fluorescent in situ hybridization (smFISH) [69]. Although there is uncertainty in the *k*_syn_ and *k*_off_ parameters in our data, the results indicate that genes with increased burst size may remain in the ON state for a longer duration when they are activated.

Examining the correlations between each parameter and the combined chromatin state suggests that, as expected, differences in enhancer chromatin state correlate well with burst frequency for genes that change significantly in burst frequency between active and inactive cells (*R*^2^ = 0.64, Fig. 5j). Interestingly, for genes changing in burst size, the enhancer chromatin state correlates well with burst size (*R*^*2*^ = 0.48) and promoter mean occupancy (*k*_on_/(*k*_on_+_*k*off_), *R*^*2*^ = 0.49), but not so well with burst frequency differences (*R*^2^ = 0.22) (Fig. 5j and Additional file 1: Fig. S8h, i). This suggests that while the enhancer chromatin state primarily influences burst frequency, it can also modulate transcriptional bursts through other parameters in a context-dependent manner.

Taken together, our transcriptome-wide inference of transcriptional bursting dynamics during DV patterning show that DV genes have the capacity for high burst size, but a lower burst frequency than non-DV genes, and that individual genes vary in their dependencies on changes in size and frequency kinetics during bursts. Combining the burst data with our comprehensive genome-wide epigenomic data reveals that the enhancer chromatin state strongly modulates burst frequency, and has less, albeit still significant, influence on burst size. The high burst size capacity of DV genes is encoded by core promoter motifs that mediate strong TFIID and Pol II recruitment, while tissue-specific P-TEFb activity ensures that bursts are only triggered in specific cells.

## Discussion

The establishment and maintenance of differential gene expression programs allows cells within multicellular organisms that contain genomes with identical DNA sequences to form distinct specialized tissues during embryogenesis. Yet, the interplay between chromatin state and transcription is not entirely understood. Here we have provided a comprehensive genome-wide assessment of chromatin state during *Drosophila* DV patterning, as measured by histone acetylation, chromatin accessibility, and CBP occupancy, and directly compared it to zygotic transcription and Pol II activity status. The use of homogenous DV tissue mutants invariant in chromatin state and transcription allowed us to dissect the interplay between the two. We note, however, that scRNA-seq revealed that *Toll*^*10B*^ mutant embryos are less homogenous than *Toll*^*rm9/rm10*^ and *gd*^*7*^ embryos, indicating that peak levels of Toll signaling are not obtained throughout these embryos. Still, and consistent with data from mammals [30], we find that the chromatin state at promoters is largely similar across tissues and cell types, but that enhancers are marked by tissue-specific chromatin accessibility, histone acetylation, and CBP occupancy.

This indicates that CBP fulfils distinct roles at enhancers and promoters. At enhancers, CBP is recruited and activated by dimerization induced by tissue-specific TFs to catalyze H3K27ac [41], which activates enhancers and stimulates target gene transcription. At promoters, CBP functions in the recruitment and establishment of a paused Pol II, possibly by interactions with the general transcription factor TFIIB [35]. These results suggest that detection of CBP at the promoter is not simply a result of looping of the promoter to CBP-bound enhancers, as CBP can be enriched at the promoters of DV genes in a tissue in which it is absent at the enhancer. Further work is needed to elucidate the mechanisms underlying the deployment of distinct CBP activities at promoters and enhancers.

Interestingly, our results show that Pol II pauses at the promoters of DV genes in a tissue-invariant manner, irrespective of future transcription activation. Pol II promoter pausing has previously been shown to be an important regulatory step in the transcription of developmental genes and has been suggested to prime developmental genes for subsequent activation [11]. Another function for Pol II pausing could be to promote synchronous gene activation across cells in a tissue [13], and to minimize expression variability between cells in a tissue [25]. This property is largely defined by core promoter elements, with paused and synchronous genes often having Initiator (Inr), downstream promoter element (DPE), and pause button (PB) sequences, whereas TATA-containing genes show higher variability in expression and are less paused [11, 25].

We find that pause release of Pol II is the critical regulatory checkpoint that dictates differential gene expression along the DV axis. Pausing is associated with the negative elongation factor (NELF) and DRB sensitivity inducing factor (DSIF, consisting of Spt4 and Spt5), and release of paused Pol II into productive elongation requires recruitment of the positive transcription elongation factor b (P-TEFb) kinase (reviewed in [52]). P-TEFb phosphorylates NELF, DSIF, and the C-terminal domain (CTD) of the Pol II largest subunit, allowing Pol II to escape from the pause site. Thus, control of P-TEFb recruitment and activity could be the key event in embryonic DV patterning. Indeed, we find that P-TEFb occupancy is spatially and temporally linked to DV gene activity. Interestingly, REL-family proteins such as NFκB regulate genes by targeting P-TEFb and transcription elongation in mammals (reviewed in [70]), suggesting that the REL-protein Dl may also specify dorsoventral cell fates primarily by promoting pause release. In addition to transcription factors such as Dl, enhancer chromatin state could also influence pause release. We have previously shown that increased histone acetylation leads to release from pausing at a subset of genes [71], so the correlation we find between H3K27ac and tissue-specific gene activity may promote transcription elongation. It will be interesting to further investigate how signals from the enhancer can modulate the activity of P-TEFb.

Once a gene is turned on, transcription is not continuous, but occurs in bursts. We found that compared to other genes, DV genes have a low burst frequency but a high burst size. Thus, many transcripts are produced per burst. This may result from an enrichment of core promoter motifs in DV genes that bind TFIID and promotes transcription reinitiation [72], and possibly from promoter-proximal pausing. However, pausing may represent an alternative OFF state that is not captured by a two-state model of transcription [73, 74]. The majority of DV genes have a higher burst size in their tissue of expression compared to non-expressing cells. The burst size is determined by the initiation rate and the off rate. Our transcriptome-wide analysis showed that the initiation rate (*k*_syn_) is less variable than the off rate (*k*_off_) for genes that increase their burst size in the tissue of expression. Burst size has also been shown to increase in response to Notch signaling [75, 76], primarily due to an increased burst duration. Although we cannot fully explain the increase in burst size in cells where DV genes are expressed, we note that the presence of P-TEFb at the promoter may play a role, as well as the chromatin state at enhancers. Another interesting finding is that enhancer-promoter distance appears to influence burst kinetics, with proximal enhancers modulating burst size, whereas distal enhancers influence both burst size and frequency. The difference in chromatin state at enhancers between cells has a large impact on burst frequency, consistent with previous findings [65, 68], and with a role for enhancers in modulating burst frequency [77].

Surprisingly, genes that are believed to be regulated in the same fashion have different bursting kinetics. Both *twi* and *sna* are activated by the Dl transcription factor in the mesoderm, but whereas *twi* has a higher burst frequency in the mesoderm compared to neuroectoderm and dorsal ectoderm, *sna* expression is driven by a higher burst size. Further, both *dpp* and *zen* are directly repressed by Dl in neuroectoderm and mesoderm, but whereas burst frequency is increased for *dpp*, the *zen* burst size increases in dorsal ectoderm. Regulation of promoter occupancy (*k*_on_/(*k*_on_ + *k*_off_)), i.e., the proportion of time the promoter is active, has been suggested to establish the expression domains of the *Drosophila* gap genes [69, 78], and two DV genes that respond to Dpp signaling [67]. Consistent with this, we find that promoter occupancy is higher in cells where DV genes are expressed compared to other cell types both for genes that change their burst size and for those that change in frequency (Additional file 1: Fig. S8h). However, we note that the kinetic parameters inferred in the framework of the two-state model may not be sufficient to fully explain the gene expression difference between cell types. Modulation of the window of time over which each cell transcribes the gene is a regulatory strategy that is independent of bursting, and important for *even-skipped* stripe 2 formation [79], which could also contribute to differential DV gene transcription.

## Conclusions

Overall, these results augment our current understanding of the interplay between the formation of chromatin state and transcription (Fig. 5k). The data suggest that tissue-specific DV enhancers and promoters are initially primed by increased accessibility across nuclei prior to ZGA by the action of the maternally supplied pioneer factor Zelda [62, 80]. Increased accessibility at enhancers is accompanied by CBP recruitment and histone acetylation, priming the genes for future activation. This provides amenability for recruitment of Dl, with occupancy occurring differentially across the DV axis of the embryo according to its nuclear concentration and enhancer-specific differences in motif composition that affect binding affinity. Dl leads to the tissue-specific recruitment of other TFs and co-regulators, including CBP and BRD4, leading to the adoption of distinct enhancer chromatin states spatially within the embryo. Concomitantly, promoters become accessible across tissues, permitting the recruitment of CBP and Pol II by unidentified factors. Pol II initiates transcription and pauses before the Dl gradient has formed and remains paused in all tissues. Recruitment and activation of P-TEFb, likely mediated by Dl and distinct enhancer chromatin states, leads to Pol II phosphorylation and possibly to phase-separated Pol II condensates [81], resulting in tissue-specific pause release and differential gene expression. The frequency of transcriptional bursts (*k*_on_) is to a large part determined by the enhancer chromatin state, whereas the burst size (*k*_syn_/*k*_off_) may also depend on P-TEFb. We speculate that P-TEFb activity is regulated and important for a high synthesis rate, leading to a high burst size in the tissue of expression.

## Methods

### Drosophila stock maintenance

Mutant *Drosophila melanogaster* embryos composed entirely of presumptive dorsal ectoderm, neuroectoderm, or mesoderm were obtained from the fly stocks *gd*^*7*^*/winscy hs-hid*, *Toll*^*rm9*^/*Toll*^*rm10*^/*TM6 e Tb Sb* and *Toll*^*10B*^*/TM3 e Sb Ser/OR60*, respectively. One-day-old larvae laid by *gd*^*7*^*/winscy hs-hid* were heat shocked for 1.5 h at 37°C for two consecutive days to eliminate *gd*^*7*^ heterozygous animals and presumptive dorsal ectoderm mutant embryos collected from the remaining *gd*^*7*^ homozygous flies. *Toll*^*rm9/rm10*^ trans-heterozygous females were separated from the stock and presumptive neuroectoderm embryos were collected from them. *Toll*^*10B*^*/TM3 e Sb* Ser and *Toll*^*10B*^*/OR60* heterozygote females that produce embryos composed of presumptive mesoderm were separated from the stock. Survival assays were performed to confirm embryonic lethality of *Toll* mutants. *yw; PCNA-eGFP*, a kind gift of Eric Wieschaus [36], and *w*^*1118*^ lines served as controls (wild-type) for ChIP-qPCR and RT-qPCR experiments. Flies in which the *dorsocross* (*doc*) locus E1 enhancer had been deleted (doc enh del^Δ/Δ^) using a CRISPR (clustered regularly interspaced short palindromic repeats)-Cas9 mediated deletion strategy and an intermediary line carrying flippase recognition target (FRT) sites flanking the intact E1 enhancer (doc enh^+/+^) that served as a control, were kind gifts from Mounia Lagha [7]. Flies carrying *UASp-CycT* and *Cdk9* transgenes were crossed with *w; alphaTub67C-GAL4::VP16* (Bloomington line 7062) and used for maternal P-TEFb overexpression (OE) (see “Overexpression of P-TEFb in early embryos” section).

Stocks were kept on potato mash-agar food and maintained at 25°C with a 12-h light/dark cycle. Embryos were collected on apple juice plates supplemented with fresh yeast and aged at 25°C for specific time ranges dependent on the specific experiment which is detailed in the relevant methods section. Plates containing embryos collected for the first 2 h each day were discarded to avoid contamination by older embryos withheld by females. Collected embryos were dechorionated in diluted bleach, rinsed thoroughly in embryo wash buffer (PBS, 0.1% Triton X-100) and processed further in a manner dependent on the specific experiment which is detailed in the relevant methods sections.

### Overexpression of P-TEFb in early embryos

Coding sequences for the P-TEFb subunits CycT and Cdk9 were PCR amplified and cloned into the pUAS-K10.attB vector [82] by restriction digest and ligation (see Additional file 7: Table S8 for primer sequences) to produce the plasmids “*pUASp-CycT*” and “*pUASp-Cdk9*.” CycT was cloned into pUAS-K10.attB using the *KpnI* and *XbaI* restriction sites whereas Cdk9 was cloned via *NotI* and *XbaI* sites. Plasmids were sequence verified, purified with the NucleoBond Xtra Midi kit (Machery-Nagel, Cat. 740410.50) and pUASp-CycT inserted into the attP2 landing site and pUASp-Cdk9 inserted into attP40 (FlyORF Injection Service). A double homozygous *UASp-Cdk9; UASp-CycT* stock was established and maternal overexpression achieved by crossing virgin females with *w; alphaTub67C-GAL4::VP16* (Bloomington line 7062) males. The resulting *UASp-Cdk9*/*alphaTub67C-GAL4::VP16 ; UASp-CycT/ +* females were collected, crossed with male siblings and used for embryo collection. For RNA in situ hybridization and ChIP-qPCR, P-TEFb OE, and wild-type (*w*^*1118*^) embryos were collected for 2 h and aged a further 2 h (2–4 h AEL) and for RNA extraction and RT-qPCR embryos were collected for 1 h and aged a further 1.5 h (1.5–2.5 h AEL).

### RNA in situ hybridization

RNA in situ hybridization was performed on wild-type (*w*^*1118*^), *gd*^*7*^, *Toll*^*rm9/rm10*^, and *Toll*^*10B*^ (2–4 h AEL) embryos using digoxigenin-labeled antisense RNA probes against *dpp*, *ind*, *twi*, and *shn*. Probes against *dpp*, *zen*, *ind*, *sog*, *twi*, and *sna* were used on wild-type (*w*^*1118*^) and P-TEFb OE (2–4 h AEL) embryos and a probe against *psq* was used on wild-type (*w*^*1118*^) (2–4 h AEL) embryos. RNA in situ hybridization was performed as previously described [83, 84]. Embryos were observed on a Leica DMLB 100T microscope and images taken on a Leica DMC2900 camera. We counted wild-type (wt) and P-TEFb OE (OE) embryos stained for *dpp* (wt *n* = 143, OE *n* = 206), *zen* (wt *n* = 119, OE *n* = 197), *ind* (wt *n* = 139, OE *n* = 178), *sog* (wt *n* = 155, OE *n* = 131), *twi* (wt *n* = 128, OE *n* = 86), and *sna* (wt *n* = 180, OE *n* = 112) manually for normal and ectopic signal and calculated the odds ratio alongside Fisher’s exact test to measure the significance of differences in the number of ectopically stained embryos between genotypes. Images of RNA in situ hybridization for *zen*, *ush*, *SoxN*, *Meltrin*, *twi*, *sna*, and *htl* in wild-type embryos were obtained from the BDGP database [85–87].

### Precision run-on sequencing (PRO-seq)

PRO-seq was performed on *Toll* mutant embryos collected for 0.5 h and aged for a further 2.5 h (2.5–3 h AEL) or 4.5 h (4.5–5 h AEL) and both PRO-seq and qPRO-seq were performed on naïve *yw; PCNA-eGFP* embryos collected for 20 min, aged for 1 h (60–80 min AEL) and hand-sorted according to the nuclear cycle observed by the eGFP signal with older embryos discarded. Collected embryos were dechorionated in dilute bleach and rinsed thoroughly in embryo wash buffer (PBS, 0.1% Triton X-100) before being flash-frozen in liquid nitrogen and stored at −80°C.

PRO-seq and qPRO-seq were performed as previously described [19, 20]. Briefly, embryos were resuspended in cold nuclear extraction buffer A (10 mM Tris-HCl pH 7.5, 300 mM sucrose, 10 mM NaCl, 3 mM CaCl_2_, 2 mM MgCl_2_, 0.1% Triton X, 0.5 mM DTT, protease inhibitor cocktail (Roche) and 4 µ/ml RNase inhibitor (SUPERaseIN, Ambion)), transferred to a dounce homogenizer and dounced with the loose pestle for 20 strokes. To remove large debris, the suspension was passed through mesh followed by douncing with a tight pestle for 10 strokes. Nuclei were pelleted at 700*g* for 10 min at 4°C and washed twice in buffer A and once in buffer D (10 mM Tris-HCl pH 8, 25% glycerol, 5mM MgAc_2_, 0.1 mM EDTA, 5 mM DTT). For PRO-seq, isolated nuclei corresponding to approximately 10 million cells, and for qPRO-seq from 1 million cells, were resuspended in buffer D and stored at −80°C. Nuclear run-on assays were performed in biological duplicates exactly as previously described [19, 20, 35]. PRO-seq and qPRO-seq libraries were sequenced (single-end 1 × 75 bp) on the Illumina NextSeq 550 platform at the BEA core facility, Karolinska Institutet, Stockholm.

### Assay for Transposase-Accessible Chromatin using sequencing (ATAC-seq)

ATAC-seq was performed on *Toll* mutant embryos collected for 0.5 h and aged accordingly to achieve three developmental time points: 2.5–3 h, 3.5–4 h, and 4.5–5 h AEL. For each time point, 10 embryos per replicate presenting the correct morphology for the developmental stage sought were immediately hand-sorted. Hand-sorted embryos were dechorionated in dilute bleach, rinsed thoroughly in embryo wash buffer (PBS, 0.1% Triton X-100) and crude nuclear extracts isolated by homogenizing the embryos using a motor pestle in ATAC lysis buffer (10 mM Tris pH 7.4, 10 mM NaCl, 3 mM MgCl_2_, and 0.1% IGEPAL CA-630) and centrifugating at 700*g* for 10 min. The nuclear pellet was resuspended in 22.5 μl of ATAC lysis buffer, 2.5 μl Tn5 (Tagment DNA Enzyme 1 (TDE1) (Illumina)), and 25 μl Tagment DNA Buffer (Illumina) and subjected to tagmentation at 37°C on a thermomixer at 1000 rpm. Transposition was blocked by the addition of 1% SDS and DNA purified with Agencourt AMPure XP beads (Beckman Coulter, A63881) according to the manufacturer’s instructions using a 2:1 ratio of beads to sample. Libraries were prepared as previously described [88]. Briefly, tagmented DNA was PCR amplified using 1x Phusion® High-Fidelity PCR Master Mix with GC Buffer (NEB) and 1.25 μM i5 and i7 PCR primers (Nextera® Index Kit (Illumina)) with the following PCR amplification conditions: 72°C for 5 min, followed by 10 cycles of 98°C for 10 s, 65°C for 1 min and 15 s, then 72°C for 1 min. Amplified libraries were purified with Agencourt AMPure XP beads with a 1.5:1 ratio of bead to sample volume. Libraries prepared from biological triplicates were sequenced paired-end (2 × 150 bp) on the Illumina NovaSeq platform at SciLifeLab, Stockholm.

### Chromatin immunoprecipitation sequencing (ChIP-seq) and ChIP-qPCR

ChIP-seq and ChIP-qPCR were performed on *Toll* mutant embryos collected for 2 h and aged for a further 2 h (2–4 h AEL) and ChIP-qPCR was also performed on *doc* enh del^Δ/Δ^ and *doc* enh^+/+^ (2–4 h AEL) control embryos and P-TEFb OE and wild-type (*w*^*1118*^) (2–4 h AEL) embryos. Formaldehyde crosslinking and chromatin preparation of embryos was performed as described previously [89]. Briefly, dechorionated embryos were crosslinked in a mixture of 2 ml fixation buffer (PBS, 0.5% Triton X-100) and 6 ml heptane supplemented with 100 μl of 37% formaldehyde (Sigma-Aldrich, F8775) for 15 min at room temperature with rotation. Fixation was quenched by the addition of PBS supplemented with 125 mM glycine and crosslinked embryos were washed 3 times in wash buffer (PBS, 0.5% Triton X-100), snap frozen in liquid nitrogen and stored at −80°C. For chromatin preparation, embryos were homogenized in a glass dounce homogenizer by 20 strokes with a tight pestle in A1 buffer (15 mM HEPES pH 7.6, 15 mM NaCl, 60 mM KCl, 4 mM MgCl_2_, 0.5 mM DTT, 0.5% Triton X-100 and protease inhibitor tablets (Roche)), centrifuged at 3500*g* for 5 min at 4°C and the supernatant discarded. The remaining nuclear pellet was resuspended in 200 μl of sonication buffer (15 mM HEPES pH 7.6, 140 mM NaCl, 1 mM EDTA pH 8.0, 0.5 mM EGTA pH 8.0, 0.1% sodium deoxycholate, 1% Triton X-100 and protease inhibitor tablets (Roche)) supplemented with 0.5% SDS and 0.2% n-lauroylsarcosine and sonicated using a Bioruptor (Diagenode) with high-power settings to obtain an average fragment size distribution of 200–500 bp, sonicated chromatin was centrifuged at 13,000 rpm for 10 min at 4°C and diluted 5-fold in sonication buffer to reduce the concentration of detergents.

For chromatin from *Toll* mutant embryos, immunoprecipitations (IPs) were performed with 10 μg rabbit anti-CBP (homemade, [17]), 2 μg rabbit anti-CycT and anti-Cdk9 (both kind gifts of Kazuko Hanyu-Nakamura [90]), and with 2 μg rabbit anti-H3K27ac (Abcam, ab4729) (also described in [9]) and 5 μg mouse anti-H3K27me3 (Abcam, ab6002) on chromatin from the *Toll*^*rm9/rm10*^ mutant. H3K27ac ChIP-seq data from *gd*^*7*^ and *Toll*^*10B*^ mutants used in this study were generated by [15]. IPs on chromatin from *doc* enh del^Δ/Δ^ and *doc* enh^+/+^ control embryos were performed with CycT, rabbit anti-BRD4/fs(1)h (long isoform) (a gift of Renato Paro, kindly provided by Nicola Iovino) [91]), CBP, H3K27ac, H3K27me3, and rabbit anti-H3 (Abcam, ab1791) antibodies. Chromatin from P-TEFb OE and wild-type (*w*^*1118*^) embryos was immunoprecipitated with anti-CycT. Chromatin was incubated with antibodies overnight at 4°C, and equal amounts of Protein A and G Dynabeads (Invitrogen), pre-blocked with BSA (1 mg/ml) were incubated with the samples for 4 h at 4°C. Chromatin corresponding to 10% of the amount in each IP was withdrawn to serve as an input for qPCR. Samples were subjected to 10 min washes with Wash A (10 mM Tris-HCl pH 8.0, 1 mM EDTA pH 8.0, 140 mM NaCl, 0.1% SDS, 0.1% sodium deoxycholate, and 1% Triton X-100), Wash B (Wash A adjusted to 500 mM NaCl), Wash C (10 mM Tris-HCl pH 8.0, 1 mM EDTA pH 8.0, 250 mM LiCl, 0.5% sodium deoxycholate, and 0.5% IGEPAL CA-630) and Tris-EDTA (TE) buffer. Beads were resuspended in 100 μl TE and treated with RNase A (20 μg/ml) at 55°C for 30 min before SDS (to 0.75%) and Tris-HCl (to 50mM) were added and crosslinks reversed at 65°C for overnight. Eluted ChIP DNA was treated with Proteinase K at 55°C for 2 h and purified with the ChIP DNA Clean & Concentrator kit (ZymoResearch, D5205).

ChIP-sequencing was performed using 2–5 ng of ChIP DNA from *Toll* mutant IPs with the anti-CBP antibody in biological duplicates. Libraries were prepared with the NEBNext® Ultra II DNA Library Prep Kit for Illumina (NEB, E7645L) and single-end (1 × 75 bp) sequenced on the Illumina NextSeq 550 platform at the BEA core facility, Karolinska Institutet, Stockholm.

ChIP DNA from IPs and inputs on *Toll* mutant chromatin (CycT), *doc* enh del^Δ/Δ^, and *doc* enh^+/+^ control chromatin (CycT, BRD4/fs(1)h, CBP, H3K27ac, H3K27me3 and H3) and P-TEFb OE and wild-type (*w*^*1118*^) chromatin (CycT) were analyzed by qPCR on a CFX96 Real-Time System (BioRad). qPCR reactions were carried out using 2 μl of ChIP DNA as a template with 300 nM primers and 5X HOT FIREPol® EvaGreen® qPCR Mix Plus (Solis BioDyne) in duplicate. All primers used in this study are listed in Additional file 7: Table S8. The percentage of input precipitated for each target was determined by comparing the average Cq to that of the input and the level of enrichment normalized to the signal at intergenic loci devoid of chromatin factors and histone modifications. For H3K27ac and H3K27me3, enrichment was further normalized to the occupancy of H3. Due to unexpected variations in the intergenic control signal between CycT IPs on P-TEFb OE and wild-type (*w*^*1118*^) chromatin, data were presented as the percent (%) input precipitated.

### CUT&Tag

CUT&Tag was performed on *Toll* mutant embryos collected for 2 h and aged for a further 2 h (2–4 h AEL) and *yw; PCNA-eGFP* embryos collected for 20 min and aged for 1 h (60–80 min AEL, nc 7–9), 30 min and aged for 1.5 h (1.5–2 h AEL, nc 11–13) and 2 h and aged for 2 h (2–4 h AEL, nc 14). Hand-sorting was performed to discard older embryos according to the nuclear cycle observed from the eGFP signal. Collected embryos were dechorionated, rinsed in embryo wash buffer (PBS, 0.1% Triton X-100) and crude nuclear extracts prepared using a glass douncer and loose pestle in Nuclear Extraction buffer (20 mM HEPES pH 7.9, 10 mM KCl, 0.5 mM spermidine, 0.1% Triton X-100, 20% glycerol with protease inhibitor cocktail (Roche)) [92] and centrifuged at 700*g* for 10 min at 4°C. The nuclear pellets were resuspended in Nuclear Extraction buffer. Nuclei corresponding to 50 embryos per reaction (2–4 h AEL), 100 embryos (1.5–2 h AEL), or 200 embryos (60–80 min AEL) were incubated with 30 μl of BioMag®Plus Concanavalin A beads (Polysciences) (prepared in Binding buffer (20 mM HEPES pH 7.5, 10 mM KCl, 1mM CaCl_2_, and 1 mM MnCl_2_)) on a nutator for 10 min at 4°C. Nuclei-bead complexes were resuspended in 100 μl antibody buffer (20 mM HEPES pH 7.5, 150 mM NaCl, 0.5 mM spermidine, 0.05% digitonin, 2 mM EDTA pH 8.0, and 0.1% BSA supplemented with protease inhibitor cocktail (Roche)). Antibodies were added and samples were incubated overnight at 4°C. For *Toll* mutant CUT&Tag, 1 μl of rabbit anti-BRD4/fs(1)h, rabbit anti-CycT, rabbit anti-Cdk9, rabbit anti-Rpb3 (a kind gift of John Lis), rabbit anti-RNA Polymerase II CTD repeat YSPTSPS (phospho-serine 5) (5SerP) (Abcam, ab5131), and guinea pig anti-Dorsal (a kind gift of Christos Samakovlis) were used. For *yw; PCNA-eGFP* CUT&Tag, 1 μl of rabbit anti-BRD4/fs(1)h, rabbit anti-Cdk9, and rabbit anti-CBP were used. Following overnight incubation, the experimental procedure was followed as previously described [93] using Donkey anti-Rabbit IgG (H+L) Alexa Fluor 568 (Invitrogen, A10042) and Rabbit anti-Guinea Pig IgG (Dako, P0141) secondary antibodies and purified pA-Tn5 (Protein Science Facility, KI, Stockholm). Tagmented DNA was PCR amplified using custom i5 and i7 PCR primers and Phusion® High-Fidelity PCR Master Mix with GC Buffer (NEB). PCR conditions were as follows: 72°C for 5 min, 98°C for 30 s, followed by thermocycling (98°C for 10 s and 63°C for 10 s) for 13 cycles and final extension at 72°C for 1 min. Amplified libraries were purified using Agencourt AMPure XP beads (Beckman Coulter) (1.1:1 bead to sample volume ratio). Libraries were paired-end (2 × 37 bp) sequenced on an Illumina NextSeq 550 platform at the BEA core facility, Karolinska Institutet, Stockholm. The low read counts obtained from sequencing for the CUT&Tag samples *gd*^*7*^ 2-4h CUT&Tag CycT Replicate 2, *gd*^*7*^ 2-4h CUT&Tag BRD4 Replicate 2, and *Toll*^*10B*^ 2-4h CUT&Tag Dl Replicate 2 indicated these reactions had failed so they were excluded from subsequent analysis.

### RNA extraction and RT-qPCR

Total RNA was extracted from *doc* enh del^Δ/Δ^ and *doc* enh^+/+^ (2–4 h AEL) and P-TEFb OE and wild-type (*w*^*1118*^) (1.5–2.5 h AEL) embryos. Dechorionated embryos were homogenized in cold PBS with a plastic pestle and RNA extracted using TRIzol LS (Invitrogen). Total RNA was purified and concentrated using the RNeasy MinElute Cleanup kit (Qiagen) according to the manufacturer’s instructions. Purified RNA (1.5 μg) was treated with DNase I (Sigma-Aldrich) to eliminate contaminating genomic DNA and converted to cDNA with the High-Capacity RNA-to-cDNA kit (Thermo Fisher Scientific) according to the manufacturer’s instructions. RT-qPCR was performed on a CFX96 Real-Time System (Biorad) using 2 μl of cDNA as template with 300 nM primers and 5X HOT FIREPol® EvaGreen® qPCR Mix Plus (Solis BioDyne) in duplicate. The delta-delta Ct method was used to quantify mRNA levels relative to RpL32 (RNA from *doc* enh del^Δ/Δ^ and *doc* enh^+/+^ embryos) and 28S rRNA (RNA from P-TEFb OE and wild-type (*w*^*1118*^)). All primers used in this study are listed in Additional file 7: Table S8.

### PRO-seq data analysis

PRO-seq and qPRO-seq reads were mapped to the *Drosophila melanogaster* (dm6) genome assembly using Bowtie2 (v.2.3.5) with the default program parameters [94]. Library mapping statistics are listed in Additional file 7: Table S9. Strand separated RPKM normalized (bigwig) coverage tracks from individual replicates were generated using the deepTools (v.3.5.1) package “bamCoverage” using the default parameters (binSize = 2 (bases), normalizeUsing = RPKM) [95]. Strand separated files of the mean RPKM signal from both replicates were produced by first merging the read alignment files produced by Bowtie2 from each replicate using the SAMtools package “samtools merge” and then bigwig files produced by “bamCoverage” (deepTools). To allow for simultaneous genome browser visualization of the signal from the pause site and gene body at genes of interest, the bin size was extended to 10 bp when producing the merged bigwig files.

To identity differentially expressed DV-regulated genes, the gene body read count (GBC, from the coding DNA sequence (CDS) to avoid counting intronic eRNAs) for annotated transcript were extracted with featureCounts [96], and normalized using DEseq2 [97]. Genes with < 10 reads mapping to the gene body were removed from the analysis. Principal component analysis (PCA) was performed on the normalized gene body read counts to ensure that the majority of variation between samples could be explained by the difference in *Toll* mutant genotype and developmental age. A subset of principal components (PCs) that separated the samples in the PC space were identified and three latent linear vectors, one for each *Toll* mutant, were constructed. The vectors pass through origo and the mean value of the *Toll* mutant sample PC scores with the positive direction towards the mean value of the *Toll* mutant. Latent vector scores were calculated for genes in each Toll mutant and further normalized to *z*-scores by removing the mean and dividing by the standard deviation from all regions. To test the validity of using a latent vector approach to identify differentially expressed genes and select a suitable cutoff, receiver operating characteristic (ROC) curve analysis was performed using DV genes previously identified from microarray data and validated experimentally by in situ hybridization [98] as a positive gene set. Based on the ROC analysis, genes with a latent vector-derived *z*-score of ≥ 3 were classified as DV regulated (*n* = 195) and assigned to the groups of dorsal ectoderm (*n* = 81), neuroectoderm (*n* = 56), and mesoderm (*n* = 58) based on the *Toll* mutant of transcriptional upregulation.

To examine Pol II promoter-proximal pausing, the promoter read counts (PC, defined as 50 bp upstream of the TSS to 100 bp downstream of the TSS) were extracted with featureCounts [96]. PCA was again performed on the normalized promoter read counts from DEseq2 [97]. The pausing index (PI), which is a ratio describing the magnitude of pausing, was calculated by dividing the normalized counts for the promoter by the sum of the promoter and gene body. For genes with multiple isoforms, the transcript with the highest sequence length-normalized GBC was selected. Between mutant statistical comparisons of gene body expression and PI for the same gene classes were performed with the Wilcoxon signed-rank test, whereas comparisons between separate gene classes used the Wilcoxon rank-sum test. A list of anterior-posterior (AP) regulated genes (*n* = 31) from Saunders A, Core LJ, Sutcliffe C, Lis JT and Ashe HL [99], and non-DV genes (*n* = 4741) were used as comparative data sets. The PRO-seq-derived gene body expression and PI of zygotic genes expressed at nc 7–9 (*n* = 20), nc 9–10 (*n* = 63), syncytial blastoderm (*n* = 946), and cellular blastoderm (*n* = 3540) stages of *Drosophila* embryogenesis were obtained from Kwasnieski JC, Orr-Weaver TL and Bartel DP [23] and used to validate the developmental staging of naïve embryo qPRO- and PRO-seq. See Additional file 2: Table S1 for the list of DV-regulated genes and *Toll* mutant PRO-seq PC and GBC data.

### Promoter motif analysis

We scanned DV promoters for putative core promoter elements from the CORE database and compared the proportion of promoters with motifs between DV promoters and all promoters in the database [27]. To de novo identify promoter motifs, we scanned DV-regulated promoters for ungapped enriched motifs using the Multiple EM for Motif Elicitation (MEME) tool from the MEME suite (https://meme-suite.org/meme). Long enriched motifs were identified with a threshold of 31 nt (-minw 31). For the other motifs, we required that the length to be ≤ 30 nt (-maxw 30). Motifs with an e-value less than 0.005 were kept for further analysis. The motifs were compared, using TOMTOM in the MEME-suite, against the motifs in the JASPAR Insects CORE redundant TF motifs database (version 2020). All of the de novo identified motifs (*P* < 0.005) were renamed based on matches to known motifs from the JASPAR database. Motifs that fitted the Inr and the DPE were assigned as Inr or Inr and DPE. The MEME suite tool FIMO was used to search DV, AP, and all other promoters for occurrences of the identified motifs. Log_2_ odds ratios were measured for the different motifs. See Additional file 3: Table S3 for the de novo identified motifs at DV promoters.

### ChIP-seq, ATAC-seq, and CUT&Tag data analysis

ChIP-seq, ATAC-seq, and CUT&Tag reads were mapped to the *Drosophila melanogaster* (dm6) genome assembly using Bowtie2 (v.2.3.5) with the default program parameters [94]. Library mapping statistics are listed in Additional file 7: Table S9. RPKM normalized (bigwig) coverage tracks from individual replicates were generated using the deepTools (v.3.5.1) package “bamCoverage” using the default parameters (binSize = 1 (bases), normalizeUsing = RPKM) [95]. The mean RPKM signal from both replicates were produced by first merging the read alignment files produced by Bowtie2 from each replicate using the SAMtools package “samtools merge” and then bigwig files produced by “bamCoverage” (deepTools). Peaks were called for *Toll* mutant ATAC-seq as well as CBP and H3K27ac [9, 14, 15] ChIP-seq using the Genrich peak caller (version 0.6) (https://github.com/jsh58/Genrich#contact) with the default program parameters.

To identify tissue-specific DV-regulated enhancers de novo from epigenomic data, the enrichment of *Toll* mutant CBP ChIP-seq, H3K27ac ChIP-seq [9, 14, 15], and ATAC-seq were profiled at peaks called for CBP (not overlapping the TSS to avoid promoter elements). Read counts were extracted with featureCounts [96] and normalized using DEseq2 [97]. Regions with < 10 reads were discarded and PCA was performed on the normalized read counts. A subset of principal components (PCs) that separated the samples in the PC space were identified (PCs 1 to 3 for ATAC-seq and 1 to 2 for CBP and H3K27ac ChIP-seq) and latent vector scores were calculated and normalized to *z*-scores as described for the PRO-seq data. Only regions with *z*-scores in the top 5% were considered potential enhancers. Combined tissue-specific chromatin state scores were calculated for each region by summing the *z*-scores for CBP and H3K27ac ChIP-seq and ATAC-seq from each *Toll* mutant. When assigning putative enhancers to DV-regulated genes identified from *Toll* mutant PRO-seq, a requirement was that they resided in the same topologically associated domain (TAD) (domain boundaries are from Hi-C data in 3–4 h AEL embryos [9, 44]). This approach identified 176 genomic regions as tissue-specific DV-regulated enhancers that were assigned to the groups of dorsal ectoderm (*n* = 72), neuroectoderm (*n* = 51), and mesoderm (*n* = 53) based on the *Toll* mutant of transcriptional upregulation of the paired DV-regulated genes.

To functionally validate the activity of the identified tissue-specific DV enhancers, we lifted annotation terms (*n* = 31) associated with the in vivo activity of 7793 enhancer reporter lines driven by non-coding genomic fragments in stage 4 to 6 *Drosophila* embryos [34]. We then measured the enrichment of annotation terms for reporter lines driven by fragments overlapping DV enhancers and compared to those overlapping all other annotated CBP peaks. Only terms with *P*-values < 0.005 (Fisher’s exact test) in at least one of the *Toll* mutant enhancers were kept. ROC curve analysis was performed to assess the quality of the enhancer identification strategy and compare the predictive accuracy of the individual and combined data. Non-DV assigned CBP peaks (referred to as non-DV enhancers, *n* = 9383) represented the negative set and the assigned tissue-specific enhancers that overlapped non-coding genomic fragments with DV-regulated activity in enhancer reporter assays [34] were used as the positive set.

The latent vector modelling approach was also applied to measure tissue-specific scores from *Toll* mutant CBP and H3K27ac ChIP-seq, and ATAC-seq data at promoters. Read counts were extracted from all promoter regions with featureCount [96], normalized using DEseq2 [97] and latent vector scores determined. Scores were obtained for promoters associated with the 195 PRO-seq identified differentially expressed DV genes, 107 of which could be paired to at least one tissue-specific DV enhancer (41 for dorsal ectoderm, 29 for neuroectoderm, and 37 for mesoderm). The remaining promoters were denoted as non-DV (*n* = 4865). The epigenomic data scores for DV and non-DV enhancers and promoters are listed in Additional file 4: Table S4.

Latent vector scores at DV and non-DV enhancers and promoters were also determined for the CycT, Cdk9, and BRD4/fs(1)h *Toll* mutant CUT&Tag data. Read counts for the CBP, Cdk9, and BRD4/fs(1)h CUT&Tag data from nc 7–9, 11–13, and 14 wild-type embryos were extracted with featureCount [96], normalized as log_2_ transcripts per million (TPM) and examined at DV, non-DV, and shuffled (obtained using BEDTools “shuffle” [100]) enhancers and promoters.

To quantify the temporal dynamics of chromatin accessibility at DV enhancers, the ATAC-seq signal from 3, 4, and 5 h AEL *Toll* mutants was counted using the deepTools “BigWigSummary” tool [95] and the log_2_ fold change in accessibility at 4 h and 5 h relative to 3 h measured. Accessibility changes were specifically measured for tissue-specific enhancers in the mutant of target gene expression. Enhancers were classified depending on whether they gained (log_2_ fold change (FC) ≥ 0.5), lost (log_2_ FC ≤ −0.5) or maintained stable chromatin accessibility (ATAC-seq) in 4 h and 5 h AEL embryos relative to 3 h. For dorsal ectoderm enhancers in *gd*^*7*^ embryos, at 4 h, 23 enhancers gained, 4 lost, and 46 had stable accessibility and at 5 h, 34 enhancers gained, 11 lost, and 28 had stable accessibility. For neuroectoderm enhancers in *Toll*^*rm9/rm10*^ embryos, at 4 h, 25 enhancers gained, 3 lost, and 23 had stable accessibility and at 5 h, 29 enhancers gained, 5 lost, and 17 had stable accessibility. For mesoderm enhancers in *Toll*^*10B*^ embryos, at 4 h, 22 enhancers gained, 8 lost, and 23 had stable accessibility and at 5 h, 25 enhancers gained, 13 lost, and 15 had stable accessibility. The PRO-seq gene body expression (log_2_ normalized read count) levels from early (2.5–3 h) and late (4.5–5 h) *Toll* mutants was examined for DV genes paired to gained (*n* = 57), lost (*n* = 24), and stable (*n* = 39) enhancers at 5 h AEL.

### Analysis of previously published datasets

In addition to the datasets generated in this study, we reanalyzed the following published datasets: ChIP-seq for H3K27ac and H3K27me3 from *gd*^*7*^ and *Toll*^*10B*^, and Sna and GAF from wild-type (Oregon-R) (2–4 h AEL) embryos (GEO: GSE68983) [14, 15]; ChIP-seq for H3K27ac and H3K27me3 from *Toll*^*rm9/rm10*^ (2–4 h AEL) embryos (ArrayExpress: E-MTAB-9303) and scRNA-seq from wild-type (*PCNA-eGFP* and *w*^*1118*^) and *Toll* mutant (2.5-3.5 h AEL) embryos (ArrayExpress: E-MTAB-9304) [9]; ChIP-nexus for Dl from wild-type (Oregon-R) (2–4 h AEL) embryos (GEO: GSE55306) [33]; ChIP-seq for Zld from nc 8, nc 13 and nc 14 wild-type embryos (GEO: GSE30757) [58]; ChIP-seq for Zld from wild-type (2–3 h AEL) embryos (GEO: GSE65441) [62]; ChIP-seq for opa from wild-type (ZH-86Fb) nc 14 (4 h AEL) and ATAC-seq from nc 14 wild-type (ZH-86Fb), *zld* and *opa* maternal RNAi embryos (GEO: GSE140722) [59]; ChIP-seq for CLAMP from wild-type (MTD-Gal4, Bloomington line 31777) (2-4 h AEL) embryos (GEO: GSE152598) and ATAC-seq from wild-type (MTD-Gal4, Bloomington line 31777) and *CLAMP* maternal RNAi (2–4 h AEL) embryos (GEO: GSE152596) [60]; ATAC-seq from control (His2AV-RFP; sfGFP-GAF) and GAF^deGradFP^ (His2Av-RFP/nos-degradFP; sfGFP-GAF) (2–2.5 h AEL) embryos (GEO: GSE152771) [61]; ChIP-seq for H3K27ac, H3K18ac, H4K8ac, H3K9ac, H3K4me1, and H3K4me3 ChIP-seq from wild-type (Oregon-R) (nc 8, nc 12, and nc 14 (early and late)) embryos (GEO: GSE58935) [57]; and ATAC-seq from wild-type embryos nc 11–13 (GEO: GSE83851) [36].

Reads for the publicly available data were mapped to the *Drosophila melanogaster* (dm6) genome assembly using Bowtie2 (v.2.3.5) with the default program parameters [94]. RPKM normalized (bigwig) coverage tracks from individual replicates were generated using the deepTools (v.3.5.1) package “bamCoverage” using the default parameters (binSize = 1 (bases), normalizeUsing = RPKM) [95]. For ATAC-seq data from wild-type embryos [36], bigwig files of the mean signal for replicates and the mean signal across each nuclear cycle were produced using the deepTools (v.3.5.1) package “bigwigCompare” using the default parameters. For ChIP-seq data for various histone modifications [57] and ATAC-seq data for from various pioneer factor perturbations [59–61], we used processed data sets generated in the original publications.

### Examining publicly available data at DV enhancer and promoters

Read counts for ATAC-seq data from nc 11–13 wild-type embryos [36] were extracted with featureCount [96], normalized as log_2_ transcripts per million (TPM), and examined at DV, non-DV, and shuffled (obtained using BEDTools “shuffle” [100]) enhancers and promoters.

BEDTools intersect [100] was used to examine the overlap between DV and non-DV enhancers and promoters with ChIP-seq peaks called for H3K27ac, H3K18ac, H4K8ac, and H3K9ac (nc 8, nc 12, and nc 14 (early and late)) [57] and Zld (nc 8, nc 13, and nc 14) [58] from wild-type embryos. This analysis identified a subset of DV enhancers (*n* = 48) that overlapped a peak called for at least one CBP-catalyzed histone acetylation (H3K27ac, H3K18ac, or H4K8ac) from nc 8. For measuring overlaps, promoter regions were defined as (TSS ± 750 bp). To preserve the original spike-in normalizations used for the ChIP-seq data from histone marks across early nuclear cycles [57], we used the coordinates for peaks called in the original paper using the dm3 *Drosophila* reference genome. We lifted peaks for DV and non-DV enhancers and promoters from dm6 to dm3 using the UCSC LiftOver tool. For Zld ChIP-seq across early nuclear cycles [58], we also used the peaks called in the original paper from the dm3 reference genome.

To quantify changes in ATAC-seq signal at DV enhancers and promoters from publicly available data for pioneer factor perturbations [59–61], the signal at DV, non-DV, and shuffled enhancers and promoters (TSS ± 500 bp) (obtained using BEDTools “shuffle” [100]) was counted using the deepTools “BigWigSummary” tool [95] and the log_2_ fold change (perturbation/control) in ATAC-seq signal measured. Boxplots were produced in R using the ggplot2 package and significant differences in the change in accessibility measured with the Wilcoxon rank-sum test.

### Uniform Manifold Approximation and Projection (UMAP) clustering of scRNA-seq data

We selected the cells in scRNA-seq from wild-type (*PCNA-eGFP*) embryos that had been originally assigned to DV-relevant clusters (ectoderm1, ectoderm2, ectoderm3, neural1, neural2, mesoderm1, and mesoderm2, *n* = 2787) based on clustering using the shared nearest neighbor (SNN) approach from the Seurat package (version 4.1.0) [9, 101, 102]. From the scRNA-seq data in the selected cells, a new principal component space was constructed using only the 195 DV genes identified in PRO-seq as features to separate the cells. SNN clustering was performed on the first 10 PCs with a clustering resolution of 0.3. Identified clusters were annotated based on the expression levels of PRO-seq identified DV genes. Based on the expression of DV marker genes, the derived clusters were named Dorsal ectoderm (*dpp, Doc1* and *ush* marker genes, *n* = 1396), early (*ind*, *sog* and *brk* marker genes, *n* = 1392), and late (older neural cells, *scrt*, *ase* and *nerfin-1* marker genes, *n* = 2367) Neuroectoderm, early (*twi and sna* marker genes, *n* = 2333) and late (older mesoderm or myoblasts, *Mef2*, *meso18E*, *sns* and *sing* marker genes, *n* = 1448) Mesoderm and a common cluster of cells (*n* = 851) that could not be separated according to the expression of the DV genes (Additional file 5: Table S5). Average expression levels within each cluster were obtained for 160 of the 195 DV-regulated genes, 26 of the 31 AP regulated genes, and 1819 non-DV genes (Additional file 5: Table S5). Uniform Manifold Approximation and Projections (UMAPs) were constructed using the default settings to visualize the scRNA-seq data.

### Inference of transcriptional bursting kinetics from scRNA-seq data

To infer transcriptional bursting kinetics, scRNA-seq UMI count matrices from the two wild-type samples (*PCNA:eGFP* and *w*^*1118*^) were first subsetted per cluster. For the dorsal ectoderm, neuroectoderm (early), and mesoderm (early) clusters, maximum likelihood kinetics inference was attempted for all detected genes according to the model implemented by Larsson AJM, Johnsson P, Hagemann-Jensen M, Hartmanis L, Faridani OR, Reinius B, Segerstolpe A, Rivera CM, Ren B, and Sandberg R [65]. Additionally, pseudorandom bootstraps of the input data before maximum likelihood inference in 100 iterations were performed. Through the bootstrapped inference, empirical confidence intervals were derived. Next, for each cluster, we filtered away low-power inferences outside of the parameter space by sorting the inferred burst size values into two distributions based on a mixture of two normal distribution curves using the normalMixEM tool from the mixtools package in R (version 1.2.0) (https://cran.r-project.org/web/packages/mixtools/vignettes/mixtools.pdf) and the values in the higher distribution were kept. Genes with noisy confidence inferences (i.e., a broad confidence interval (CI)) were discarded (For *k*_on_: log_10_(CI *k*_on_) < 1.3 + 0.8 log_10_(*k*_on_) and for *k*_bs_: log_10_(CI *k*_bs_) < 1.0 + 0.8 log_10_(*k*_bs_). Pearson correlations of the bursting kinetics for the DV clusters between the two wild-type samples were measured to control for reproducibility. Kinetics were obtained for 2232 genes in all three clusters and 1519 genes in two of the three clusters, including 135 of the 195 DV genes identified by PRO-seq (Additional file 5: Table S6). To facilitate direct comparison of live imaging- and scRNA-seq-derived burst kinetics, we also inferred kinetic values for *hindsight* (*hnt*) in cells annotated as amnioserosa by [9]. Changes in kinetics between clusters for genes were considered significant if the confidence intervals did not overlap. The 47 DV genes with a significant change in burst frequency (*n* = 16), size (*n* = 25), or both (*n* = 6) between the clusters, included 29 with an enhancer identified by epigenome profiling of chromatin state. The 29 enhancer-paired DV genes were separated into kinetic classes based on whether they changed significantly in burst frequency (*n* = 8), burst size (*n* = 16), or both (*n* = 5). Further parameterizations of transcriptional bursts: promoter mean occupancy ((*k*_on_/(*k*_on_+*k*_off_)); switching correlation time (1/(*k*_on_+*k*_off_)); and mean transcript synthesis rate ((*k*_syn_ x *k*_on_)/(*k*_on_ + *k*_off_)) were inferred to further characterize DV gene transcriptional activity [69]. Pearson correlations, coefficient of determination (*R*^2^), and *P*-values were measured from comparisons of burst kinetics against the tissue-specific scores of various *Toll* mutant epigenomic data at DV enhancers and promoters (ATAC-seq, CBP and H3K27ac ChIP-seq, CycT, Cdk9 and BRD4/fs(1)h CUT&Tag, PRO-seq) for the 29 enhancer-paired DV genes with a significant kinetic change (Additional file 6: Table S7).

### Statistical analysis

The statistical tests applied in this study are denoted in the relevant methods sections and figure legends. Asterisks were used to denote statistical tests that gave significant differences, * = *P* < 0.05, ** = *P* < 0.01, *** = *P* < 0.001. All *P*-values are provided in Additional file 6: Table S10, except for those from Fig. 5g, j and S8i which are listed in Table S7.

### Supplementary Information

**Additional file 1:** **Figure S1.** PRO-seq identifies DV regulated genes with promoter-proximal paused Pol II that persists across tissue types and developmental stages. **Figure S2.** Characterization of tissue-specific DV enhancers identified by epigenomic profiling of chromatin state. **Figure S3.** Temporal dynamics of chromatin accessibility and genome organization at DV genes [104]. **Figure S4.** Tissue-specific P-TEFb and BRD4/fs(1)h recruitment to DV genes. **Figure S5.** Distinct repressors define the expression boundaries of DV regulated gene. **Figure S6.** Temporal dynamics of DV enhancer and promoter chromatin states. **Figure S7.** Identification of DV relevant cell clusters from scRNA-seq data based on PROseq identified DV genes. **Figure S8.** Transcriptome-wide inference of burst kinetics from single-cell expression data.

**Additional file 2: Table S1 and S2.** DV regulated genes identified from *Toll* mutant PRO-seq, PRO-seq expression and Pol II promoter pausing data for DV regulated genes, and enriched GO terms for DV regulated genes identified from PRO-seq.

**Additional file 3: Table S3.** *De novo* identified motifs enriched at DV promoters.

**Additional file 4: Table S4.** DV regulated enhancers and promoters identified by epigenomic profiling of chromatin state.

**Additional file 5: Table S5 and S6.** Average expression and burst kinetic values inferred for DV and non-DV genes (according to *Toll* mutant PRO-seq) in DV-relevant scRNA-seq clusters from wild-type (PCNA:eGFP) embryos.

**Additional file 6: Table S7 and S10**. Pearson correlation, coefficient of determination (R^2^) and P-values associated with comparisons between tissue-specific *Toll* mutant epigenomic data at DV enhancers and promoters with burst frequency and size kinetics inferred from DV tissue clusters in scRNA-seq. Correlations are made for enhancer-paired DV genes that have a significant change in at least one burst kinetic between the cluster of activity and inactive clusters (n = 29 genes, 60 enhancers). *P*-values for statistical tests performed for each Figure panel are listed in Table S10.

**Additional file 7 Tables S8 and S9.** List of primer sequences, and PRO-seq, ChIP-seq, ATAC-seq and CUT&Tag library mapping information.

**Additional file 8.** Peer review history.

## Data Availability

The datasets generated during this study are available at Gene Expression Omnibus with the Accession Number GEO: GSE211220 [103]. The following datasets were also analyzed: GEO: GSE68983 [14, 15]; ArrayExpress: E-MTAB-9303 and E-MTAB-9304 [9]; GEO: GSE55306 [33][100]; GEO: GSE30757 [58]; GEO: GSE65441 [62]; GEO: GSE140722 [59]; GEO: GSE152598 and GEO: GSE152596 [60]; GEO: GSE152771 [61]; GEO: GSE58935 [57]; and GEO: GSE83851 [36].

## References

[1] Hong JW, Hendrix DA, Papatsenko D, Levine MS (2008). How the Dorsal gradient works: insights from postgenome technologies. Proc Natl Acad Sci U S A.

[2] Reeves GT, Stathopoulos A (2009). Graded dorsal and differential gene regulation in the Drosophila embryo. Cold Spring Harb Perspect Biol.

[3] Roth S, Stein D, Nusslein-Volhard C (1989). A gradient of nuclear localization of the dorsal protein determines dorsoventral pattern in the Drosophila embryo. Cell.

[4] Rushlow CA, Han K, Manley JL, Levine M (1989). The graded distribution of the dorsal morphogen is initiated by selective nuclear transport in Drosophila. Cell.

[5] Steward R (1989). Relocalization of the dorsal protein from the cytoplasm to the nucleus correlates with its function. Cell.

[6] Rusch J, Levine M (1996). Threshold responses to the dorsal regulatory gradient and the subdivision of primary tissue territories in the Drosophila embryo. Curr Opin Genet Dev.

[7] Espinola SM, Gotz M, Bellec M, Messina O, Fiche JB, Houbron C, Dejean M, Reim I, Cardozo Gizzi AM, Lagha M, Nollmann M (2021). Cis-regulatory chromatin loops arise before TADs and gene activation, and are independent of cell fate during early Drosophila development. Nat Genet.

[8] Ghavi-Helm Y, Klein FA, Pakozdi T, Ciglar L, Noordermeer D, Huber W, Furlong EE (2014). Enhancer loops appear stable during development and are associated with paused polymerase. Nature.

[9] Ing-Simmons E, Vaid R, Bing XY, Levine M, Mannervik M, Vaquerizas JM (2021). Independence of chromatin conformation and gene regulation during Drosophila dorsoventral patterning. Nat Genet.

[10] Core L, Adelman K (2019). Promoter-proximal pausing of RNA polymerase II: a nexus of gene regulation. Genes Dev.

[11] Gaertner B, Zeitlinger J (2014). RNA polymerase II pausing during development. Development.

[12] Zeitlinger J, Stark A, Kellis M, Hong JW, Nechaev S, Adelman K, Levine M, Young RA (2007). RNA polymerase stalling at developmental control genes in the Drosophila melanogaster embryo. Nat Genet.

[13] Lagha M, Bothma JP, Esposito E, Ng S, Stefanik L, Tsui C, Johnston J, Chen K, Gilmour DS, Zeitlinger J, Levine MS (2013). Paused Pol II coordinates tissue morphogenesis in the Drosophila embryo. Cell.

[14] Koenecke N, Johnston J, He Q, Meier S, Zeitlinger J (2017). Drosophila poised enhancers are generated during tissue patterning with the help of repression. Genome Res.

[15] Koenecke N, Johnston J, Gaertner B, Natarajan M, Zeitlinger J (2016). Genome-wide identification of Drosophila dorso-ventral enhancers by differential histone acetylation analysis. Genome Biol.

[16] Boija A, Mannervik M (2016). Initiation of diverse epigenetic states during nuclear programming of the Drosophila body plan. Proc Natl Acad Sci U S A.

[17] Holmqvist PH, Boija A, Philip P, Crona F, Stenberg P, Mannervik M (2012). Preferential genome targeting of the CBP co-activator by Rel and Smad proteins in early Drosophila melanogaster embryos. PLoS Genet.

[18] Zeitlinger J, Zinzen RP, Stark A, Kellis M, Zhang H, Young RA, Levine M (2007). Whole-genome ChIP-chip analysis of Dorsal, Twist, and Snail suggests integration of diverse patterning processes in the Drosophila embryo. Genes Dev.

[19] Kwak H, Fuda NJ, Core LJ, Lis JT (2013). Precise maps of RNA polymerase reveal how promoters direct initiation and pausing. Science.

[20] Judd J, Wojenski LA, Wainman LM, Tippens ND, Rice EJ, Dziubek A, Villafano GJ, Wissink EM, Versluis P, Bagepalli L, et al. A rapid, sensitive, scalable method for Precision Run-On sequencing (PRO-seq). bioRxiv. 2020.05.18.102277.

[21] Stathopoulos A, Van Drenth M, Erives A, Markstein M, Levine M (2002). Whole-genome analysis of dorsal-ventral patterning in the Drosophila embryo. Cell.

[22] Adelman K, Lis JT (2012). Promoter-proximal pausing of RNA polymerase II: emerging roles in metazoans. Nat Rev Genet.

[23] Kwasnieski JC, Orr-Weaver TL, Bartel DP (2019). Early genome activation in Drosophila is extensive with an initial tendency for aborted transcripts and retained introns. Genome Res.

[24] Chen K, Johnston J, Shao W, Meier S, Staber C, Zeitlinger J (2013). A global change in RNA polymerase II pausing during the Drosophila midblastula transition. Elife.

[25] Ramalingam V, Natarajan M, Johnston J, Zeitlinger J (2021). TATA and paused promoters active in differentiated tissues have distinct expression characteristics. Mol Syst Biol.

[26] Hendrix DA, Hong JW, Zeitlinger J, Rokhsar DS, Levine MS (2008). Promoter elements associated with RNA Pol II stalling in the Drosophila embryo. Proc Natl Acad Sci U S A.

[27] Sloutskin A, Danino YM, Orenstein Y, Zehavi Y, Doniger T, Shamir R, Juven-Gershon T (2015). ElemeNT: a computational tool for detecting core promoter elements. Transcription.

[28] Haberle V, Stark A (2018). Eukaryotic core promoters and the functional basis of transcription initiation. Nat Rev Mol Cell Biol.

[29] Creyghton MP, Cheng AW, Welstead GG, Kooistra T, Carey BW, Steine EJ, Hanna J, Lodato MA, Frampton GM, Sharp PA (2010). Histone H3K27ac separates active from poised enhancers and predicts developmental state. Proc Natl Acad Sci U S A.

[30] Heintzman ND, Hon GC, Hawkins RD, Kheradpour P, Stark A, Harp LF, Ye Z, Lee LK, Stuart RK, Ching CW (2009). Histone modifications at human enhancers reflect global cell-type-specific gene expression. Nature.

[31] Rada-Iglesias A, Bajpai R, Swigut T, Brugmann SA, Flynn RA, Wysocka J (2011). A unique chromatin signature uncovers early developmental enhancers in humans. Nature.

[32] Visel A, Blow MJ, Li Z, Zhang T, Akiyama JA, Holt A, Plajzer-Frick I, Shoukry M, Wright C, Chen F (2009). ChIP-seq accurately predicts tissue-specific activity of enhancers. Nature.

[33] He Q, Johnston J, Zeitlinger J (2015). ChIP-nexus enables improved detection of in vivo transcription factor binding footprints. Nat Biotechnol.

[34] Kvon EZ, Kazmar T, Stampfel G, Yanez-Cuna JO, Pagani M, Schernhuber K, Dickson BJ, Stark A (2014). Genome-scale functional characterization of Drosophila developmental enhancers in vivo. Nature.

[35] Boija A, Mahat DB, Zare A, Holmqvist PH, Philip P, Meyers DJ, Cole PA, Lis JT, Stenberg P, Mannervik M (2017). CBP Regulates Recruitment and Release of Promoter-Proximal RNA Polymerase II. Mol Cell.

[36] Blythe SA, Wieschaus EF. Establishment and maintenance of heritable chromatin structure during early Drosophila embryogenesis. Elife. 2016;5:e20148. 10.7554/eLife.20148 PMC5156528 27879204

[37] Reddington JP, Garfield DA, Sigalova OM, Karabacak Calviello A, Marco-Ferreres R, Girardot C, Viales RR, Degner JF, Ohler U, Furlong EEM (2020). Lineage-Resolved Enhancer and Promoter Usage during a Time Course of Embryogenesis. Dev Cell.

[38] Andersson R, Gebhard C, Miguel-Escalada I, Hoof I, Bornholdt J, Boyd M, Chen Y, Zhao X, Schmidl C, Suzuki T (2014). An atlas of active enhancers across human cell types and tissues. Nature.

[39] Tippens ND, Liang J, Leung AK, Wierbowski SD, Ozer A, Booth JG, Lis JT, Yu H (2020). Transcription imparts architecture, function and logic to enhancer units. Nat Genet.

[40] Bose DA, Donahue G, Reinberg D, Shiekhattar R, Bonasio R, Berger SL (2017). RNA Binding to CBP Stimulates Histone Acetylation and Transcription. Cell.

[41] Ortega E, Rengachari S, Ibrahim Z, Hoghoughi N, Gaucher J, Holehouse AS, Khochbin S, Panne D (2018). Transcription factor dimerization activates the p300 acetyltransferase. Nature.

[42] Schaukowitch K, Joo JY, Liu X, Watts JK, Martinez C, Kim TK (2014). Enhancer RNA facilitates NELF release from immediate early genes. Mol Cell.

[43] Mikhaylichenko O, Bondarenko V, Harnett D, Schor IE, Males M, Viales RR, Furlong EEM (2018). The degree of enhancer or promoter activity is reflected by the levels and directionality of eRNA transcription. Genes Dev.

[44] Hug CB, Grimaldi AG, Kruse K, Vaquerizas JM (2017). Chromatin Architecture Emerges during Zygotic Genome Activation Independent of Transcription. Cell.

[45] Ghavi-Helm Y, Jankowski A, Meiers S, Viales RR, Korbel JO, Furlong EEM (2019). Highly rearranged chromosomes reveal uncoupling between genome topology and gene expression. Nat Genet.

[46] Batut PJ, Bing XY, Sisco Z, Raimundo J, Levo M, Levine MS (2022). Genome organization controls transcriptional dynamics during development. Science.

[47] Price DH (2000). P-TEFb, a cyclin-dependent kinase controlling elongation by RNA polymerase II. Mol Cell Biol.

[48] Gressel S, Schwalb B, Decker TM, Qin W, Leonhardt H, Eick D, Cramer P. CDK9-dependent RNA polymerase II pausing controls transcription initiation. Elife. 2017;6:e29736. 10.7554/eLife.29736 PMC5669633 28994650

[49] Jang MK, Mochizuki K, Zhou M, Jeong HS, Brady JN, Ozato K (2005). The bromodomain protein Brd4 is a positive regulatory component of P-TEFb and stimulates RNA polymerase II-dependent transcription. Mol Cell.

[50] Yang Z, Yik JH, Chen R, He N, Jang MK, Ozato K, Zhou Q (2005). Recruitment of P-TEFb for stimulation of transcriptional elongation by the bromodomain protein Brd4. Mol Cell.

[51] Dey A, Chitsaz F, Abbasi A, Misteli T, Ozato K (2003). The double bromodomain protein Brd4 binds to acetylated chromatin during interphase and mitosis. Proc Natl Acad Sci U S A.

[52] Zhou Q, Li T, Price DH (2012). RNA polymerase II elongation control. Annu Rev Biochem.

[53] Dubnicoff T, Valentine SA, Chen G, Shi T, Lengyel JA, Paroush Z, Courey AJ (1997). Conversion of dorsal from an activator to a repressor by the global corepressor Groucho. Genes Dev.

[54] Papagianni A, Fores M, Shao W, He S, Koenecke N, Andreu MJ, Samper N, Paroush Z, Gonzalez-Crespo S, Zeitlinger J, Jimenez G (2018). Capicua controls Toll/IL-1 signaling targets independently of RTK regulation. Proc Natl Acad Sci U S A.

[55] Nibu Y, Zhang H, Levine M (1998). Interaction of short-range repressors with Drosophila CtBP in the embryo. Science.

[56] Qi D, Bergman M, Aihara H, Nibu Y, Mannervik M (2008). Drosophila Ebi mediates Snail-dependent transcriptional repression through HDAC3-induced histone deacetylation. EMBO J.

[57] Li XY, Harrison MM, Villalta JE, Kaplan T, Eisen MB. Establishment of regions of genomic activity during the Drosophila maternal to zygotic transition. Elife. 2014;3:e03737. 10.7554/eLife.03737 PMC4358338 25313869

[58] Harrison MM, Li XY, Kaplan T, Botchan MR, Eisen MB (2011). Zelda binding in the early Drosophila melanogaster embryo marks regions subsequently activated at the maternal-to-zygotic transition. PLoS Genet.

[59] Koromila T, Gao F, Iwasaki Y, He P, Pachter L, Gergen JP, Stathopoulos A. Odd-paired is a pioneer-like factor that coordinates with Zelda to control gene expression in embryos. Elife. 2020;9:e59610. 10.7554/eLife.59610 PMC7417190 32701060

[60] Duan J, Rieder L, Colonnetta MM, Huang A, McKenney M, Watters S, Deshpande G, Jordan W, Fawzi N, Larschan E. CLAMP and Zelda function together to promote Drosophila zygotic genome activation. Elife. 2021;10:e69937. 10.7554/eLife.69937 PMC8367384 34342574

[61] Gaskill MM, Gibson TJ, Larson ED, Harrison MM. GAF is essential for zygotic genome activation and chromatin accessibility in the early Drosophila embryo. Elife. 2021;10:e66668. 10.7554/eLife.66668 PMC8079149 33720012

[62] Sun Y, Nien CY, Chen K, Liu HY, Johnston J, Zeitlinger J, Rushlow C (2015). Zelda overcomes the high intrinsic nucleosome barrier at enhancers during Drosophila zygotic genome activation. Genome Res.

[63] Soluri IV, Zumerling LM, Payan Parra OA, Clark EG, Blythe SA. Zygotic pioneer factor activity of Odd-paired/Zic is necessary for late function of the Drosophila segmentation network. Elife. 2020;9:e53916. 10.7554/eLife.53916 PMC7190358 32347792

[64] Rodriguez J, Larson DR (2020). Transcription in Living Cells: Molecular Mechanisms of Bursting. Annu Rev Biochem.

[65] Larsson AJM, Johnsson P, Hagemann-Jensen M, Hartmanis L, Faridani OR, Reinius B, Segerstolpe A, Rivera CM, Ren B, Sandberg R (2019). Genomic encoding of transcriptional burst kinetics. Nature.

[66] Forbes Beadle L, Zhou H, Rattray M, Ashe HL (2023). Modulation of transcription burst amplitude underpins dosage compensation in the Drosophila embryo. Cell Rep.

[67] Hoppe C, Bowles JR, Minchington TG, Sutcliffe C, Upadhyai P, Rattray M, Ashe HL (2020). Modulation of the Promoter Activation Rate Dictates the Transcriptional Response to Graded BMP Signaling Levels in the Drosophila Embryo. Dev Cell.

[68] Nicolas D, Zoller B, Suter DM, Naef F (2018). Modulation of transcriptional burst frequency by histone acetylation. Proc Natl Acad Sci U S A.

[69] Zoller B, Little SC, Gregor T (2018). Diverse Spatial Expression Patterns Emerge from Unified Kinetics of Transcriptional Bursting. Cell.

[70] Diamant G, Dikstein R (2013). Transcriptional control by NF-kappaB: elongation in focus. Biochim Biophys Acta.

[71] Vaid R, Wen J, Mannervik M (2020). Release of promoter-proximal paused Pol II in response to histone deacetylase inhibition. Nucleic Acids Res.

[72] Joo YJ, Ficarro SB, Soares LM, Chun Y, Marto JA, Buratowski S (2017). Downstream promoter interactions of TFIID TAFs facilitate transcription reinitiation. Genes Dev.

[73] Tantale K, Garcia-Oliver E, Robert MC, L’Hostis A, Yang Y, Tsanov N, Topno R, Gostan T, Kozulic-Pirher A, Basu-Shrivastava M, et al. Stochastic pausing at latent HIV-1 promoters generates transcriptional bursting. Nat Commun. 2021;12:4503. 10.1038/s41467-021-24462-5 PMC8302722 34301927

[74] Pimmett VL, Dejean M, Fernandez C, Trullo A, Bertrand E, Radulescu O, Lagha M (2021). Quantitative imaging of transcription in living Drosophila embryos reveals the impact of core promoter motifs on promoter state dynamics. Nat Commun.

[75] Falo-Sanjuan J, Lammers NC, Garcia HG, Bray SJ (2019). Enhancer priming enables fast and sustained transcriptional responses to notch signaling. Dev Cell.

[76] Lee C, Shin H, Kimble J (2019). Dynamics of notch-dependent transcriptional bursting in its native context. Dev Cell.

[77] Fukaya T, Lim B, Levine M (2016). Enhancer control of transcriptional bursting. Cell.

[78] Fukaya T (2021). Dynamic regulation of anterior-posterior patterning genes in living Drosophila embryos. Curr Biol.

[79] Lammers NC, Galstyan V, Reimer A, Medin SA, Wiggins CH, Garcia HG (2020). Multimodal transcriptional control of pattern formation in embryonic development. Proc Natl Acad Sci U S A.

[80] Schulz KN, Bondra ER, Moshe A, Villalta JE, Lieb JD, Kaplan T, McKay DJ, Harrison MM (2015). Zelda is differentially required for chromatin accessibility, transcription factor binding, and gene expression in the early Drosophila embryo. Genome Res.

[81] Lu H, Yu D, Hansen AS, Ganguly S, Liu R, Heckert A, Darzacq X, Zhou Q (2018). Phase-separation mechanism for C-terminal hyperphosphorylation of RNA polymerase II. Nature.

[82] Koch R, Ledermann R, Urwyler O, Heller M, Suter B (2009). Systematic functional analysis of Bicaudal-D serine phosphorylation and intragenic suppression of a female sterile allele of BicD. PLoS One.

[83] Jiang J, Hoey T, Levine M (1991). Autoregulation of a segmentation gene in Drosophila: combinatorial interaction of the even-skipped homeo box protein with a distal enhancer element. Genes Dev.

[84] Tautz D, Pfeifle C (1989). A non-radioactive in situ hybridization method for the localization of specific RNAs in Drosophila embryos reveals translational control of the segmentation gene hunchback. Chromosoma.

[85] Tomancak P, Beaton A, Weiszmann R, Kwan E, Shu S, Lewis SE, Richards S, Ashburner M, Hartenstein V, Celniker SE, Rubin GM (2002). Systematic determination of patterns of gene expression during Drosophila embryogenesis. Genome Biol.

[86] Tomancak P, Berman BP, Beaton A, Weiszmann R, Kwan E, Hartenstein V, Celniker SE, Rubin GM (2007). Global analysis of patterns of gene expression during Drosophila embryogenesis. Genome Biol.

[87] Hammonds AS, Bristow CA, Fisher WW, Weiszmann R, Wu S, Hartenstein V, Kellis M, Yu B, Frise E, Celniker SE (2013). Spatial expression of transcription factors in Drosophila embryonic organ development. Genome Biol.

[88] Buenrostro JD, Giresi PG, Zaba LC, Chang HY, Greenleaf WJ (2013). Transposition of native chromatin for fast and sensitive epigenomic profiling of open chromatin, DNA-binding proteins and nucleosome position. Nat Methods.

[89] Blythe SA, Wieschaus EF (2015). Zygotic genome activation triggers the DNA replication checkpoint at the midblastula transition. Cell.

[90] Hanyu-Nakamura K, Sonobe-Nojima H, Tanigawa A, Lasko P, Nakamura A (2008). Drosophila Pgc protein inhibits P-TEFb recruitment to chromatin in primordial germ cells. Nature.

[91] Kockmann T, Gerstung M, Schlumpf T, Xhinzhou Z, Hess D, Beerenwinkel N, Beisel C, Paro R (2013). The BET protein FSH functionally interacts with ASH1 to orchestrate global gene activity in Drosophila. Genome Biol.

[92] Hainer SJ, Fazzio TG (2019). High-Resolution Chromatin Profiling Using CUT&RUN. Curr Protoc Mol Biol.

[93] Kaya-Okur HS, Wu SJ, Codomo CA, Pledger ES, Bryson TD, Henikoff JG, Ahmad K, Henikoff S (1930). CUT&Tag for efficient epigenomic profiling of small samples and single cells. Nat Commun.

[94] Langmead B, Salzberg SL (2012). Fast gapped-read alignment with Bowtie 2. Nat Methods.

[95] Ramirez F, Ryan DP, Gruning B, Bhardwaj V, Kilpert F, Richter AS, Heyne S, Dundar F, Manke T (2016). deepTools2: a next generation web server for deep-sequencing data analysis. Nucleic Acids Res.

[96] Liao Y, Smyth GK, Shi W (2014). featureCounts: an efficient general purpose program for assigning sequence reads to genomic features. Bioinformatics.

[97] Love MI, Huber W, Anders S (2014). Moderated estimation of fold change and dispersion for RNA-seq data with DESeq2. Genome Biol.

[98] Stathopoulos A, Levine M (2002). Whole-genome expression profiles identify gene batteries in Drosophila. Dev Cell.

[99] Saunders A, Core LJ, Sutcliffe C, Lis JT, Ashe HL (2013). Extensive polymerase pausing during Drosophila axis patterning enables high-level and pliable transcription. Genes Dev.

[100] Quinlan AR, Hall IM (2010). BEDTools: a flexible suite of utilities for comparing genomic features. Bioinformatics.

[101] Hao Y, Hao S, Andersen-Nissen E, Mauck WM, Zheng S, Butler A, Lee MJ, Wilk AJ, Darby C, Zager M (2021). Integrated analysis of multimodal single-cell data. Cell.

[102] Satija R, Farrell JA, Gennert D, Schier AF, Regev A (2015). Spatial reconstruction of single-cell gene expression data. Nat Biotechnol.

[103] Hunt G, Vaid R, Pirogov S, Pfab A, Ziegenhain C, Sandberg R, Reimegård J, Mannervik: Tissue-specific RNA Polymerase II promoter-proximal pause release and burst kinetics in a *Drosophila* embryonic patterning network. GSE211220. Gene Expression Omnibus. 2023. https://www.ncbi.nlm.nih.gov/geo/query/acc.cgi?acc=GSE211220. 10.1186/s13059-023-03135-0 PMC10763363 38166964

[104] Kerpedjiev P, Abdennur N, Lekschas F, McCallum C, Dinkla K, Strobelt H, Luber JM, Ouellette SB, Azhir A, Kumar N (2018). HiGlass: web-based visual exploration and analysis of genome interaction maps. Genome Biol.

