# Supplementary material for: Tissue-specific RNA Polymerase II promoter-proximal pause release and burst kinetics in a Drosophila embryonic patterning network
Source: Genome Biol. 2024 Jan 2;25:2. doi: 10.1186/s13059-023-03135-0 (PMC10763363; doi:10.1186/s13059-023-03135-0)
Supplement: Supplementary file 1 — Additional file 1: Figure S1. PRO-seq identifies DV regulated genes with promoter-proximal paused Pol II that persists across tissue types and developmental stages. Figure S2. Characterization of tissue-specific DV enhancers identified by epigenomic profiling of chromatin state. Figure S3. Temporal dynamics of chromatin accessibility and genome organization at DV genes [104]. Figure S4. Tissue-specific P-TEFb and BRD4/fs(1)h recruitment to DV genes. Figure S5. Distinct repressors define the expression boundaries of DV regulated gene. Figure S6. Temporal dynamics of DV enhancer and promoter chromatin states. Figure S7. Identification of DV relevant cell clusters from scRNA-seq data based on PROseq identified DV genes. Figure S8. Transcriptome-wide inference of burst kinetics from single-cell expression data. [file 13059_2023_3135_MOESM1_ESM.pdf]

Figure S1

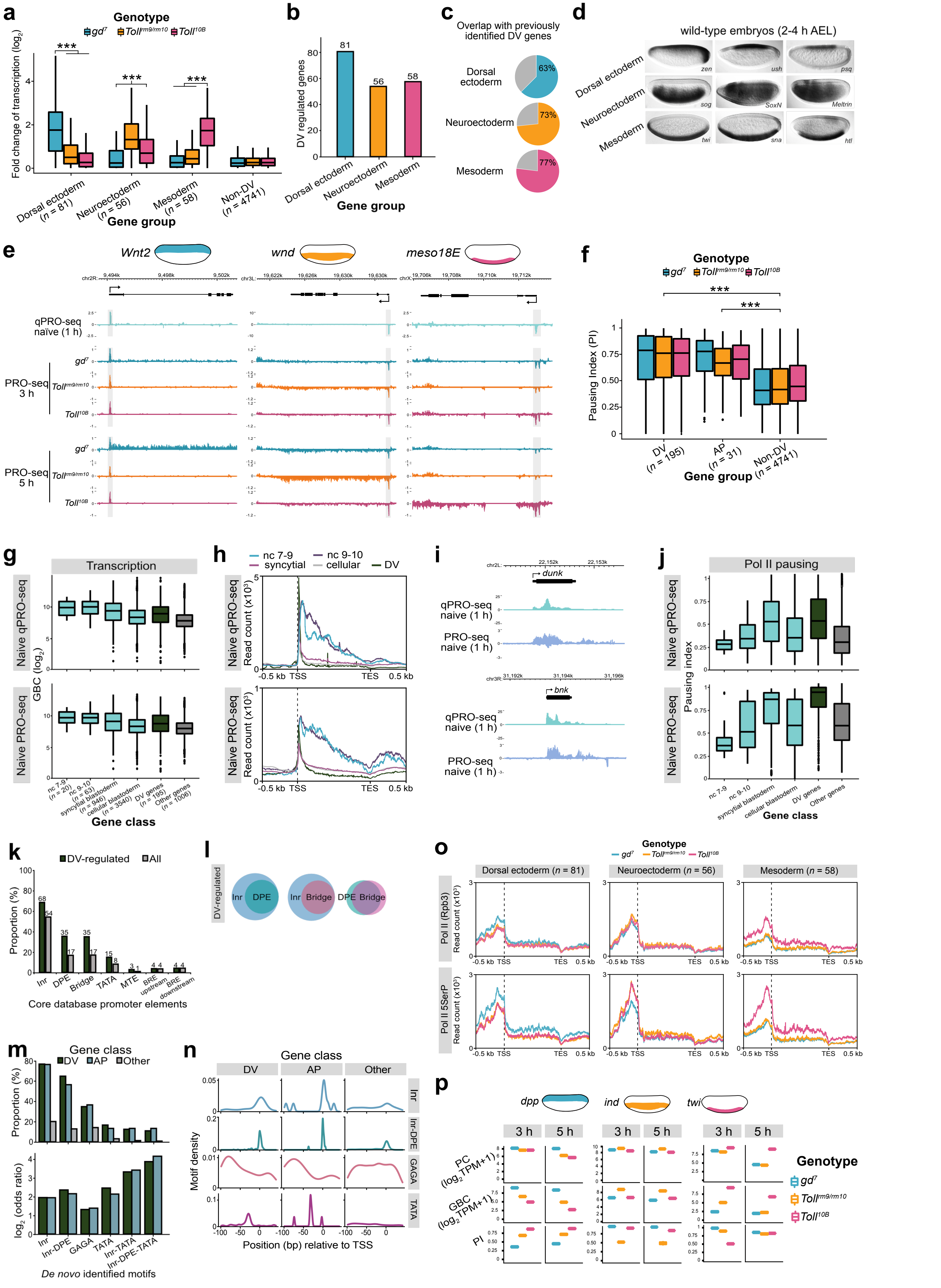
a
Genotype
gd7
Tollrm9/rm10
Toll10B
Fold change of transcription (log2)
Dorsal ectoderm (n = 81)
Neuroectoderm (n = 56)
Mesoderm (n = 58)
Non-DV (n = 4741)
Gene group
b
DV regulated genes
81
56
58
Dorsal ectoderm
Neuroectoderm
Mesoderm
Gene group
c
Overlap with previously identified DV genes
Dorsal ectoderm
63%
Neuroectoderm
73%
Mesoderm
77%
d
wild-type embryos (2-4 h AEL)
Dorsal ectoderm
Neuroectoderm
Mesoderm
zen
ush
psq
sog
SoxN
Meltrin
twi
sna
htl
e
Wnt2
wnd
meso18E
qPRO-seq naïve (1 h)
PRO-seq 3 h
PRO-seq 5 h
f
Genotype
Pausing Index (PI)
DV (n = 195)
AP (n = 31)
Non-DV (n = 4741)
Gene group
g
Transcription
Naive qPRO-seq
Naive PRO-seq
GBC (log2)
nc 7-9 (n = 20)
nc 9-10 (n = 63)
syncytial blastoderm (n = 946)
cellular blastoderm (n = 3540)
DV genes (n = 195)
Other genes (n = 1006)
Gene class
h
nc 7-9
nc 9-10
syncytial
cellular
DV
Naive qPRO-seq Read count (x10³)
Naive PRO-seq Read count (x10³)
-0.5 kb TSS TES 0.5 kb
i
dunk
qPRO-seq naive (1 h)
PRO-seq naive (1 h)
bnk
j
Pol II pausing
Naive qPRO-seq
Naive PRO-seq
Pausing index
nc 7-9
nc 9-10
syncytial blastoderm
cellular blastoderm
DV genes
Other genes
Gene class
k
DV-regulated
All
Proportion (%)
Inr
DPE
Bridge
TATA
MTE
BRE upstream
BRE downstream
Core database promoter elements
l
DV-regulated
Inr
DPE
Bridge
o
Genotype
Dorsal ectoderm (n = 81)
Neuroectoderm (n = 56)
Mesoderm (n = 58)
Pol II (Rpb3) Read count (x10³)
Pol II 5SerP Read count (x10³)
m
Gene class
DV
AP
Other
Proportion (%)
log2 (odds ratio)
Inr
Inr-DPE
GAGA
TATA
Inr-TATA
Inr-DPE-TATA
De novo identified motifs
n
Gene class
DV
AP
Other
Motif density
Position (bp) relative to TSS
p
dpp
ind
twi
3 h
5 h
PC (log2TPM+1)
GBC (log2TPM+1)
PI
Genotype

Figure S1. PRO-seq identifies DV regulated genes with promoter-proximal paused Pol II that persists across tissue types and developmental stages. a) Fold change (log_2) of tissue-specific transcription (from PRO-seq derived gene body read counts (GBC)) in *Toll* mutants for DV genes grouped by the tissue of expression (dorsal ectoderm n = 81, neuroectoderm n = 56, mesoderm n = 58) and non-DV(n = 4741) genes. Significant differences in DV gene group tissue-specific transcription between *Toll* mutants (Wilcoxon signed-rank test) are indicated by asterisks, * = $P < 0.05$, ** = $P < 0.01$, *** = $P < 0.001$ **b)** The number of differentially expressed DV genes identified by PRO-seq, grouped by the tissue of expression. **c)** The overlap (%) of PRO-seq identified DV regulated genes with DV genes previously identified by whole genome microarray [21]. **d)** Images of whole mount *in situ* hybridization in wild-type embryos (2-4 h AEL) with probes for mRNAs of representative DV regulated genes identified by PRO-seq. Images of *zen, ush, SoxN, Meltrin, twi, sna* and *htl* were obtained from the BDGP database [84-86]. **e)** Genome browser shots of stranded PRO-seq signal (RPKM x10^3) at *Wnt2*, *wnd* and *meso18E*. Promoters are shaded gray. **f)** Pausing index (PI) of DV regulated genes compared to anterior-posterior (AP) [98] and non-DV genes in *Toll* mutant PRO-seq. *P*-values are from the Wilcoxon rank-sum test. **g)** Comparisons of the PRO-seq and qPRO-seq signal (GBC (log_2 TPM+1)) of zygotic genes expressed at different embryonic stages [23] and DV genes from naïve wild-type embryos. **h)** Metagene plots of naïve qPRO- and PRO-seq signal for the gene classes from **g**. **i)** Genome browser shots of stranded qPRO- and PRO-seq signal (RPKM x10^3) from naïve wild-type embryos at representative nc 7-9 expressed genes. **j)** Comparisons of the PI of the gene classes from **g** between naïve wild-type embryos in qPRO- and PRO-seq. **k)** Representation (%) of core promoter elements from the CORE database [27] at the promoters of DV regulated (n = 195) and all (n = 13,965) genes. **l)** Venn diagrams of the overlap of DV regulated genes with Inr, DPE and Bridge motif. **m)** Representation (%) and odds ratio (log_2) for *de novo* identified motifs at the promoters of DV, AP and other genes. **n)** De novo identified motif densities for Inr, Inr-DPE, GAGA and TATA at the promoters (from 100 bp upstream to 50 bp downstream of the TSS) of DV, AP and other genes. **o)** Metagene plots of *Toll* mutant Pol II (Rpb3) and serine 5 phosphorylated (5SerP) Pol II CUT&Tag (2-4 h AEL) at DV regulated genes. **p)** PRO-seq promoter counts (PC) (log_2 TPM+1), GBC and PI for representative DV genes from 2.5-3 h and 4.5-5 h AEL *Toll* mutants.

Figure S2

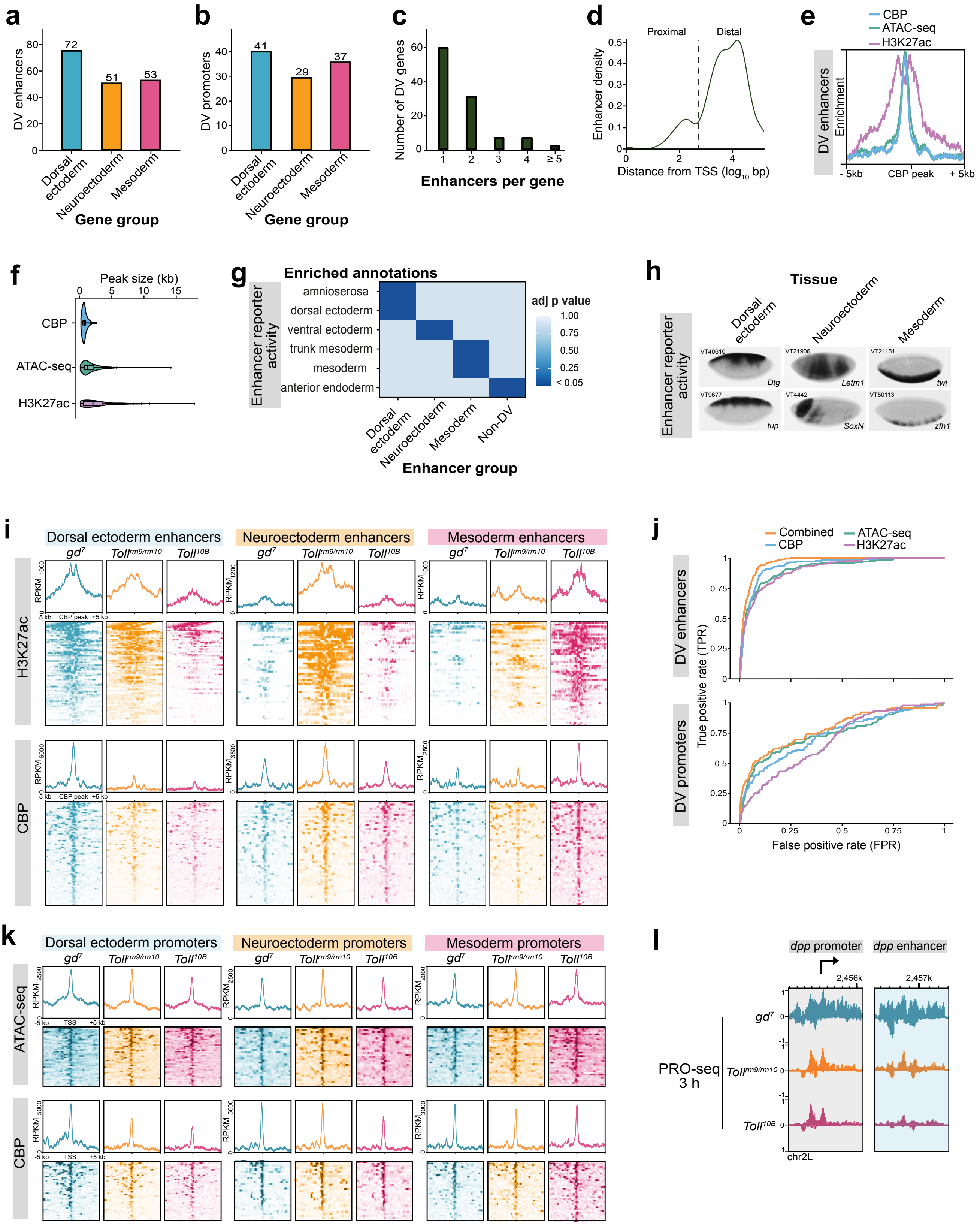
a
DV enhancers
72
51
53
Dorsal ectoderm
Neuroectoderm
Mesoderm
Gene group
b
DV promoters
41
29
37
Dorsal ectoderm
Neuroectoderm
Mesoderm
Gene group
c
Number of DV genes
Enhancers per gene
d
Proximal
Distal
Enhancer density
Distance from TSS (log10 bp)
e
CBP
ATAC-seq
H3K27ac
DV enhancers
Enrichment
- 5kb
CBP peak
+ 5kb
f
Peak size (kb)
CBP
ATAC-seq
H3K27ac
g
Enriched annotations
Enhancer reporter activity
amnioserosa
dorsal ectoderm
ventral ectoderm
trunk mesoderm
mesoderm
anterior endoderm
adj p value
1.00
0.75
0.50
0.25
< 0.05
Dorsal ectoderm
Neuroectoderm
Mesoderm
Non-DV
Enhancer group
h
Tissue
Dorsal ectoderm
Neuroectoderm
Mesoderm
Enhancer reporter activity
VT40610
Dtg
VT21906
Letm1
VT21151
twi
VT9677
tup
VT4442
SoxN
VT50113
zfh1
i
Dorsal ectoderm enhancers
Neuroectoderm enhancers
Mesoderm enhancers
gd7
Tollrm9/rm10
Toll10B
H3K27ac
CBP
RPKM
CBP peak
j
Combined
CBP
ATAC-seq
H3K27ac
DV enhancers
DV promoters
True positive rate (TPR)
False positive rate (FPR)
k
Dorsal ectoderm promoters
Neuroectoderm promoters
Mesoderm promoters
gd7
Tollrm9/rm10
Toll10B
ATAC-seq
CBP
RPKM
TSS
l
dpp promoter
dpp enhancer
2,456k
2,457k
PRO-seq 3 h
gd7
Tollrm9/rm10
Toll10B
chr2L

Figure S2. Characterization of tissue-specific DV enhancers identified by epigenomic profiling of chromatin state. a) Counts for the identified tissue-specific DV enhancers and **(b)** corresponding promoters partitioned by the tissue of target gene expression. **c)** Enhancer-linked DV genes binned by the number of paired enhancers. **d)** Distribution of DV enhancer density in relation to genomic distance from the TSS of target genes. Enhancers with a distance ≤ 700 bp from the TSS were classified as proximal (n = 36) and above this threshold defined as distal (n = 160). For enhancers linked to multiple separate genes or genes with more than one differentially expressed isoform, the distance to each TSS was measured. **e)** Metagene profiles of CBP, ATAC-seq and H3K27ac enrichment at DV enhancers (± 5 kb of CBP peak). **f)** Violin plots of the genomic length distributions (kb) of peaks called for CBP, ATAC-seq and H3K27ac overlapping DV enhancers. **g)** Enriched annotations associated with the enhancer reporter activities of non-coding genomic fragments (Vienna Tiles, VT) that overlap DV enhancers separated by the tissue of activity [33]. **h)** Images of whole-mount *in situ* hybridization of lacZ reporter activity driven by representative VT enhancer fragments that overlap identified DV enhancers. **i)** Metagene profiles and heatmaps (± 5 kb of CBP peak) of *Toll* mutant H3K27ac and CBP ChIP-seq signal (RPKM) at dorsal ectoderm, neuroectoderm and mesoderm enhancers. **j)** Receiver operating characteristic (ROC) curves for ATAC-seq, CBP and H3K27ac individually and combined at DV enhancers and promoters. **k)** Metagene profiles and heatmaps (± 5 kb of TSS) of *Toll* mutant CBP and ATAC-seq signal (RPKM) at dorsal ectoderm, neuroectoderm and mesoderm promoters. **l)** Genome browser closeups of *Toll* mutant PRO-seq (3 h AEL) signal at the *dpp* promoter and enhancer.

Figure S3

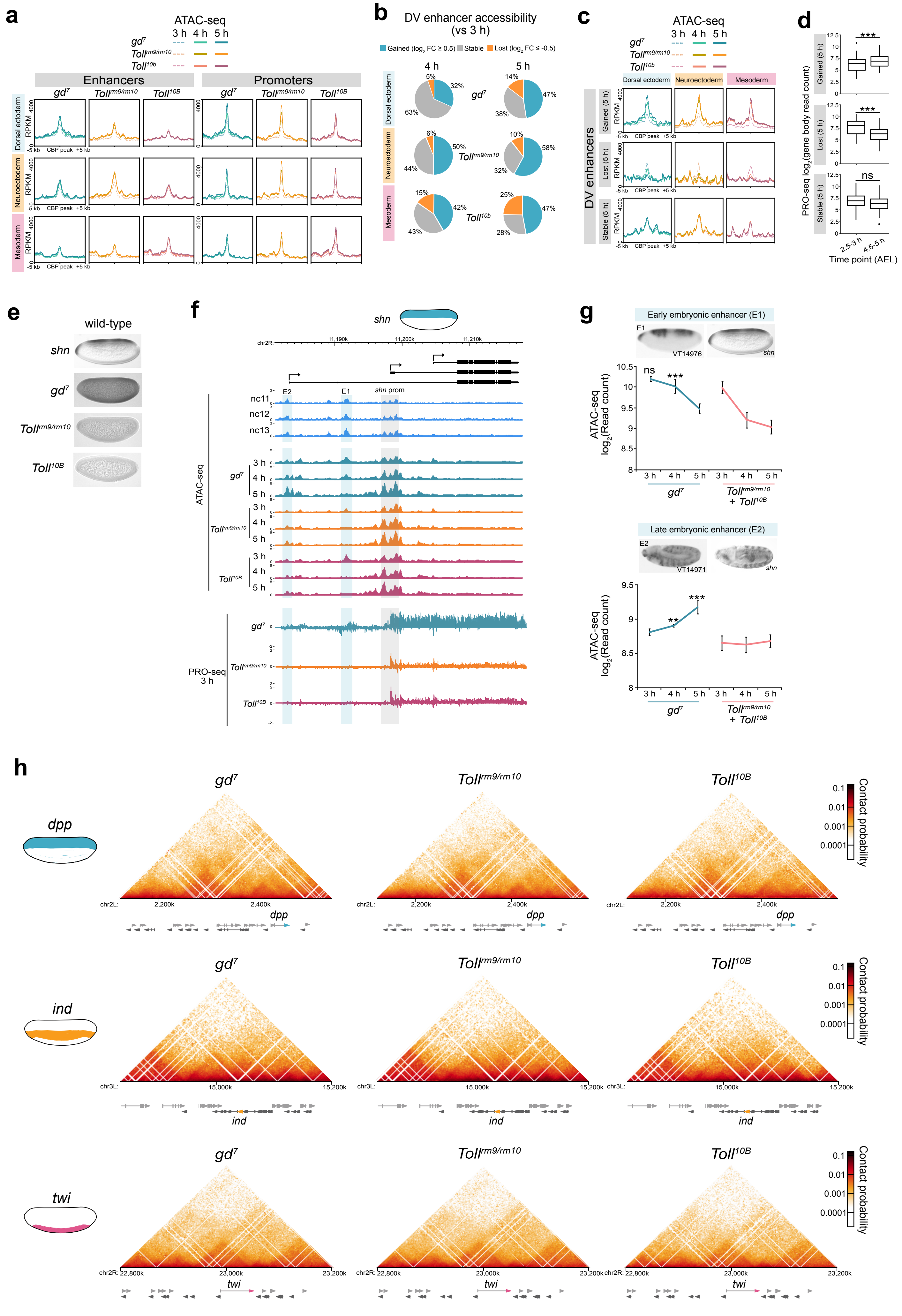

a
ATAC-seq
3 h 4 h 5 h
gd7
Tollrm9/rm10
Toll10b
Enhancers
Promoters
gd7
Tollrm9/rm10
Toll10B
Dorsal ectoderm
Neuroectoderm
Mesoderm
RPKM
4000
0
-5 kb
CBP peak
+5 kb
b
DV enhancer accessibility
(vs 3 h)
Gained (log2 FC ≥ 0.5)
Stable
Lost (log2 FC ≤ -0.5)
4 h
5 h
5%
32%
63%
14%
47%
38%
6%
50%
44%
10%
58%
32%
15%
42%
43%
25%
47%
28%
c
DV enhancers
Dorsal ectoderm
Neuroectoderm
Mesoderm
Gained (5 h)
Lost (5 h)
Stable (5 h)
d
PRO-seq log2(gene body read count)
***
ns
2.5-3 h
4.5-5 h
Time point (AEL)
e
wild-type
shn
gd7
Tollrm9/rm10
Toll10B
f
shn
chr2R:
11,190k
11,200k
11,210k
E2
E1
shn prom
nc11
nc12
nc13
ATAC-seq
PRO-seq
3 h
g
Early embryonic enhancer (E1)
E1
VT14976
shn
ATAC-seq
log2(Read count)
ns
***
**
Late embryonic enhancer (E2)
E2
VT14971
Tollrm9/rm10
+ Toll10B
h
dpp
ind
twi
Contact probability
0.1
0.01
0.001
0.0001
chr2L:
2,200k
2,400k
chr3L:
15,000k
15,200k
chr2R:
22,800k
23,000k
23,200k

Figure S3. Temporal dynamics of chromatin accessibility and genome organization at DV genes. a) Metagene plots of *Toll* mutant embryo (3 h, 4 h and 5 h AEL) ATAC-seq signal (RPKM) at DV enhancers and promoters partitioned by the tissue of target gene expression. **b)** Proportion (%) of dorsal ectoderm, neuroectoderm and mesoderm enhancers with gained ($\log_2$ fold change (FC) ≥ 0.5), lost ($\log_2$ FC ≤ -0.5) or maintained stable chromatin accessibility (ATAC-seq) in 4 h and 5 h AEL embryos relative to 3 h. The accessibility was measured from *gd*^{7} at dorsal ectoderm enhancers, *Toll*$^{rm9/rm10}$ at neuroectoderm enhancers and *Toll*^{10B} at mesoderm enhancers. **c)** Metagene plots of *Toll* mutant ATAC-seq signal (3 h, 4 h and 5 h AEL) at DV enhancers partitioned by the tissue of target gene activity and the change of accessibility (5 h relative to 3 h AEL). **d)** Boxplots of early (2.5-3 h) and late (4.5-5 h) *Toll* mutant PRO-seq gene body expression ($\log_2$ read count) for DV genes associated with enhancers that have gained ($n = 57$), lost ($n = 24$) or stable accessibility ($n = 39$) at 5 h AEL relative to 3 h AEL. Expression was measured for genes in the tissue mutant of activity. *P*-values (Wilcoxon rank-sum test) show significant differences in expression (2.5-3 h vs 4.5-5 h). **e)** Images of whole-mount *in situ* hybridization with a probe against *schnurri* (*shn)* mRNA in wild-type and *Toll* mutant embryos (2-4 h AEL). **f)** Genome browser shot of ATAC-seq signal from wild-type naïve embryos (nc 11, 12 and 13) [35] and *Toll* mutant embryos (3 h, 4 h and 5 h AEL) and *Toll* mutant PRO-seq (3 h AEL) at the *shn* locus. The major *shn* promoter active during early embryogenesis and associated enhancers are denoted. **g)** (Top) Images of whole-mount *in situ* hybridization of lacZ reporter activity driven by VT enhancer fragments that overlap *shn* enhancers with predicted early (E1) and late (E2) embryonic activity, alongside *in situ* hybridization with a probe against endogenous *shn* mRNA in wild-type embryos at corresponding developmental stages. (Bottom) Plots of the mean ATAC-seq signal ($\log_2$ read count) at the E1 and E2 enhancers (3 h, 4 h and 5 h AEL) in the *gd*^{7} mutant compared to *Toll*$^{rm9/rm10}$ and *Toll*^{10B} ($n = 3$). Error bars show SEM. Significant differences in the accessibility between *gd*^{7} and *Toll*$^{rm9/rm10}$/*Toll*^{10B} (two tailed, unpaired t-test) are indicated by asterisks, * = $P < 0.05$, ** = $P < 0.01$, *** = $P < 0.001$. **h)** Normalized Hi-C contact probabilities (5-kb resolution) from Ing-Simmons E, Vaid R, Bing XY, Levine M, Mannervik M and Vaquerizas JM [9] for representative DV regulated genes (*dpp*, *ind* and *twi*) in *Toll* mutant (2-3 h AEL) embryos visualized with HiGlass [103].

Figure S4

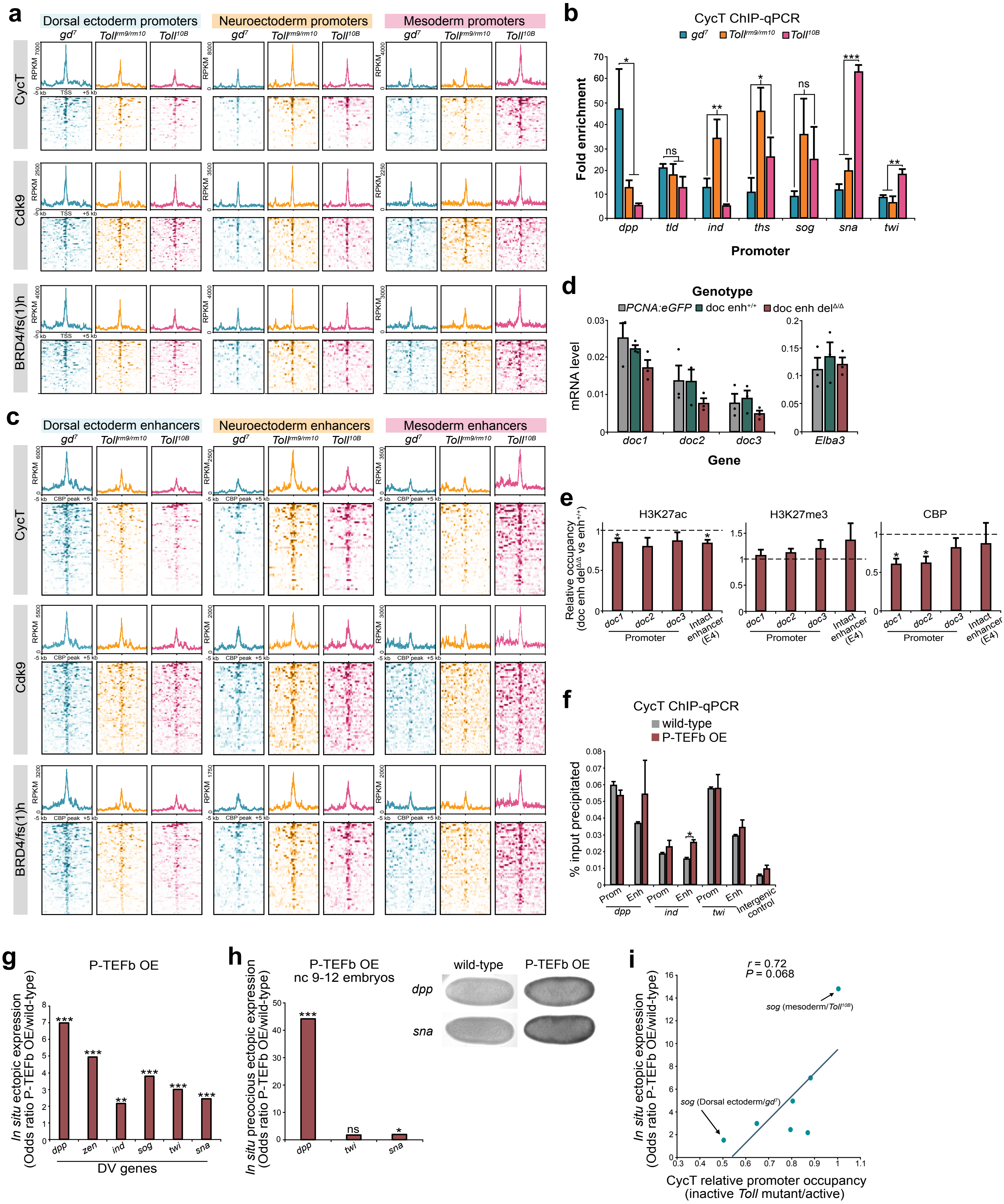

a
Dorsal ectoderm promoters
Neuroectoderm promoters
Mesoderm promoters
CycT
Cdk9
BRD4/fs(1)h
RPKM
TSS
b
CycT ChIP-qPCR
Fold enrichment
Promoter
dpp
tld
ind
ths
sog
sna
twi
c
Dorsal ectoderm enhancers
Neuroectoderm enhancers
Mesoderm enhancers
CBP peak
d
Genotype
PCNA:eGFP
mRNA level
doc1
doc2
doc3
Elba3
Gene
e
H3K27ac
H3K27me3
CBP
Relative occupancy
Intact enhancer (E4)
f
CycT ChIP-qPCR
wild-type
P-TEFb OE
% input precipitated
Prom
Enh
Intergenic control
g
P-TEFb OE
In situ ectopic expression (Odds ratio P-TEFb OE/wild-type)
DV genes
zen
h
P-TEFb OE nc 9-12 embryos
In situ precocious ectopic expression (Odds ratio P-TEFb OE/wild-type)
wild-type
P-TEFb OE
i
r = 0.72
P = 0.068
CycT relative promoter occupancy (inactive Toll mutant/active)

Figure S4. Tissue-specific P-TEFb and BRD4/fs(1)h recruitment to DV genes. a) Metagene profiles and heatmaps (± 5 kb of TSS) of *Toll* mutant CUT&Tag (2-4 h AEL) at DV regulated genes with antibodies against the P-TEFb subunits CycT and Cdk9 and the co-activator BRD4/fs(1)h. **b)** ChIP-qPCR validation of tissue-specific enrichment of CycT at DV regulated gene promoters (*dpp*, *tld*, *sog*, *ths*, *ind*, *sna* and *twi*) in *Toll* mutants. Enrichment is measured at DV targets relative to the signal at representative intergenic regions. Error bars show SEM. Significant differences in enrichment at promoters between the mutant that expresses the gene versus the mutants that do not (two tailed, unpaired t-test) are indicated by asterisks, * = $P < 0.05$, ** = $P < 0.01$, *** = $P < 0.001$. **c)** Metagene profiles and heatmaps (± 5 kb of CBP peak) of *Toll* mutant CycT, Cdk9 and BRD4/fs(1)h CUT&Tag signal (RPKM) at dorsal ectoderm, neuroectoderm and mesoderm enhancers. **d)** RT-qPCR quantification of *Doc1, Doc2, Doc3* and *Elba3* mRNA levels (relative to *RpL32*) from *Doc enhancer (enh)* $del^{\Delta/\Delta}$ embryos (2-4 h AEL) and *PCNA-eGFP* and $enh^{+/+}$ embryos ($n = 3$). **e)** ChIP-qPCR showing the enrichment of H3K27ac, H3K27me3 and CBP at the promoters of *Doc1*, *Doc2* and *Doc3* in *Doc enh* $del^{\Delta/\Delta}$ embryos (2-4 h AEL) relative to $enh^{+/+}$ embryos ($n = 3\text{-}4$). Relative occupancy is also shown at an intact *Doc* enhancer (E4). Error bars show SEM. Significant differences in occupancy (two tailed, unpaired t-test) are indicated by asterisks, * = $P < 0.05$. **f)** ChIP-qPCR showing the % input precipitated by anti-CycT at the promoters and enhancers of DV regulated genes (*dpp*, *ind* and *twi*) and an intergenic control region in chromatin from wild-type and P-TEFb maternally overexpressed (OE) embryos (2-4 h AEL). Error bars show SEM. Significant differences in the % input precipitated at targets between wild-type and P-TEFb OE embryos (two tailed, unpaired t-test) are indicated by asterisks (* = $P < 0.05$). **g)** Odds ratios measuring the strength of association between P-TEFb OE and *in situ* ectopic expression relative to wild-type embryos, for DV regulated genes. *P*-values are from Fisher's exact test. **h)** Odds ratios as in **g** but measuring precocious expression in nc 9-12 P-TEFb OE embryos. Representative images of whole mount *in situ* hybridization with *dpp* and *sna* probes. **i)** Correlation between *in situ* ectopic expression of DV genes in P-TEFb OE embryos (odds ratio P-TEFb OE/wild-type) and the relative CUT&Tag promoter occupancy of CycT at the same DV genes in inactive Toll mutant embryos relative to active. Values for *sog* were obtained in the two inactive tissues (dorsal ectoderm and mesoderm) separately. The Pearson correlation coefficient (*r*) and associated *P*-value are denoted.

Figure S5

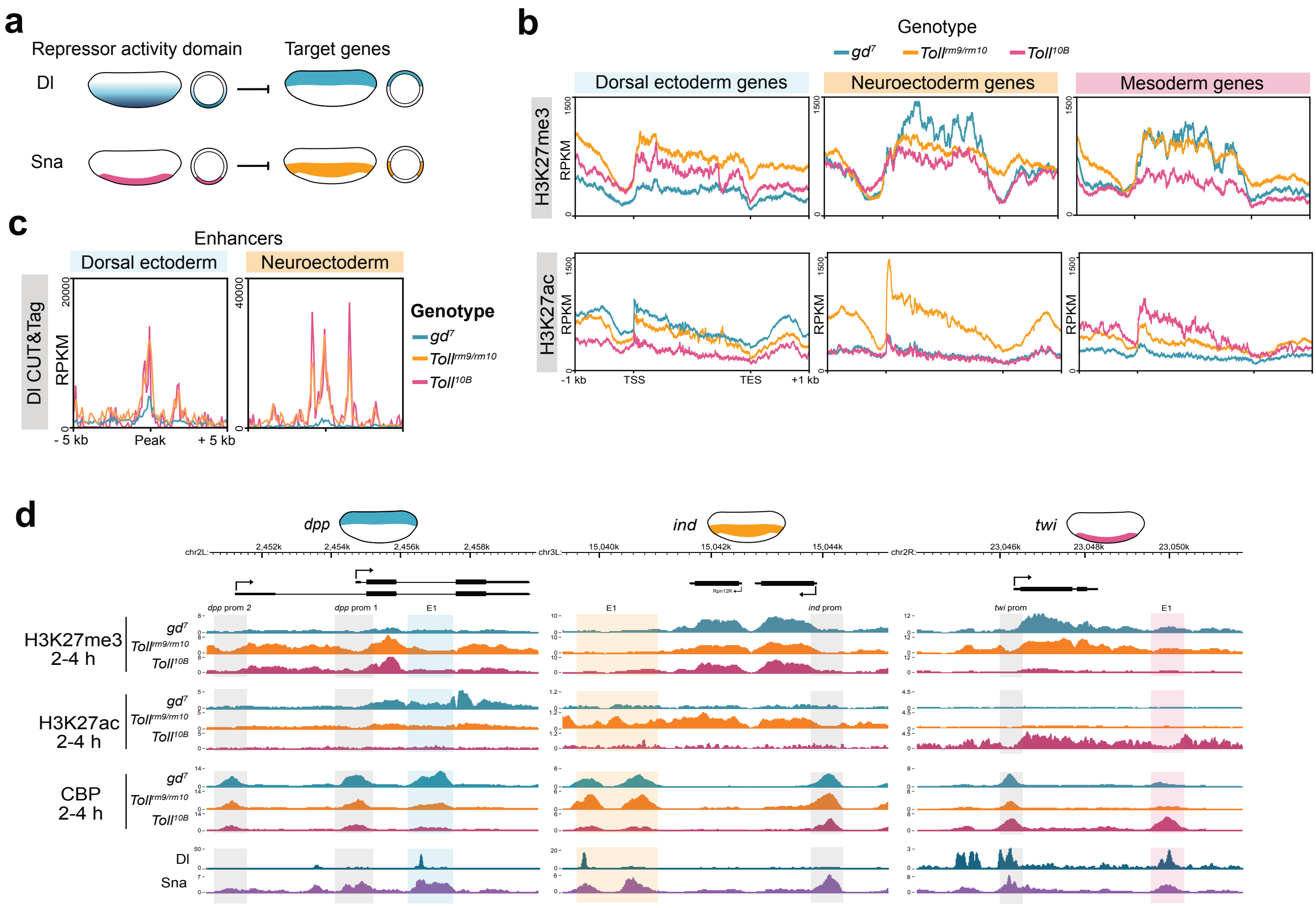
a
Repressor activity domain
Target genes
Dl
Sna
b
Genotype
gd7
Toll rm9/rm10
Toll 10B
Dorsal ectoderm genes
Neuroectoderm genes
Mesoderm genes
H3K27me3
H3K27ac
RPKM
-1 kb
TSS
TES
+1 kb
c
Enhancers
Dorsal ectoderm
Neuroectoderm
Dl CUT&Tag
RPKM
- 5 kb
Peak
+ 5 kb
Genotype
d
dpp
ind
twi
H3K27me3 2-4 h
H3K27ac 2-4 h
CBP 2-4 h
Dl
Sna
dpp prom 2
dpp prom 1
E1
ind prom
twi prom

Figure S5. Distinct repressors define the expression boundaries of DV regulated gene. a) Schematic of the repressor activity domains and target genes of Dorsal (Dl) and Snail (Sna)-mediated repression. **b)** Metagene plots of *Toll* mutant (2-4 h AEL) H3K27me3 and H3K27ac ChIP-seq signal (RPKM) at dorsal ectoderm, neuroectoderm and mesoderm-specific DV genes [15] [14]. **c)** Metagene plots (± 5 kb of CBP peak) of Dl CUT&Tag signal (RPKM) in *Toll* mutant (2-4 h AEL) embryos at dorsal ectoderm and neuroectoderm enhancers. **d)** Genome browser shots of *Toll* mutant H3K27me3, H3K27ac and CBP ChIP-seq signal alongside Dl ChIP-nexus [100] and Sna ChIP-seq [15] [14] from wild-type (2-4 h AEL) embryos at *dpp*, *ind* and *twi*.

Figure S6

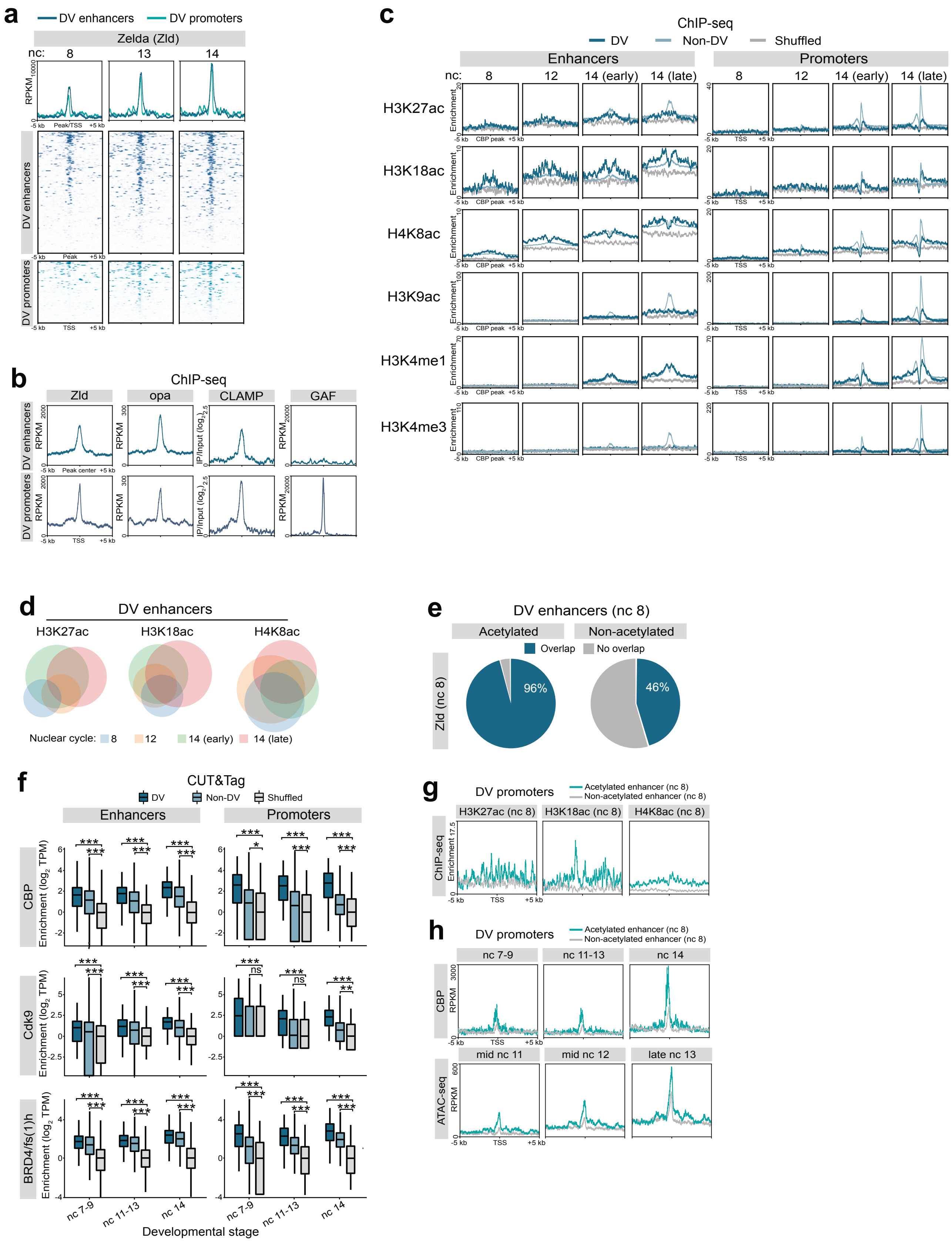
a
DV enhancers
DV promoters
Zelda (Zld)
nc: 8 13 14
RPKM
DV enhancers
DV promoters
b
ChIP-seq
Zld
opa
CLAMP
GAF
c
ChIP-seq
DV
Non-DV
Shuffled
Enhancers
Promoters
nc: 8 12 14 (early) 14 (late)
H3K27ac
H3K18ac
H4K8ac
H3K9ac
H3K4me1
H3K4me3
Enrichment
d
DV enhancers
H3K27ac
H3K18ac
H4K8ac
Nuclear cycle: 8 12 14 (early) 14 (late)
e
DV enhancers (nc 8)
Acetylated
Non-acetylated
Overlap
No overlap
Zld (nc 8)
96%
46%
f
CUT&Tag
DV
Non-DV
Shuffled
Enhancers
Promoters
CBP
Cdk9
BRD4/fs(1)h
Developmental stage
nc 7-9
nc 11-13
nc 14
g
DV promoters
Acetylated enhancer (nc 8)
Non-acetylated enhancer (nc 8)
H3K27ac (nc 8)
H3K18ac (nc 8)
H4K8ac (nc 8)
ChIP-seq
h
DV promoters
Acetylated enhancer (nc 8)
Non-acetylated enhancer (nc 8)
nc 7-9
nc 11-13
nc 14
CBP
mid nc 11
mid nc 12
late nc 13
ATAC-seq

Figure S6. Temporal dynamics of DV enhancer and promoter chromatin states. a) Metagene plots and heatmaps of Zelda (Zld) ChIP-seq enrichment in nc 8, 13 and 14 wild-type embryos at DV enhancers and promoters [57]. **b)** Metagene plots of ChIP-seq enrichment for the pioneer factors Zld [56], opa [58], CLAMP [60] and GAF [14, 15] from wild-type embryos at DV enhancers (± 5 kb of CBP peak) and promoters (± 5 kb of TSS). **c)** Metagene plots of the ChIP-seq enrichment of histone marks (H3K27ac, H3K18ac, H4K8ac, H3K9ac, H3K4me1 and H3K4me3) [62] at DV, non-DV and shuffled enhancers and promoters from wild-type embryos at nc 8, 12 and 14 (early and late). **d)** Venn diagrams of the overlap between DV enhancers bound by H3K27ac, H3K18ac and H4K8ac across the developmental time course. **e)** Overlap of DV enhancers acetylated (n = 48) or non-acetylated at nc 8 with Zld ChIP-seq peaks from nc 8. **f)** Boxplots of CUT&Tag enrichment ($\log_2$ TPM) of CBP, Cdk9 and BRD4/fs(1)h at DV and non-DV enhancers and promoters relative to shuffled genomic control regions from wild-type embryos at nc 7-9, 11-13 and 14. *P*-values (Wilcoxon rank-sum test) show significant enrichment compared to shuffled regions. **g-h)** Metagene plots of **(g)** CBP-catalyzed histone marks from nc 8 ChIP-seq and **(h)** CBP CUT&Tag and ATAC-seq enrichment at the promoters of DV genes linked to early acetylated or non-acetylated enhancers at nc 8.

Figure S7

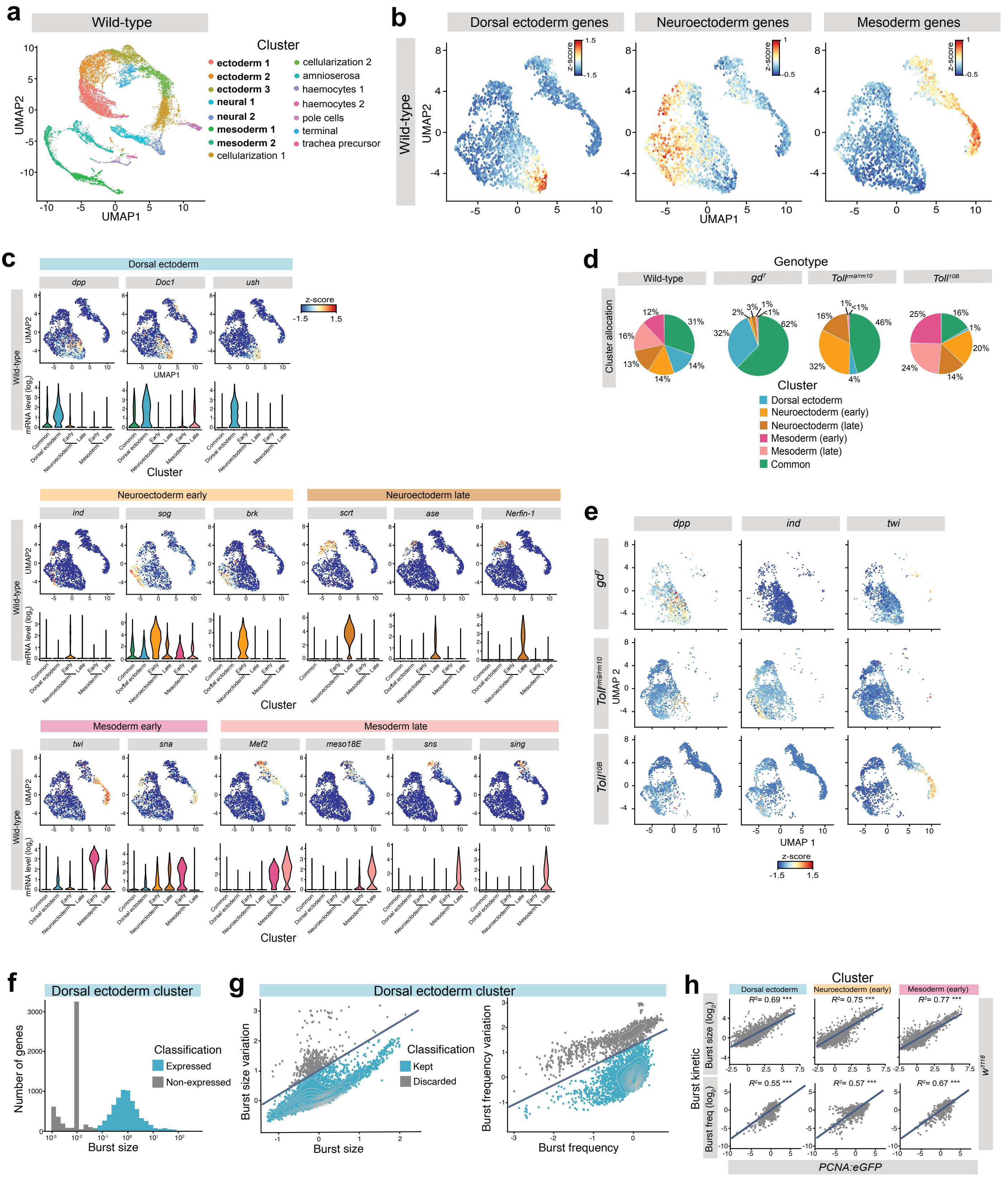
a
Wild-type
Cluster
ectoderm 1
ectoderm 2
ectoderm 3
neural 1
neural 2
mesoderm 1
mesoderm 2
cellularization 1
cellularization 2
amnioserosa
haemocytes 1
haemocytes 2
pole cells
terminal
trachea precursor
UMAP1
UMAP2
b
Dorsal ectoderm genes
Neuroectoderm genes
Mesoderm genes
Wild-type
z-score
c
Dorsal ectoderm
Neuroectoderm early
Neuroectoderm late
Mesoderm early
Mesoderm late
mRNA level (log2)
Cluster
d
Genotype
Cluster allocation
Cluster
Dorsal ectoderm
Neuroectoderm (early)
Neuroectoderm (late)
Mesoderm (early)
Mesoderm (late)
Common
e
UMAP 1
UMAP 2
f
Dorsal ectoderm cluster
Number of genes
Burst size
Classification
Expressed
Non-expressed
g
Dorsal ectoderm cluster
Burst size variation
Burst size
Burst frequency variation
Burst frequency
Classification
Kept
Discarded
h
Cluster
Dorsal ectoderm
Neuroectoderm (early)
Mesoderm (early)
Burst kinetic
Burst size (log2)
Burst freq (log2)
PCNA:eGFP

Figure S7. Identification of DV relevant cell clusters from scRNA-seq data based on PRO-seq identified DV genes. a) UMAP clustering of single-cell RNA-seq (scRNA-seq) data from wild-type embryos (2.5-3.5 h AEL) [9]. DV-relevant clusters are shown in bold, **b)** Projections of the mean expression (z-score) of dorsal ectoderm, neuroectoderm and mesoderm genes identified by PRO-seq on the UMAP of wild-type cells from DV relevant clusters from **a** reclustered according to the expression of PRO-seq DV genes (see methods). **c)** Projections of the expression of marker DV genes used to identify the 6 clusters from the UMAP from wild-type cells in **b** and violin plots of the expression (log_2 TPM) for each gene in the assigned cell clusters (see Fig. 6a). **d)** The assignment (%) of DV relevant cells from wild-type and *Toll* mutant embryo scRNA-seq UMAP clusters (see Fig. 5a). **e)** Projections of *dpp*, *ind* and *twi* expression on UMAPs from *Toll* mutant scRNA-seq (see Fig. 5a). **f)** Distribution of gene burst sizes inferred from the dorsal ectoderm cluster from wild-type scRNA-seq. A mixture model derived subpopulation of genes outside the distribution of expressed genes with low or no inferred burst sizes were considered non-expressed and removed. **g)** Inferred gene burst sizes and frequencies plotted against the variation across cells assigned to the dorsal ectoderm cluster. Genes with unreliable kinetic parameters outside the cutoff were discarded. **h)** Correlation plots of burst size (log_2) and burst frequency (log_2) for all genes passing the quality control in DV relevant (dorsal ectoderm, neuroectoderm (early) and mesoderm (early)) scRNA-seq clusters between *PCNA-eGFP* and w^{1118} control (wild-type) lines. R^2 for the correlations are denoted and associated P-values are indicated by asterisks (*** = $P < 0.001$).

Figure S8

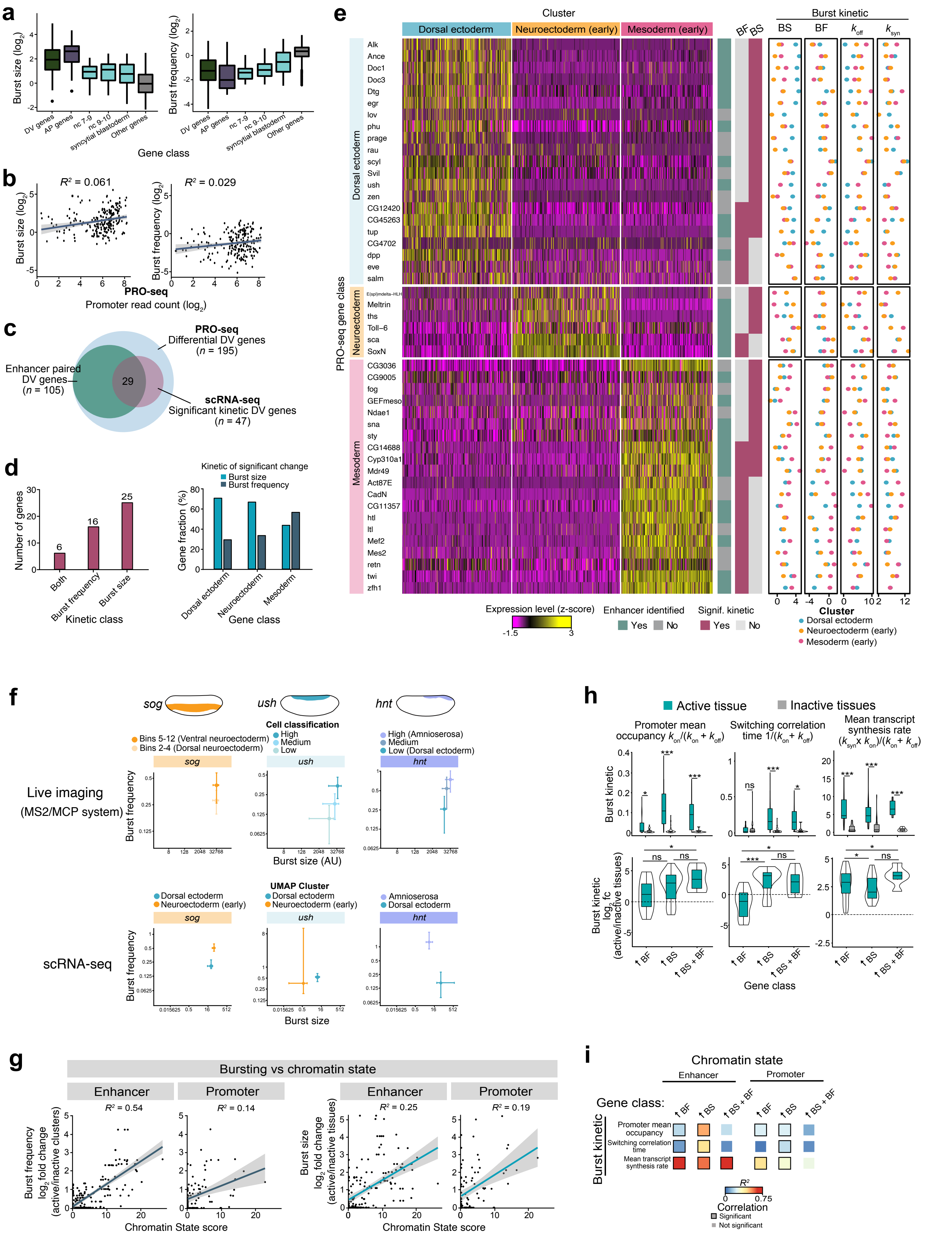

a
b
c
d
e
f
g
h
i
Gene class
PRO-seq
Promoter read count (log2)
Differential DV genes
Enhancer paired DV genes
scRNA-seq
Significant kinetic DV genes
Kinetic of significant change
Burst size
Burst frequency
Kinetic class
Cluster
Dorsal ectoderm
Neuroectoderm (early)
Mesoderm (early)
Burst kinetic
PRO-seq gene class
Expression level (z-score)
Enhancer identified
Signif. kinetic
Live imaging
(MS2/MCP system)
Cell classification
UMAP Cluster
Active tissue
Inactive tissues
Bursting vs chromatin state
Enhancer
Promoter
Chromatin State score
Chromatin state
Correlation
Significant
Not significant

Figure S8. Transcriptome-wide inference of burst kinetics from single-cell expression data. a) Boxplots of the burst size (log_2) and burst frequency (log_2) for DV and AP regulated genes, alongside genes partitioned by the stage of expression during early embryogenesis. **b)** Correlation plots of burst kinetics and the PRO-seq promoter read count (log_2) for DV regulated genes. **c)** Venn diagram showing the overlap between DV genes that are expressed in *Toll* mutant PRO-seq (n = 195), paired with enhancers (n = 105) and have a significant change in burst frequency, size or both kinetics between DV relevant clusters (n = 47). **d)** The number of DV genes with a significant kinetic change between DV-relevant clusters and the proportion (%) that change in burst size and frequency when partitioned by the tissue of expression. **e)** Heatmaps showing the scRNA-seq expression levels (z-score) of DV genes with a significant change in at least one kinetic parameter from single cells assigned to DV-relevant clusters and partitioned by the tissue of expression. Whether each DV gene changes significantly in burst size (BS) and/or burst frequency (BF) and has an identified enhancer are denoted. For each gene, the mean BF, BS, k_{on}, k_{off} and k_{syn} kinetic values from DV-relevant clusters are plotted. **f)** Plots comparing burst kinetics inferred from live imaging using MS2/MCP-GFP embryos [65, 66] with scRNA-seq-inferred transcriptional kinetics for the DV-regulated genes *sog*, *ush* and *hnt*. Error bars show the 95% confidence intervals. For the scRNA-seq-derived kinetics, clusters most comparable to the embryonic tissues examined by live imaging are shown. Note that burst size is plotted in arbitrary fluorescence units (AU) for the live imaging data, whereas it is RNAs per burst for scRNA-seq. **g)** Correlation plots of the fold change in burst kinetics (log_2 active tissue/inactive tissues) and DV enhancer and promoter tissue-specific chromatin state scores. **h)** (Top) Boxplots of additional inferred transcriptional bursting parameters for enhancer-paired DV genes, partitioned into classes based on whether they have a significant kinetic change in burst frequency (n = 8), size (n = 16) or both (n = 5) between the active and inactive tissue clusters (see **d** and **e**). (Bottom) For each class, the log_2 fold change (active/inactive tissues) is plotted. Significant differences (Wilcoxon rank-sum test) are indicated by asterisks, * = $P < 0.05$, ** = $P < 0.01$, *** = $P < 0.001$. **i)** Heatmap showing the coefficient of determination (R^2) between the chromatin state at DV enhancers and promoters compared to the burst parameterizations inferred for each class (see **h**). Comparisons with significant P-values from correlations are denoted. See Table S9 for R^2 and P-values.
