# Supplementary material for: Tissue-specific RNA Polymerase II promoter-proximal pause release and burst kinetics in a Drosophila embryonic patterning network
Source: Genome Biol. 2024 Jan 2;25:2. doi: 10.1186/s13059-023-03135-0 (PMC10763363; doi:10.1186/s13059-023-03135-0)
Supplement: Supplementary file 3 — Additional file 3: Table S3. De novo identified motifs enriched at DV promoters. [file 13059_2023_3135_MOESM3_ESM.pdf]

De novo identified motifs enriched at DV promoters

	Motif	Score	Name	Count
DV promoters	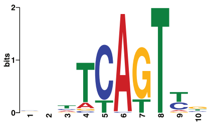	4.1e-59	Inr	133
	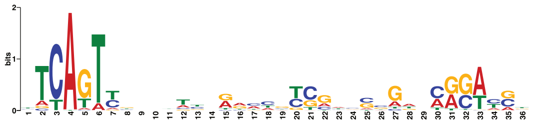	5.7e-222	Inr-DPE	165
	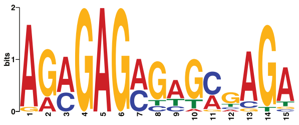	2.4e-6	GAGA	17
	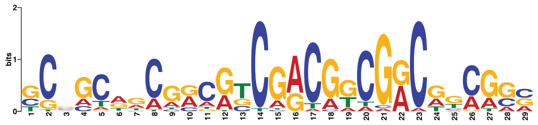	2.1e-27	Mad-Brk	24
	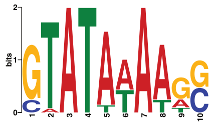	1.7e-1	TATA	21
