# Supplementary material for: Tissue-specific RNA Polymerase II promoter-proximal pause release and burst kinetics in a Drosophila embryonic patterning network
Source: Genome Biol. 2024 Jan 2;25:2. doi: 10.1186/s13059-023-03135-0 (PMC10763363; doi:10.1186/s13059-023-03135-0)
Supplement: Supplementary file 8 — Additional file 8. Peer review history. [file 13059_2023_3135_MOESM8_ESM.docx]

**Review history**

**First round of review**

**Reviewer 1**

Here the authors study the genome-wide chromatin state during Drosophila DV patterning in a tissue-specific and temporal manner by measuring histone acetylation, chromatin accessibility and CBP occupancy. They show that the chromatin state at DV enhancers, but not promoters, can predict the transcriptional output of target genes. By inferring transcription burst kinetics, DV genes are found to typically have high burst size and low burst frequency. Differences in burst kinetics between tissues correlate with differences in enhancer chromatin state. Overall, I thought the data presented and analyses are of high quality, and the findings will be of broad interest. Given the amount of data generated and published data sets analysed, the manuscript is very dense, which I found made it hard to navigate in places. My comments below largely relate to providing additional explanations to help the reader.

Major comments
Fig 3g - could the ectopic expression observed relate to higher levels of tissue specific expression in the P-TEFb OE embryos? I wonder if what looks like ectopic signal could just be much stronger amplified signal from the alkaline phosphate-based detection due to the higher expression levels? Were the embryos stained with the alkaline phosphate substrates for the same length of time? If so, I am not sure if this is appropriate given that the expression levels are different. RT-qPCR of some of the genes in the 3 mutant backgrounds with and without P-TEFb OE would be a cleaner way to provide further support for ectopic expression when P-TEFb is overexpressed.

I found the 'Repression involves exclusion of H3K27ac or induction of Polycomb-mediated H3K27me3' hard to follow. I wonder if it would be better removed from the paper but will leave this up to the authors and highlight some areas of confusion here. The authors write 'in contrast, whereas dorsal ectoderm genes are hypoacetylated in the mesoderm, H3K27ac is not completely blocked in the neuroectoderm (Fig. S5b), despite similar levels of Dl at dorsal ectoderm enhancers in these two tissues as determined by CUT&Tag (Fig. S5c)'. It would help the reader to suggest why H3K27ac might be high on dorsal ectoderm genes in the neuroectoderm if it is unrelated to Dl levels.
Then - 'prevention of H3K27ac at DV loci appears to be a major target of Sna-mediated repression' - is the evidence for this in Fig S5b that neuroectoderm genes (I presume this is what is meant by DV loci) have low H3K27ac in Tl10B embryos? The H3K27ac level of the neuroectoderm genes is the same in Tl10B and gd7 embryos, yet Sna will only be bound in Tl10B embryos, so I do not follow the logic that low H3K27ac of neuroectoderm genes is due to Sna-mediated repression.
Also, why do the authors think that neuroectoderm genes have higher H3K27me3 in Tlrm9/10 embryos than in Tl10B embryos? The neuroectoderm genes do not show the anti-correlation between expression and the H3K27me3 mark, like the dorsal ectoderm and mesoderm genes do.
Line 328 - 'both mechanisms prevent release of paused Pol II into elongation (Fig. S5d)'. What data in Fig S5d supports this statement - would this conclusion not be based on Pol II/PRO-seq reads?

Fig 5d is confusing to me. Are the genes in each class of promoter motif a mixture from the DV, AP and other categories (as in Fig S1m)? As if so, these classes will have many DV genes in them so I cannot understand why every promoter motif class (including no motif) has higher burst frequency and lower burst size than the collective set of DV genes. In any case, as the DV genes should cover a mix of different promoter types (albeit with enrichment of some particular motifs such as Inr, DPE and TATA) then surely the DV burst size and frequency should be similar to some kind of average of the frequency/sizes obtained in the individual promoter element classes rather than the extreme value for burst size and frequency? Line 449 - Since these motifs are overrepresented in the DV genes (Fig. S1j-m), it may partly explain their high burst size capacity - does this mean the combinations of Inr, DPE and TATA? Only the Inr-DPE-TATA class has a burst size similar to the DV genes.
Line 443 - The analysis revealed that DV genes have high burst sizes and low burst frequencies compared to non-DV genes …. (Fig 5c and S8a) - perhaps it is worth clarifying that AP genes have similar high burst size and low frequency to DV genes in Fig S8a.
Overall, line 455 - Thus, an enrichment of core promoter motifs that bind to TFIID may partly explain the high burst size of DV genes - I think the logic for concluding this needs to be made much clearer.

Minor comments
What is S1i supposed to show? I don't think it is referred to in the text.

S1m and Fig 5D - why is Mad-Brk included in the promoter motifs? These would be binding sites in the enhancer rather than a core promoter motif.
S2j - it would help to have greater explanation of these graphs. I would have thought a TPR of 1 would equal a FPR of zero rather than 1.

S3a, c - it is very difficult to distinguish 4h and 5h on the graphs based on the subtle color change.

S3f - based on the PRO-seq reads it looks like shn has weak expression in Tl10B and Tlrm9/10 embryos rather than being completely off. Do the authors think that there is very low level/transient expression in the tissues where the gene is off? If so is this observed for other genes? I think there is old data on tissue specific genes (eg the globins) being very weakly expressed in tissues where the gene was thought to be off.

Fig S4h - I do not understand this graph -since the y axis is ectopic expression in P-TEFb OE/WT embryos why are there 2 values for sog? I can understand that CycT occupancy will change depending on the mutants compared but not why the value for P-TEFb OE/WT embryos will be different for any given gene.

Line 340 - are the shuffled sites enhancers and promoters (eg from housekeeping genes?) or does the explanation that they are representative of genomic background mean that they are regions with neither promoter or enhancer activity?

Fig 5C - why is there transcription in the mutants in which the gene is not expressed, eg why is there data for dpp in the mesoderm/NEE or twi in the NEE/dorsal ectoderm? This relates to my query above as to whether all the genes show very low basal expression in a tissue in which they are considered to be off.

Line 509 - Interestingly, for genes changing in burst size, the enhancer chromatin state correlates well with burst size (R = 0.48) and promoter mean occupancy (kon/(kon+koff), R2 = 0.49), but not so well with burst frequency (R2 = 0.22) (Fig. 5j and S8g, h). I do not understand what the last part of this sentence (but not so well with burst frequency) means, as it is burst size that is being discussed.

Can the authors provide a list of coordinates for the eRNAs they have identified?

I found the result in Fig S6b - that Opa and CLAMP occupy DV enhancers and promoters, but GAF is only found at DV promoters - interesting but this is not really discussed further. Can the authors speculate why this might be and whether there is an advantage from GAF being absent from enhancers? Similarly, the unexpected slight increase in DV enhancer accessibility in CLAMP RNAi embryos is mentioned in passing - what could this mean?

Other results that I thought it could be interesting to speculate about the significance in the discussion are: 1)Toll10B embryos consist of 49% mesoderm cells and 34% cells that resemble neuroectoderm; 2) the changes in burst frequency associated with DV genes regulated by proximal enhancers vs changes in burst size and frequency for genes regulated by distal enhancers.

**Reviewer 2**

Hunt et al. employed a variety of whole-genome sequencing analysis combined with Drosophila genetics to study the mechanism of transcriptional regulation of dorsal-ventral patterning genes by enhancers. The authors first demonstrated that deposition of H3K27Ac by CBP at enhancers (rather than promoters) is predictive of target gene expression during early embryogenesis, which is consistent with previous studies (e.g., Koenecke et al., Genome Biol 2016). By using GAL4/UAS system, the authors further showed that overexpression of P-TEFb leads to ectopic activation of key dorsal-ventral patterning genes, suggesting that pause-releases by enhancers represents a key regulatory step in gene expression. They also reported that "enhancer priming" by Zld/CBP fosters subsequent induction of target gene expression during zygotic genome activation. Lastly, the authors attempted to infer the kinetics of transcriptional bursting using scRNA-seq data. Overall, this study provides valuable information about the regulatory mechanism of gene expression during dorsal-ventral axis formation in early embryos. I think that further experimental validation will improve the clarity of the study. Especially, inferred bursting kinetics should be experimentally validated by other methods such as single-molecule FISH analysis because current analysis shown in Fig 5 appears to be indirect and less convincing comparing to other parts of this stduy.

Specific points
1) Do the authors have any explanation why TATA is enriched for paused DV genes (Fig S1k)? As the authors may know, it has been reported that TATA is typically depleted from highly paused genes in early Drosophila embryos (e.g., Chen et al., eLife 2013, Gaertner et al., Cell Rep 2012)

2) In Page 6, it is written that "pausing is maintained in different tissue contexts, irrespective of transcription, demonstrating that pause release is a major regulatory step in tissue-specific DV transcription". This conclusion is not new as this has been demonstrated by the original pausing paper using the same Drosophila genetic background (Zeitlinger et al., Nat Genetics 2007).

3) In Page7, the authors claim that "promoter-bound CBP supports Pol II recruitment and pausing in an enzymatically-independent manner" It is not clear for me which data in the current study actually support this conclusion.

4) Fig 2h shows that enhancers are enriched with Pol II cut&tag signal but not associated with high PRO-seq signal, which led the authors to conclude that features specific to enhancers are involved in this unique Pol II profile. I wonder if the phosphorylation status of Ser5 and/or Ser2 of Pol II CTD is different between enhancers and promoters.

5) In Page 13, it is written that recruitment of CBP and CBP-catalyzed histone acetylation is associated with the "rapid" induction of tissue-specific transcription. Can the authors exclude a possibility that CBP modulates the level of gene expression without changing the timing at early and late stages of DV gene induction? Temporal kinetics of gene expression appears to be not directly examined in the current manuscript.

6) To further validate the author's model that Brd4 facilitates pause-release by recruiting PTEF-b (Fig 3A and Fig 4i), it is important to determine if overexpression of Brd4 augments transcription of DV genes as in the case of PTEF-b overexpression.

7) In Fig 3g, it has been shown that PTEF-b overexpression leads to ectopic transcriptional activation in nc14 embryos. Does this manipulation also lead to precocious activation of those genes?

8) In Fig 5f, it is inferred that each DV gene is differentially regulated by the burst size and the burst frequency. It is important to verify this conclusion by different experimental methods such as single-molecule FISH assay.

9) It is interesting to determine how PTEF-b overexpression impacts kinetics of transcriptional bursting. Given ChIP-qPCR data showing that overexpressed PTEF-b is preferentially recruited to enhancers rather than promoters (Fig S4f), this manipulation should lead to preferential increase in the burst size (as expected in Fig 5g).

**Reviewer 3**

Hunt et al. present a comprehensive analysis of gene activation across the dorsal-ventral axis of early Drosophila embryos. The study is a tour de force, in that it integrates a variety of whole-genome datasets, including H3K27Ac, ATAC-seq, CBP recruitment, and release of paused Pol II by p-TEFb. The authors also employ modeling methods to explain some of the observations, particularly regarding the control of transcriptional bursting dynamics. What makes this paper particularly compelling is that the analysis is done in the context of developing embryos, rather than more commonly used cultured cells and organoids.

The authors provide new and interesting insights into the control of zygotic genome activation (ZGA). First, they show that the recruitment of CBP to enhancers and promoters involve different mechanisms and produce distinct consequences on gene expression. CBP "primes" enhancers for transcriptional activation, while CBP helps established paused Pol II at promoters. Recruitment of p-TEFb is shown to be the key step in gene activation, by releasing Pol II from promoter-proximal pause sites.

The paper is quite exhaustive, so it is difficult to suggest any additional experiments that the authors have not already performed. I have just one small question. Does paused Pol II eventually fall away from inactive genes in tissues where they are never expressed (e.g., ind in the mesoderm)? If so, does this occur during the time when Polycomb-mediated repression is established during germband elongation?

**Authors’ response to reviewers**

**Response to reviews**

We thank the reviewers for a careful reading of the manuscript and for their useful suggestions. We are happy that they find “the data presented and analyses of high quality, and the findings of broad interest”, that it “provides valuable information about the regulatory mechanism of gene expression during dorsal-ventral axis formation in early embryos”, and “new and interesting insights into the control of zygotic genome activation”.

The reviewers also noted that the study would benefit from some clarifications, as well as validation of the burst kinetics, which we have provided in the revised manuscript and is described in our response below.

**Reviewer #1**: Here the authors study the genome-wide chromatin state during Drosophila DV patterning in a tissue-specific and temporal manner by measuring histone acetylation, chromatin accessibility and CBP occupancy. They show that the chromatin state at DV enhancers, but not promoters, can predict the transcriptional output of target genes. By inferring transcription burst kinetics, DV genes are found to typically have high burst size and low burst frequency. Differences in burst kinetics between tissues correlate with differences in enhancer chromatin state. Overall, I thought the data presented and analyses are of high quality, and the findings will be of broad interest. Given the amount of data generated and published data sets analysed, the manuscript is very dense, which I found made it hard to navigate in places. My comments below largely relate to providing additional explanations to help the reader.

Major comments

Fig 3g - could the ectopic expression observed relate to higher levels of tissue specific expression in the P-TEFb OE embryos? I wonder if what looks like ectopic signal could just be much stronger amplified signal from the alkaline phosphate-based detection due to the higher expression levels? Were the embryos stained with the alkaline phosphate substrates for the same length of time? If so, I am not sure if this is appropriate given that the expression levels are different. RT-qPCR of some of the genes in the 3 mutant backgrounds with and without P- TEFb OE would be a cleaner way to provide further support for ectopic expression when P-TEFb is overexpressed.

**Response**: We thank the reviewer for this comment and agree that it is important to distinguish between higher levels of tissue-specific expression and ectopic expression. Although the embryos were stained for the same amount of time, we do not believe that higher expression in one tissue would lead to stronger alkaline phosphatase-based staining in another tissue. Consistent with this, we did not observe ind or sog staining at the embryonic termini upon P-TEFb over-expression (Fig 3g and Figure 1 for reviewer). Therefore, we believe that ectopic expression does occur. We agree that a cleaner way to demonstrate ectopic expression would be to over- express P-TEFb in the mutant backgrounds, but this is unfortunately very difficult to achieve as it requires that Gal4, UAS-Cdk9, UAS-CycT and the Toll mutant are all present in the female germline. Instead, we have over- expressed P-TEFb in S2 cells. As can be seen in Figure 2 for the reviewer, silent but paused genes can be activated by P-TEFb overexpression whereas lowly paused genes are less affected.

control	P-TEFb OE

**Figure 1 for reviewer**. Staining for *sog* RNA in P-TEFb over-expressing embryos shows ectopic expression in the mesoderm, but not at the termini.

60

*R^2^* = 0.34

*eve*

50

*wg*

40

*hnt*

*Abd-B*

30

20

*salm*

10

*en*

*7SK*

0

*twi*

*disco*

*sog hexim RpL32*

60

*eve*

*wg*

*Abd-B hnt*

*salm*

*sna*

*disco en*

*sog*

*hexim*

*twi 7SK*

*RpL32*

mRNA fold change (P-TEFb OE/control)

mRNA fold change (P-TEFb OE/control)

50

40

30

20

10

0

1x10

-8

1x10

1x10

1x10

1x10

1x10

1x10

1x10

0	0.2	0.4	0.6	0.8	1

mRNA level in control cells (28SrRNA normalized)

-7

-6

-5

-4

-3

-2

-1

1

Pausing index (PI)

**Figure 2 for reviewer.** RT-qPCR showing upregulation of silent genes in Drosophila S2 cells when P-TEFb is overexpressed. PRO-seq derived pausing index (PI) values are from PMID: 32297950.

I found the 'Repression involves exclusion of H3K27ac or induction of Polycomb-mediated H3K27me3' hard to follow. I wonder if it would be better removed from the paper but will leave this up to the authors and highlight some areas of confusion here. The authors write 'in contrast, whereas dorsal ectoderm genes are hypoacetylated in the mesoderm, H3K27ac is not completely blocked in the neuroectoderm (Fig. S5b), despite similar levels of Dl at dorsal ectoderm enhancers in these two tissues as determined by CUT&Tag (Fig. S5c)'. It would help the reader to suggest why H3K27ac might be high on dorsal ectoderm genes in the neuroectoderm if it is unrelated to Dl levels.

**Response**: We have now tried to clarify this part of the manuscript by adding two sentences to p. 11. Dorsal ectoderm-specific genes are repressed by Dl in both the neuroectoderm and in the mesoderm. If repression involves a block to histone acetylation, we would therefore expect similar levels of H3K27ac in these two tissues. The fact that Dl occupies the dorsal ectoderm enhancers to a similar extent in the neuroectoderm and mesoderm whereas histone acetylation levels differ argues that Dl represses by some other mechanism. We speculate that these enhancers are occupied by a different set of transcription factors and chromatin regulators in the neuroectoderm than in the mesoderm.

Then - 'prevention of H3K27ac at DV loci appears to be a major target of Sna-mediated repression' - is the evidence for this in Fig S5b that neuroectoderm genes (I presume this is what is meant by DV loci) have low H3K27ac in Tl10B embrydos? The H3K27ac level of the neuroectoderm genes is the same in Tl10B and gd7 embryos, yet Sna will only be bound in Tl10B embryos, so I do not follow the logic that low H3K27ac of neuroectoderm genes is due to Sna-mediated repression.

**Response**: As correctly pointed out by the reviewer, neuroectoderm genes are repressed by different mechanisms in dorsal ectoderm and mesoderm. The Dl activator is absent from the dorsal ectoderm, whereas both Dl and Sna are present at neuroectoderm enhancers in the mesoderm. We propose that Sna prevents Dl from inducing H3K27ac in the mesoderm, clarified on p. 11 line 323, whereas the absence of activators explains the lack of H3K27ac in the dorsal ectoderm.

Also, why do the authors think that neuroectoderm genes have higher H3K27me3 in Tlrm9/10 embryos than in Tl10B embryos? The neuroectoderm genes do not show the anti-correlation between expression and the H3K27me3 mark, like the dorsal ectoderm and mesoderm genes do.

**Response**: We were also surprised to find H3K27me3 at neuroectoderm genes in Tlrm9/10 embryos where these genes are expressed, but speculate that the signal comes from the embryonic termini where neuroectoderm genes are silent (see e.g. ind in Fig. 1b).

Line 328 - 'both mechanisms prevent release of paused Pol II into elongation (Fig. S5d)'. What data in Fig S5d supports this statement - would this conclusion not be based on Pol II/PRO-seq reads?

**Response**: We thank the reviewer for pointing out this mistake. We now refer to the PRO-seq data in Fig. 1h.

Fig 5d is confusing to me. Are the genes in each class of promoter motif a mixture from the DV, AP and other categories (as in Fig S1m)? As if so, these classes will have many DV genes in them so I cannot understand why every promoter motif class (including no motif) has higher burst frequency and lower burst size than the collective set of DV genes. In any case, as the DV genes should cover a mix of different promoter types (albeit with enrichment of some particular motifs such as Inr, DPE and TATA) then surely the DV burst size and frequency should be similar to some kind of average of the frequency/sizes obtained in the individual promoter element classes rather than the extreme value for burst size and frequency? Line 449 - Since these motifs are overrepresented in the DV genes (Fig. S1j-m), it may partly explain their high burst size capacity - does this mean the combinations of Inr, DPE and TATA? Only the Inr-DPE-TATA class has a burst size similar to the DV

genes.

**Response**: The data in Fig. 5D uses all expressed genes with bursting values (n=2291), which is clarified on p. 15, and compares it to the 195 DV genes identified by PRO-seq. This shows that there are features in addition to the known core promoter motifs in the DV genes that result in higher burst size and lower burst frequency than for other genes. Still, on line 456 we suggest that the high prevalence of these core promoter motifs contribute to their burst characteristics.

Line 443 - The analysis revealed that DV genes have high burst sizes and low burst frequencies compared to non-DV genes …. (Fig 5c and S8a) - perhaps it is worth clarifying that AP genes have similar high burst size and low frequency to DV genes in Fig S8a.

**Response**: We have now added to p. 15 that also AP genes have these features.

Overall, line 455 - Thus, an enrichment of core promoter motifs that bind to TFIID may partly explain the high burst size of DV genes - I think the logic for concluding this needs to be made much clearer.

**Response**: We have removed TFIID from this sentence. Minor comments

What is S1i supposed to show? I don't think it is referred to in the text.

**Response**: S1i demonstrates that although DV gene transcription is not detected at this stage, we do observe transcription of genes that are active before cellularization.

S1m and Fig 5D - why is Mad-Brk included in the promoter motifs? These would be binding sites in the enhancer rather than a core promoter motif.

**Response**: These motifs were discovered by searching for de novo motifs in DV gene promoters, but since they don’t add much information we have removed them from Figs S1m,n and 5D.

S2j - it would help to have greater explanation of these graphs. I would have thought a TPR of 1 would equal a FPR of zero rather than 1.

**Response**: These graphs are explained in the Methods, on p. 31. It is an AUC (Area Under Curve) ROC (Reciever Opterater Characteristics) curve. A ROC curve illustrates how well a binary classification model separates classes by plotting true positives (TPR) against false positives (FPR) at different thresholds. It helps evaluate model performance and threshold selection.

Our model uses data from ATAC-seq, CBP and H2K27ac and the combination of the three to separate known DV enhancers (positive set) from other enhancers (negative set). As explained on p. 31, the positive set consists of genomic regions overlapping validated DV enhancers, whereas the negative set are CBP peaks that could not be assigned to DV genes. A ROC curve can visualise what the TPR is, i.e. how large a fraction of the known enhancers we find, and the FPR is, i.e. how large fraction of the other enhancers we need to include to get to that TPR, at any point on the curve. Preferably a ROC curve should have as large TPR as possible compared to the FPR.

This plot tells us that our model to identify DV enhancers in a set of other enhancers is good (since we have it in the top left corner, i.e. high TPR and low FPR) but not so good for promoters. In fact it tells us that we should not expect to find more than half of the known DV promoters using chromatin state data without accepting that we will also include other promoters (since the curve starts to deviate to the right around TPR of 0.5). It also shows that when we use the model with the combination of all the chromatin state data we get the best predictive power of the model since we always get the best TPR given any value of the FPR.

S3a, c - it is very difficult to distinguish 4h and 5h on the graphs based on the subtle color change.

**Response**: We changed the colors in S3a and c.

S3f - based on the PRO-seq reads it looks like shn has weak expression in Tl10B and Tlrm9/10 embryos rather than being completely off. Do the authors think that there is very low level/transient expression in the tissues where the gene is off? If so is this observed for other genes? I think there is old data on tissue specific genes (eg the globins) being very weakly expressed in tissues where the gene was thought to be off.

**Response**: We do detect low levels of transcription in the OFF tissues by both PRO-seq and RNA-seq. This is the basis for inferring burst kinetic differences between the three cell types.

Fig S4h - I do not understand this graph -since the y axis is ectopic expression in P-TEFb OE/WT embryos why are there 2 values for sog? I can understand that CycT occupancy will change depending on the mutants compared but not why the value for P-TEFb OE/WT embryos will be different for any given gene.

**Response**: Since sog is expressed in the neuroectoderm, we can use its expression pattern to define the mesoderm and dorsal ectoderm. Therefore, we have counted the occurrence of ectopic sog expression in dorsal ectoderm and mesoderm separately. The y-axis shows how frequent the ectopic expression occurs relative to wt embryos, demonstrating that sog can be detected more often in the mesoderm than in the dorsal ectoderm upon P-TEFb OE and that this correlates with higher CycT occupancy. A sentence explaining that sog was counted in the two inactive tissues has been added to the Figure legend on p. 53.

Line 340 - are the shuffled sites enhancers and promoters (eg from housekeeping genes?) or does the explanation that they are representative of genomic background mean that they are regions with neither promoter or enhancer activity?

**Response**: The shuffled sites are randomly selected genomic regions of equal size and numbers to the enhancers and promoters.

Fig 5C - why is there transcription in the mutants in which the gene is not expressed, eg why is there data for dpp in the mesoderm/NEE or twi in the NEE/dorsal ectoderm? This relates to my query above as to whether all the genes show very low basal expression in a tissue in which they are considered to be off.

**Response**: Low levels of expression can be detected in cells where the genes are not active, both by scRNA-seq using wt embryos (Fig. 5) and by PRO-seq in mutant embryos.

Line 509 - Interestingly, for genes changing in burst size, the enhancer chromatin state correlates well with burst size (R = 0.48) and promoter mean occupancy (kon/(kon+koff), R2 = 0.49), but not so well with burst frequency (R2 = 0.22) (Fig. 5j and S8g, h). I do not understand what the last part of this sentence (but not so well with burst frequency) means, as it is burst size that is being discussed.

**Response**: This is referring to the class of genes where burst size differs between cell types. Although enhancer chromatin state generally correlates with burst frequency (Fig. 5D), we find that this is not always the case. At genes with significant changes in burst size, the difference in enhancer chromatin state between mutants correlates better with burst size differences between cell types than with burst frequency differences. The sentence has been changed to “but not so well with burst frequency differences” p. 17.

Can the authors provide a list of coordinates for the eRNAs they have identified?

**Response**: We have added a column to Table S4 showing from which DV enhancers eRNAs can be detected. The coordinates of these enhancers are also shown.

I found the result in Fig S6b - that Opa and CLAMP occupy DV enhancers and promoters, but GAF is only found at DV promoters - interesting but this is not really discussed further. Can the authors speculate why this might be and whether there is an advantage from GAF being absent from enhancers? Similarly, the unexpected slight increase in DV enhancer accessibility in CLAMP RNAi embryos is mentioned in passing - what could this mean?

**Response**: We have no good explanation for why GAF is absent from DV enhancers. We speculate that GAGA motif composition differs between DV enhancers and promoters and that the presence of GAF and CBP at DV promoters contributes to their high pausing, as previously demonstrated (PMID: 29056321). The increase in DV enhancer accessibility after CLAMP RNAi could possibly be an indirect effect.

Other results that I thought it could be interesting to speculate about the significance in the discussion are:

1) Toll10B embryos consist of 49% mesoderm cells and 34% cells that resemble neuroectoderm; 2) the changes in burst frequency associated with DV genes regulated by proximal enhancers vs changes in burst size and frequency for genes regulated by distal enhancers.

**Response**: We have now included these points in the Discussion, p.18 and 19.

**Reviewer #2**: Hunt et al. employed a variety of whole-genome sequencing analysis combined with Drosophila genetics to study the mechanism of transcriptional regulation of dorsal-ventral patterning genes by enhancers. The authors first demonstrated that deposition of H3K27Ac by CBP at enhancers (rather than promoters) is predictive of target gene expression during early embryogenesis, which is consistent with previous studies (e.g., Koenecke et al., Genome Biol 2016). By using GAL4/UAS system, the authors further showed that overexpression of P-TEFb leads to ectopic activation of key dorsal-ventral patterning genes, suggesting that pause-releases by enhancers represents a key regulatory step in gene expression. They also reported that "enhancer priming" by Zld/CBP fosters subsequent induction of target gene expression during zygotic genome activation. Lastly, the authors attempted to infer the kinetics of transcriptional bursting using scRNA-seq data.

Overall, this study provides valuable information about the regulatory mechanism of gene expression during dorsal-ventral axis formation in early embryos. I think that further experimental validation will improve the clarity of the study. Especially, inferred bursting kinetics should be experimentally validated by other methods such as single-molecule FISH analysis because current analysis shown in Fig 5 appears to be indirect and less convincing comparing to other parts of this stduy.

Specific points

1) Do the authors have any explanation why TATA is enriched for paused DV genes (Fig S1k)? As the authors may know, it has been reported that TATA is typically depleted from highly paused genes in early Drosophila embryos (e.g., Chen et al., eLife 2013, Gaertner et al., Cell Rep 2012)

**Response**: We agree with the reviewer that it is unexpected to find TATA motifs in highly paused genes. However, as can be seen in Fig S1k, the Inr motif is much more prevalent (68%) in DV promoters compared to the TATA (15%) motif. Furthermore, only 7 of 192 DV genes have a TATA box without an Inr, whereas 38 genes have both motifs. It is thus very rare to find TATA without an element associated with high pausing. We refer to studies that reported depletion of TATA from highly paused genes in the Discussion, p. 18.

2) In Page 6, it is written that "pausing is maintained in different tissue contexts, irrespective of transcription, demonstrating that pause release is a major regulatory step in tissue-specific DV transcription". This conclusion is not new as this has been demonstrated by the original pausing paper using the same Drosophila genetic background (Zeitlinger et al., Nat Genetics 2007).

**Response**: We have changed this sentence to “confirms that pause release…” and added the reference to Julia Zeitlinger’s original finding.

3) In Page7, the authors claim that "promoter-bound CBP supports Pol II recruitment and pausing in an enzymatically-independent manner" It is not clear for me which data in the current study actually support this conclusion.

**Response**: We have removed “in an enzymatically-independent manner” and replaced this with “without inducing H3K27ac (Fig. 2f)”.

4) Fig 2h shows that enhancers are enriched with Pol II cut&tag signal but not associated with high PRO-seq signal, which led the authors to conclude that features specific to enhancers are involved in this unique Pol II profile. I wonder if the phosphorylation status of Ser5 and/or Ser2 of Pol II CTD is different between enhancers and promoters.

**Response**: We have performed CUT&Tag for the Ser5- and Ser2-phosphorylated Pol II CTD marks, but did not detect obvious differences in the signal of these marks between enhancers and promoters, see Figure 3 for reviewer. We speculate that this may relate to these phosphorylated forms of Pol II displaying similar distributions along genes during ZGA, as was recently shown in mammals (PMID: 36577375).

**Genotype**

 *gd^7^* *Tollrm9/rm10* *Toll10B*

Promoters	Enhancers

Dorsal ectoderm

Dorsal ectoderm

Neuroectoderm

Mesoderm

PRO-seq

RPKM

RPKM

2500 0

850

2500 0

700

850

2500 0

RPKM

RPKM

2500 0

850

700

2500 0

2500 0

850

Neuroectoderm

Mesoderm

- 5kb

2SerP CUT&Tag 5SerP CUT&Tag Rpb3 CUT&Tag

RPKM 4000

RPKM

0

0

4000 0

4000

0

0

4000 0

RPKM 4000

RPKM

0

0

4000

0

0

4000 0

4000

0

4000 0

TSS

+ 5kb

0

0

4000 0

4000

- 5kb

Peak

+ 5kb

0

4000 0

**Figure 3 for reviewer**. CUT&Tag data for Rpb3, Ser 5- and Ser 2-phosphorylated Pol II do not differ much between promoters and enhancers, whereas PRO-seq data does.

5) In Page 13, it is written that recruitment of CBP and CBP-catalyzed histone acetylation is associated with the "rapid" induction of tissue-specific transcription. Can the authors exclude a possibility that CBP modulates the level of gene expression without changing the timing at early and late stages of DV gene induction? Temporal kinetics of gene expression appears to be not directly examined in the current manuscript.

**Response**: We thank the reviewer for this comment and agree that CBP may modulate the level of gene expression without changing the timing of expression. Although expression at 2.5-3h is stronger for genes associated with histone acetylation at nc8 than for genes with non-acetylated enhancers, and this effect is much weaker at 4.5-5h (Fig 4h), this could be due to higher levels of induction rather than a more rapid induction. We have therefore changed the text in the paragraph on p.12-13 from “rapid” to “strong”.

6) To further validate the author's model that Brd4 facilitates pause-release by recruiting PTEF-b (Fig 3A and Fig 4i), it is important to determine if overexpression of Brd4 augments transcription of DV genes as in the case of PTEF-b overexpression.

**Response**: We agree that this would be an interesting experiment. Unfortunately, the reagents for performing the experiment are not available as there are no UAS-Brd4/fs(1)h flies described.

7) In Fig 3g, it has been shown that PTEF-b overexpression leads to ectopic transcriptional activation in nc14 embryos. Does this manipulation also lead to precocious activation of those genes?

**Response**: We have examined whether P-TEFb overexpression leads to precocious activation by counting stained embryos at nuclear cycles 9-12. As shown in the new Fig.S4h, we do detect precocious activation of dpp and sna, whereas the effect on twi is not statistically significant. This result has been added to the Results on p. 10.

8) In Fig 5f, it is inferred that each DV gene is differentially regulated by the burst size and the burst frequency. It is important to verify this conclusion by different experimental methods such as single-molecule FISH assay.

**Response**: We agree that validating the burst kinetics by an orthogonal method is important. For this purpose, we have examined published live imaging data from embryos where MS2 loops have been inserted into the endogenous loci of sog, hnt and ush and combined with MCP-GFP to follow transcription over time using fluorescence microscopy, published by the Ashe lab (PMID: 37060568 and PMID: 32758422). Since neither smFISH nor live imaging is sensitive enough to detect signal in cells where the gene is OFF, we cannot directly compare the change in burst kinetics between cell types as we do when we infer kinetic data from scRNA-seq.

However, we do find that burst frequency inferences in the tissue of expression are highly concordant between methods (Fig. 4 for reviewer).

**Figure 4 for reviewer**. The inferred burst frequencies from scRNA-seq and live imaging of genes in their tissue of expression are highly similar.

Further comparison between methods shows that changes of burst kinetics between different areas with different expression levels within the tissues examined by live-imaging are consistent with kinetic changes between scRNA-seq clusters (new Fig. S8f). For example, according to the live imaging data, the burst size (measured in arbitrary fluorescent units) of sog remains constant in dorsal and ventral areas of its neuroectoderm expression domain, whereas the burst frequency increases ventrally. Consistent with this, the scRNA-seq inferered kinetics find that sog burst size (RNAs per burst) is similar between neuroectoderm and dorsal ectoderm, but burst frequency is significantly higher in the neuroectoderm. Both methods therefore find that increases in the frequency of bursts underly high sog transcription. Moreover, both methods show similar kinetic changes underlying increases in ush (increases in both burst size and frequency) and hnt (predominantly burst frequency dependent) transcription. Overall, the comparisons are largely consistent with our conclusion that DV genes can be regulated by changes in either burst size or frequency.

9) It is interesting to determine how PTEF-b overexpression impacts kinetics of transcriptional bursting. Given ChIP-qPCR data showing that overexpressed PTEF-b is preferentially recruited to enhancers rather than promoters (Fig S4f), this manipulation should lead to preferential increase in the burst size (as expected in Fig 5g).

**Response**: We agree that this is an interesting experiment for a follow-up study. We feel that performing scRNA- seq on P-TEFb over-expressing embryos is outside the scope of the present manuscript.

**Reviewer #3**: Hunt et al. present a comprehensive analysis of gene activation across the dorsal-ventral axis of early Drosophila embryos. The study is a tour de force, in that it integrates a variety of whole-genome datasets, including H3K27Ac, ATAC-seq, CBP recruitment, and release of paused Pol II by p-TEFb. The authors also employ modeling methods to explain some of the observations, particularly regarding the control of transcriptional bursting dynamics. What makes this paper particularly compelling is that the analysis is done in the context of developing embryos, rather than more commonly used cultured cells and organoids.

The authors provide new and interesting insights into the control of zygotic genome activation (ZGA). First, they show that the recruitment of CBP to enhancers and promoters involve different mechanisms and produce distinct consequences on gene expression. CBP "primes" enhancers for transcriptional activation, while CBP helps established paused Pol II at promoters. Recruitment of p-TEFb is shown to be the key step in gene activation, by releasing Pol II from promoter-proximal pause sites.

The paper is quite exhaustive, so it is difficult to suggest any additional experiments that the authors have not already performed. I have just one small question. Does paused Pol II eventually fall away from inactive genes in tissues where they are never expressed (e.g., ind in the mesoderm)? If so, does this occur during the time when Polycomb-mediated repression is established during germband elongation?

**Response**: We thank the reviewer for raising this question. As can be seen in Fig S1p and Table S1, the promoter counts are lower at 5h than at 3h in inactive tissues for many genes. This suggests that paused Pol II does fall away from many inactive gene promoters eventually. To investigate this further, we tracked representative DV genes over an extended developmental timespan using previously published Pol II ChIP-seq and RNA-seq data from FACS-isolated muscle tissue from the Zeitlinger lab (PMID: 23260668) (Fig. 5 for reviewer). We observed that the paused Pol II signal decayed over time from the promoters of *dpp* and *ind* across the developmental stages. However, this did not correlate with H3K27me3, which is present at these genes in the mesoderm throughout the timespan. The Zeitlinger lab (PMID: 23260668) described genes that lost, gained and maintained paused Pol II across development, suggesting that the fate of paused Pol II is gene-specific. The study also concluded that changes to paused Pol II are stage-specific, whereas Polycomb-mediated silencing is tissue-specific.

**Figure 5 for reviewer**. Promoter Pol II ChIP-seq, RNA-seq and gene body H3K27me3 signal at *dpp* and *ind* from 2-4 h *Toll10B*

mutants and FACS-isolated muscle tissue at later developmental stages (data from PMID: 23260668).

**Second round of review**

**Reviewer 1**

The authors have addressed all my concerns and I believe that the changes in response to all the reviewers' comments have made the manuscript stronger.

**Reviewer 2**

The authors have carefully addressed the reviewers' comments, and the clarity of the study has been significantly improved. Therefore, I support the speedy publication of the study.
